# Performance of GFR Estimation in Finnish Children and Adolescents

**DOI:** 10.1016/j.ekir.2026.107082

**Published:** 2026-09-17

**Authors:** Elmeri Hiltunen, Pauliina M. Mäkelä, Jukka Schildt, Kari Pulkki, Tapio Lahtiharju, Timo Jahnukainen

**Affiliations:** 1 Faculty of Medicine, University of Helsinki, Helsinki, Finland; 2 Department of Pediatric Nephrology and Transplantation, New Children’s Hospital, Helsinki University Hospital and University of Helsinki, Helsinki, Finland; 3 Department of Clinical Physiology and Nuclear Medicine, HUS Medical Imaging Center, Helsinki University Hospital and the University of Helsinki, Helsinki, Finland; 4 Department of Clinical Chemistry, HUS Diagnostic Center, Helsinki University Hospital and University of Helsinki, Helsinki, Finland

**Keywords:** children, creatinine, cystatin C, eGFR, GFR

## Abstract

**Introduction:**

The accuracy of estimating glomerular filtration rate (GFR) using endogenous biomarkers has been widely studied, and many GFR estimating equations have been proposed. In this study, we compared the performance of these equations in a Finnish population and evaluated their accuracy in different age groups and categories based on measured GFR (mGFR).

**Methods:**

Our study included patients under the age of 20 years whose GFR was measured between September 2014 and April 2025. Patient information was collected retrospectively from the Helsinki University Hospital electronic data lake. GFR was estimated with multiple equations, using either a single analyte or a combination of analytes, and was compared with mGFR in terms of bias, precision, and accuracy.

**Results:**

Our cohort consisted of 516 patients aged from 0.8 to 19.9 years with a history of solid organ transplantation, malignancy, or suspected kidney impairment. In general, creatinine-cystatin C-based combination equations exhibited better performance than single-analyte equations. Despite developed using cystatin C not calibrated to International Federation of Clinical Chemistry and Laboratory Medicine (IFCC) reference material, the Chronic Kidney Disease in Children (CKiD) (2012) combination equation had the lowest median bias (0.1 ml/min per 1.73 m^2^), interquartile range (IQR; 14.6 ml/min per 1.73 m^2^), and the highest P_10_ and P_30_ accuracies (47.3% and 94.6%, respectively). Moreover, it surpassed the P_30_ threshold of 90% in all age groups. Other combination equations showed similar median bias (between 2.6 and 4.0 ml/min per 1.73 m^2^) and P_30_ accuracy (between 86.4% and 90.1%), with overlapping 95% confidence intervals (CIs).

**Conclusion:**

Combination equations provided more accurate estimates of GFR than single-analyte equations. Selected combination equations accurately estimated GFR across all age groups, a finding that is likely population dependent.

## Graphical abstract

**Figure:**
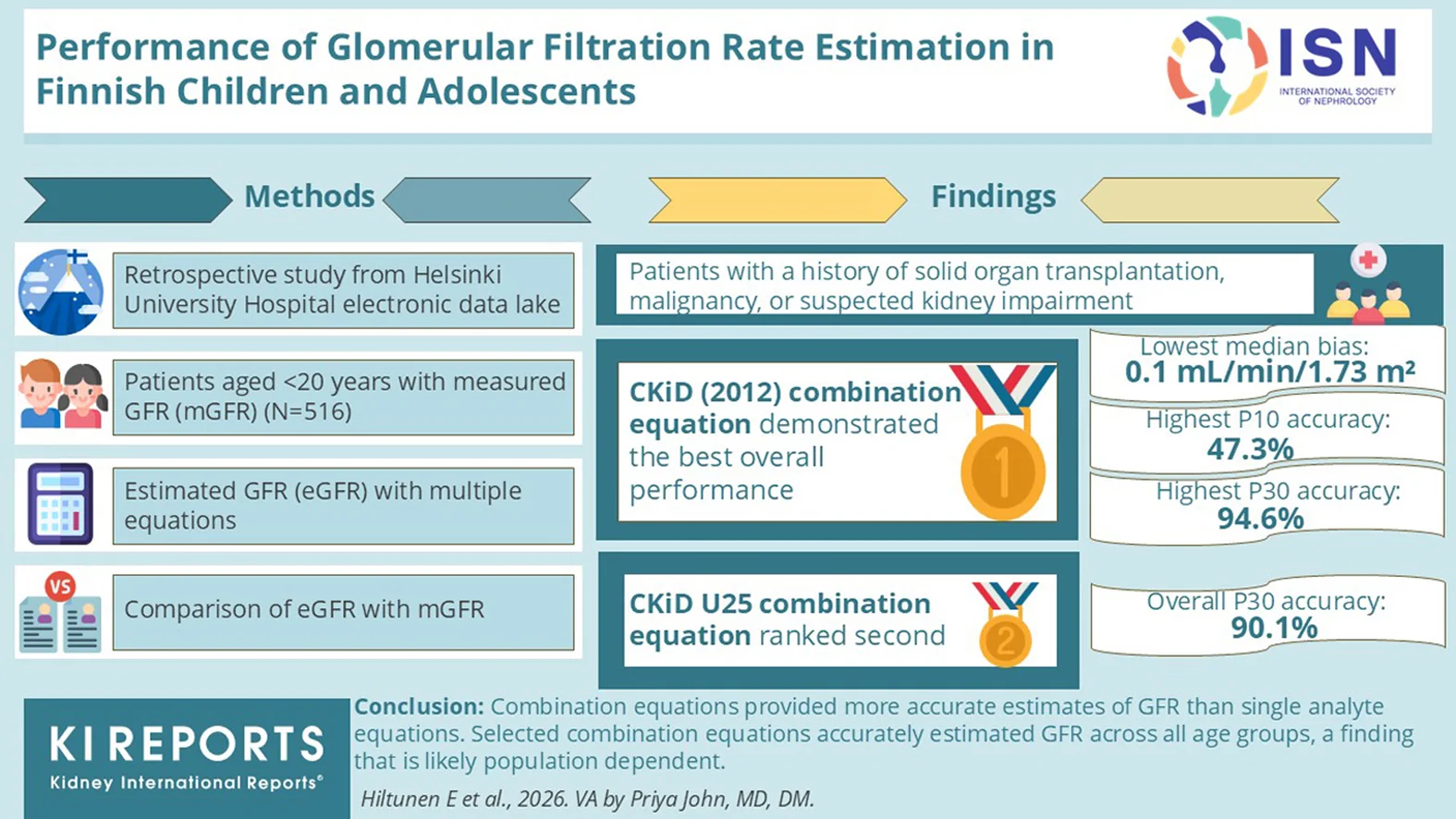

GFR is widely regarded as the most reliable indicator of kidney function. It estimates the volume of blood filtered by the glomeruli each minute, reflecting the kidneys’ ability to remove waste products and excess fluid from the bloodstream.^1^ GFR is used to evaluate and monitor kidney function, assess severity and progression of kidney disease, and guide decisions regarding prognosis and treatment. It plays a central role in the evaluation and staging of chronic kidney disease, in accordance with the classification system established by the Kidney Disease: Improving Global Outcomes guidelines.^1^ Additionally, accurate assessment of GFR is critical when determining appropriate dose of medications that are primarily excreted by the kidneys.^1^

GFR can be directly measured using exogenous filtration markers, by assessing their concentrations in blood, and less commonly in urine, over a defined period.^2^ This process typically involves i.v. administration of the marker and multiple blood samples, which may cause discomfort, particularly in pediatric patients. Alternatively, GFR can be estimated using endogenous biomarkers such as creatinine or cystatin C. This approach requires only a single blood draw, making it more patient-friendly and economical. However, these biomarkers alone do not provide an entirely accurate estimation of GFR because of confounding factors unrelated to kidney function. For instance, factors such as age, sex, and, specifically for creatinine, muscle mass can influence biomarker levels independently of GFR.^3^^,^^4^ Therefore, creatinine might be misleading in extremes of body habitus or in specific conditions, such as sarcopenia, whereas cystatin C is affected by alternative factors, such as thyroid disease or cancer.^1^

To address the variability caused by physiological development, researchers have developed GFR estimating equations that incorporate biomarker concentrations along with demographic and physiological variables (e.g., age and sex) to improve estimation accuracy. Some equations also combine multiple biomarkers to further mitigate the impact of non–GFR-related variability. In recent studies addressing the performance of pediatric GFR estimation creatinine-cystatin C-based combination equations have shown high accuracy.^5^^,^^6^ Other studies have shown that in pediatric patients, cystatin C-based equations also operate well when compared with creatinine-based equations, and in some cases, they perform as well as or even better than combination equations.^7^, ^8^, ^9^ Children below the age of 2 years are of particular interest in this study because of the limited amount of research on GFR estimation in this age group.^10^, ^11^, ^12^ Data on the reliability of eGFR in the pediatric population with subnormal kidney function are limited to studies involving relatively small patient cohorts.^5^^,^^7^^,^^8^^,^^13^ If GFR estimating equations were to achieve sufficient accuracy, eGFR would be preferred to decrease the burden of measuring GFR on pediatric patients.

The aim of this study was to evaluate the performance of GFR estimating equations in a Finnish cohort of children and adolescents, which is distinc from the populations used in the development of the equations. Additionally, we assessed the accuracy of GFR estimation across different age groups and GFR categories.

## Methods

### Data Acquisition

This study was performed as a retrospective study with data collected from the Helsinki University Hospital (HUS) electronic data lake consisting of all patients who had had their GFR measured between September 2014 and April 2025, and were under the age of 20 years. All patients had a documented history of suspected kidney disease, solid organ transplantation, or malignancy. From these records mGFR, volume of distribution, weight, height, and date of measurement were extracted. Plasma creatinine, cystatin C, and urea concentrations within 5 days of the GFR measurement were collected, and the information on date of birth, sex, diagnoses, and organ transplantation was also collected. All GFR measurements were done, and blood samples were taken and analyzed in the same accredited laboratory organization (HUS Diagnostic Center; International Organization for Standardization 15189).

The initial data consisted of 3015 measurements from 837 patients. However, the final analysis included only a patient’s first mGFR measurement with complete clinical variables, laboratory results measured with standardized laboratory methods, and an appropriate volume of distribution (20%–30%), resulting in a final number of 516 patients. This selection process is described in Figure 1. The patients in this study were not part in the development of any of the analyzed eGFR equations.

**Figure 1:**
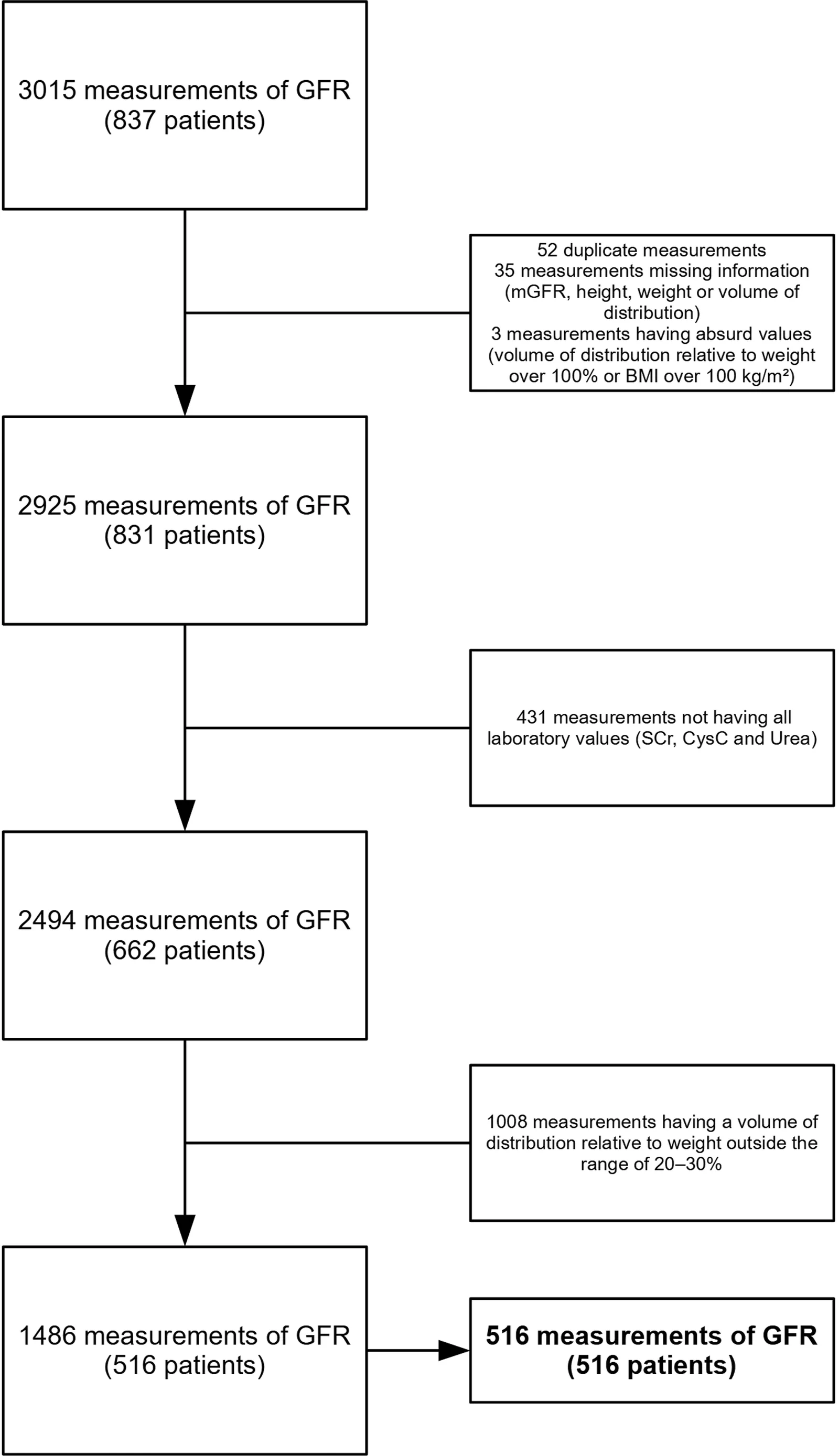
Flowchart of the data preparation procedure. The chart shows the inclusion criteria and the amount of excluded data in different steps of the process. From the remaining measurements the first measurement of each individual patient was selected, resulting in the final cohort (bold). BMI, body mass index; CysC, cystatin C; GFR, glomerular filtration rate; mGFR, measured GFR; SCr, serum creatinine.

Using the collected information on transplant operations and diagnoses, patients with a known transplant operation or an International Classification of Diseases, Tenth Revision diagnosis code Z94 before measuring GFR were counted as transplant patients. The specific organ transplanted was determined using the transplant operation data, and in the case of missing information, data obtained on diagnoses were used. Patients with International Classification of Diseases, Tenth Revision diagnosis code C00–C97 before measuring GFR were counted as patients with a malignant neoplasm.

### GFR Measurement

A mean dose of 3.7 MBq of chromium-51 ethylenediaminetetraacetic acid (2014–2019) or a mean dose of 15 MBq of technetium-99m diethylenetriaminepentaacetic acid (2019 onwards) was administered via i.v. The syringe was measured before and after injection to calculate the exact injected activity. A total of 4 serum samples (5 ml gel tubes) were obtained at 90, 150, 210, and 240 minutes after injection. The samples were either collected from the same cannula (patients <18 years) or from a secondary cannula (patients ≥18 years) as the administration of the radiotracer. A reference solution of equivalent radiopharmaceutical and volume was prepared as a standard. A 10-minute measurement with a Hidex 800 GL gammacounter (Hidex Oy, Turku, Finland) was used to determine the activity of the standard and then of the samples, twice for each time point. mGFR was determined from these results according to the slope-intercept method using the Bröchner-Mortensen correction and the volume of distribution was estimated using the Peters method. The methods of GFR measurement were based on the reference articles for children^14^ and adults.^15^

### GFR Estimation

Creatinine, cystatin C, and urea were measured from lithium-heparin plasma. The analyzers were Abbott Architect (Abbott Laboratories, Abbott Park, IL) from 2014 to 2019 and Siemens Atellica Solution (Siemens Healthineers, Erlangen, Germany) from 2019 to 2025. Creatinine was measured with isotope dilution mass spectrometry traceable enzymatic methods. Cystatin C was measured with immunochemical photometric methods that were traceable to the IFCC reference material. Urea was measured with enzymatic methods.

GFR was primarily estimated using the following equations: CKiD (2009) eGFRcr,^16^ CKiD (2012) eGFRcys,^17^ CKiD (2012) eGFRcr-cys,^17^ CKiD U25 eGFRcr,^18^ CKiD U25 eGFRcys,^18^ CKiD U25 eGFRcr-cys,^18^ European Kidney Function Consortium (EKFC) eGFRcr,^19^ EKFC-Height eGFRcr,^19^ EKFC eGFRcys,^20^^,^^21^ EKFC eGFRcr-cys,^20^ EKFC-Height eGFRcr-cys,^19^^,^^20^ r- Lund-Malmö Revised (LMR) eGFRcr,^22^^,^^23^ r-LMR eGFRcys,^23^ and r-LMR eGFRcr-cys.^23^ These are presented with more details in Supplementary Table S1, with additional equations used for supplementary analysis. Calculations were performed using the mathematical expressions shown in Supplementary Item S1. The details on the development of the supplementary equations are provided in the Supplementary References. CKiD (2012) eGFRcr-cys and CKiD (2012) eGFRcys were developed before the calibration of cystatin C assays to the IFCC reference material, but we calculated our estimations using cystatin C traceable to the IFCC reference material. All equations were extended to be used in patients between 0 and 20 years of age despite the equations not being designed to operate beyond a certain age range. Age was calculated by dividing the difference of days between the date of measuring GFR and the date of birth by 365.25.

### Statistical Analysis

To evaluate the performance of eGFR equations, we calculated the median bias (eGFR − mGFR), IQR of the difference as well as P_10_ and P_30_ accuracies for the entire data set. The P_10_ refers to the percentage of eGFR values within 10% of the mGFR value and P_30_ correspondingly to the percentage of eGFR values within 30% of the mGFR value. According to Kidney Disease: Improving Global Outcomes, the target value for eGFR P_30_ accuracy is 100% with greater than 80% being clinically acceptable and greater than 90% optimal.^1^ For median bias, IQR as well as P_10_ and P_30_ accuracies, 95% CIs were calculated using percentile bootstrapping (10^7^ resamples). The 95% CIs were used to compare different equations against each other and determine their relative performance.

We also compared the eGFR P_10_ and P_30_ accuracies in different age groups and in different mGFR categories. The age groups were assigned based on developmental factors resulting in 5 different groups (<2, 2 to <5, 5 to <10, 10 to <16, and 16 to <20 years). The mGFR categories were based on Kidney Disease: Improving Global Outcomes GFR classification,^1^ but to maintain a sufficient number of data points, these were partly combined into 4 groups (< 30, 30 to < 60, 60 to < 90, and ≥ 90 ml/min per 1.73 m^2^).

To visualize the findings, scatter plots of the relationship between mGFR and eGFR were drawn and fitted with locally weighted scatterplot smoothing to further show the difference of mGFR and eGFR in different GFR ranges. Locally weighted scatterplot smoothing is a method used to evaluate and visualize the local regression of the data. The 95% CIs of the locally weighted scatterplot smoothing regression were calculated using percentile bootstrapping (10,000 resamples).

Sensitivity analyses were performed for the effects from the difference in sampling days and from the different analyzers for creatinine and cystatin C. The difference in relative error was estimated visually with boxplots and histograms, and compared numerically with the Kruskal-Wallis test or the Wilcoxon rank-sum test.

Statistical analysis and visualization were performed with Python version, 3.12.0^24^ (Python Software Foundation, Wilmington, DE) and its packages Matplotlib (version 3.9.1.post1),^25^ NumPy (version 2.2.0),^26^ pandas (version 2.2.3),^27^^,^^28^ SciPy (version 1.14.0),^29^ seaborn (version 0.13.2),^30^ and statsmodels (version 0.14.4).^31^

### Ethics Statement

The study was approved by the HUS Medical Research Committee (§53/2024). The principles of the Declaration of Helsinki were followed throughout the study. Based on the current regulations, no informed written consent from study subjects or their caregivers is required in the case of a retrospective register-based study.

## Results

### Patient Characteristics

The patient characteristics are presented in Table 1. The median age of the cohort was 9.1 (range: 0.8–19.9) years. Of the 516 patients, 273 patients (53%) were transplant recipients with 159 having received a kidney transplant, 61 a liver transplant, 40 a heart transplant, 2 a lung transplant, and 1 a small-bowel transplant and 10 patients having undergone multiorgan transplantation. Of note, 32 patients were diagnosed with a malignant neoplasm. One patient presented with glomerular hyperfiltration (mGFR > 135 ml/min per 1.73 m^2^). All laboratory values were sampled within 5 days of mGFR, with 356 patients (69%) having creatinine and cystatin C sampled on the day of GFR measurement. In the sensitivity analysis, the difference in sampling days did not have a significant effect (*P* = 0.06) on the relative error of CKiD U25 eGFRcr-cys (Supplementary Figure S1).

**Table 1:** Demographics of the final cohort in general as well as in different age groups

Characteristic	All patients (*N* = 516)	Age < 2 yr (*n* = 72)	Age: 2–< 5 yr (*n* = 91)	Age: 5–< 10 yr (*n* = 108)	Age: 10–< 16 yr (*n* = 171)	Age: 16–< 20 yr (*n* = 74)
Female	219 (42%)	33 (46%)	38 (42%)	38 (35%)	77 (45%)	33 (45%)
Age, yr	9.1 (3.3–14.1)	1.5 (1.2–1.8)	3.0 (2.5–3.9)	7.8 (6.8–8.6)	13.2 (11.9–14.4)	17.1 (16.5–18.1)
Weight, kg	28.4 (14.1–47.9)	10.8 (9.7–11.5)	13.0 (11.4–15.0)	23.7 (21.6–27.1)	42.8 (35.0–52.5)	60.0 (52.5–68.5)
Height, cm	130 (94–158)	79 (74–81)	92 (86–96)	123 (118–128)	152 (144–163)	165 (159–173)
BMI, kg/m^2^	17.2 (15.7–19.6)	17.2 (15.9–18.5)	15.9 (14.8–16.9)	16.0 (14.8–17.4)	18.0 (16.5–20.8)	21.5 (19.2–24.0)
P-Creatinine, μmol/l	49.0 (34.0–70.0)	25.5 (19.0–37.2)	33.0 (24.5–41.0)	41.0 (34.0–52.0)	61.0 (50.0–84.0)	71.5 (60.0–87.5)
P-Cystatin C, mg/l	1.2 (1.0–1.5)	1.3 (1.1–1.8)	1.2 (1.0–1.4)	1.1 (0.9–1.3)	1.2 (1.0–1.6)	1.1 (0.9–1.3)
P-Urea, mmol/l	6.2 (4.7–8.3)	5.6 (4.0–8.1)	6.6 (5.2–8.8)	6.5 (4.9–9.1)	6.3 (4.9–8.4)	5.5 (4.3–6.9)
mGFR, ml/min per 1.73 m^2^	78 (61–93)	72 (61–89)	72 (54–82)	84 (64–97)	81 (60–92)	80 (68–97)
mGFR < 30	20 (4%)	6 (8%)	6 (7%)	2 (2%)	6 (4%)	0 (0%)
mGFR: 30– < 60	97 (19%)	11 (15%)	20 (22%)	17 (16%)	37 (22%)	12 (16%)
mGFR: 60–< 90	242 (47%)	37 (51%)	49 (54%)	46 (43%)	76 (44%)	34 (46%)
mGFR ≥ 90	157 (30%)	18 (25%)	16 (18%)	43 (40%)	52 (30%)	28 (38%)
Transplant diagnosis	273 (53%)	47 (65%)	47 (52%)	54 (50%)	90 (53%)	35 (47%)
Malignant neoplasm diagnosis	32 (6%)	4 (6%)	6 (7%)	10 (9%)	8 (5%)	4 (5%)

BMI, body mass index; mGFR, measured GFR; yr, year(s).

Age, weight, height, BMI, creatinine, cystatin C, urea, and mGFR are presented as medians with interquartile ranges in parentheses. Other characteristics are presented as the number of patients and the proportion of the patient group (all patients or an age group). Transplant and malignant neoplasm diagnoses refer to patients that have the respective diagnosis before the measuring of GFR. Transplants include kidney, liver, heart, lung, and small bowel transplants.

### Bias, Precision, and Accuracy of Selected eGFR Equations in the Entire Cohort

Median bias, IQR, and P_10_ and P_30_ accuracies for the selected eGFR equations for the entire cohort are presented in Table 2. CKiD (2012) eGFRcr-cys had the lowest median bias (0.1 [95% CI, −1.1 to 1.0] ml/min per 1.73 m^2^) and IQR (14.6 [95% CI, 13.2–16.3] ml/min per 1.73 m^2^) and the highest P_10_ and P_30_ accuracies (47.3% [95% CI, 43.0%–51.6%] and 94.6% [95% CI, 92.6%–96.5%], respectively) of all the equations. Other combination equations showed similar median bias (between 2.6 and 4.0 ml/min per 1.73 m^2^) and P_30_ accuracy, (between 86.4% and 90.1%) with overlapping CIs among themselves. The performance of selected combination equations is visualized in Figure 2.

**Table 2:** Performance of selected GFR estimating equations in the entire cohort (*N* = 516)

Equation analytes	Equation	Median bias (95% CI), ml/min per 1.73 m^2^	IQR (95% CI), ml/min per 1.73 m^2^	P_10_ (95% CI), %	P_30_ (95% CI), %
Creatinine-based equations	CKiD (2009) eGFRcr	18.5 (15.9–21.8)	31.1 (27.1–36.2)	18.6 (15.3–22.1)	51.9 (47.7–56.2)
	CKiD U25 eGFRcr	11.9 (10.1–14.9)	24.4 (21.7–27.2)	27.9 (24.0–31.8)	68.4 (64.3–72.5)
	EKFC eGFRcr	13.7 (11.9–15.1)	22.2 (19.6–24.2)	26.7 (23.1–30.6)	69.2 (65.1–73.1)
	EKFC-Height eGFRcr	10.6 (9.2–12.6)	20.1 (18.0–22.9)	31.0 (27.1–35.1)	74.8 (70.9–78.5)
	r-LMR eGFRcr	10.4 (8.8–11.6)	20.9 (18.4–23.2)	31.8 (27.7–35.9)	72.5 (68.6–76.4)
Cystatin C-based equations	CKiD (2012) eGFRcys	−14.3 (−17.0 to −11.6)	23.6 (21.8–26.0)	23.6 (20.0–27.3)	70.5 (66.7–74.4)
	CKiD U25 eGFRcys	−8.6 (−10.5 to −6.2)	23.1 (21.1–25.1)	28.1 (24.2–32.0)	80.0 (76.6–83.5)
	EKFC eGFRcys	−3.1 (−5.0 to −1.4)	23.2 (20.8–25.6)	31.4 (27.5–35.5)	80.0 (76.6–83.3)
	r-LMR eGFRcys	−5.2 (−7.1 to −3.6)	22.0 (19.8–23.8)	32.6 (28.5–36.6)	81.4 (77.9–84.7)
Combination equations	CKiD (2012) eGFRcr-cys	0.1 (−1.1 to 1.0)	14.6 (13.2–16.3)	47.3 (43.0–51.6)	94.6 (92.6–96.5)
	CKiD U25 eGFRcr-cys	2.8 (1.5–3.9)	15.7 (13.9–18.3)	45.5 (41.3–49.8)	90.1 (87.4–92.6)
	EKFC eGFRcr-cys	4.0 (3.0–5.9)	17.6 (15.6–19.4)	42.8 (38.6–47.1)	86.4 (83.3–89.3)
	EKFC-Height eGFRcr-cys	3.2 (2.1–4.0)	17.1 (15.3–19.2)	43.8 (39.5–48.1)	87.2 (84.3–89.9)
	r-LMR eGFRcr-cys	2.6 (1.0–3.7)	16.9 (15.1–18.8)	44.6 (40.3–48.8)	88.8 (86.0–91.5)

CI, confidence interval; CKiD, Chronic Kidney Disease in Children Study; CKiD (2009) eGFRcr, eGFR based on CKiD equation (2009) using creatinine; CKiD (2012) eGFRcr-cys, eGFR based on CKiD equation (2012) using creatinine, cystatin C and blood urea nitrogen; CKiD (2012) eGFRcys, eGFR based on CKiD equation (2012) using cystatin C; CKiD U25 eGFRcr, eGFR based on CKiD under 25 equation using creatinine; CKiD U25 eGFRcr-cys, eGFR based on the mean of CKiD U25 eGFRcr and CKiD U25 eGFRcys equations; CKiD U25 eGFRcys, eGFR based on CKiD under 25 equation using cystatin C; eGFR, estimated glomerular filtration rate; EKFC, European Kidney Function Consortium; EKFC eGFRcr, eGFR based on EKFC equation using creatinine and Q(age); EKFC-Height eGFRcr, eGFR based on EKFC equation using creatinine and Q(height); EKFC eGFRcr-cys, eGFR based on the mean of EKFC eGFRcr and EKFC eGFRcys equations; EKFC-Height eGFRcr-cys, eGFR based on the mean of EKFC-Height eGFRcr and EKFC eGFRcys equations; EKFC eGFRcys, eGFR based on EKFC equation using cystatin C; mGFR, mesaured glomerular filtration rate; r-LMR, Lund-Malmö Revised (LMR) equation re-expressed (r-LMR); r-LMR eGFRcr, eGFR based on r-LMR equation using SCr; r-LMR eGFRcr-cys, eGFR based on the mean of r-LMR eGFRcr and r-LMR eGFRcys equations; r-LMR eGFRcys, eGFR based on r-LMR equation using cystatin C.

Biases are presented as medians of eGFR – mGFR. Similarly, interquartile ranges are for the differences of eGFR and mGFR. The P_10_ and P_30_ values are the percentage of eGFR values within 10% and 30%, respectively, of the mGFR values.

**Figure 2:**
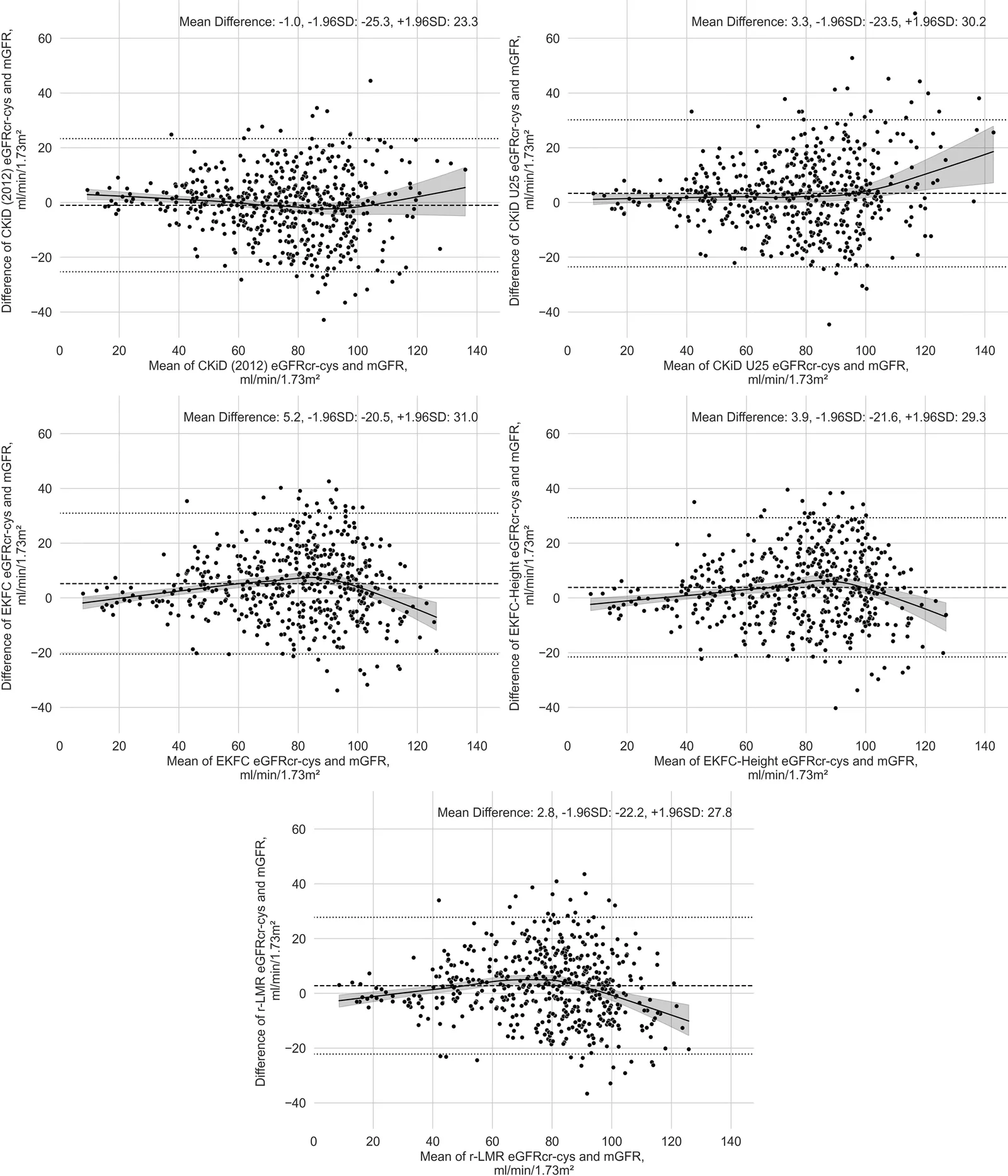
Bland-Altman plots with local regression of selected combination eGFR equations in all patients. The values are presented in ml/min per 1.73 m^2^. The gray area represents the 95% confidence interval for the locally weighted scatterplot smoothing regression (solid line) calculated using bootstrapping (10,000 resamples). The dashed line represents the mean difference of eGFR equation and mGFR (eGFR − mGFR), and the dotted lines show the ±1.96 SD from the mean difference representing the 95% limits of agreement. CKiD, Chronic Kidney Disease in Children Study; CKiD (2012) eGFRcr-cys, eGFR based on CKiD equation (2012) using creatinine, cystatin C and blood urea nitrogen; CKiD U25 eGFRcr, eGFR based on CKiD under 25 equation using creatinine; CKiD U25 eGFRcr-cys, eGFR based on the mean of CKiD U25 eGFRcr and CKiD U25 eGFRcys equations; CKiD U25 eGFRcys, eGFR based on CKiD under 25 equation using cystatin C; eGFR, estimated GFR; EKFC, European Kidney Function Consortium; EKFC eGFRcr, eGFR based on EKFC equation using creatinine and Q (age); EKFC-Height eGFRcr, eGFR based on EKFC equation using creatinine and Q (height); EKFC eGFRcr-cys, eGFR based on the mean of EKFC eGFRcr and EKFC eGFRcys equations; EKFC-Height eGFRcr-cys, eGFR based on the mean of EKFC-Height eGFRcr and EKFC eGFRcys equations; EKFC eGFRcys, eGFR based on EKFC equation using cystatin C; mGFR, measured GFR; r-LMR, Lund-Malmö Revised (LMR) equation re-expressed (r-LMR); r-LMR eGFRcr, eGFR based on r-LMR equation using creatinine; r-LMR eGFRcr-cys, eGFR based on the mean of r-LMR eGFRcr and r-LMR eGFRcys equations; r-LMR eGFRcys, eGFR based on r-LMR equation using cystatin C.

All creatinine-based equations showed positive bias and none of them, or their 95% CIs, reached the P_30_ threshold of 80% (Table 2). Of the selected creatinine-based equations, r-LMR eGFRcr had the lowest median bias of 10.4 (95% CI, 8.8–11.6) ml/min per 1.73 m^2^ and EKFC-Height eGFRcr had the highest P_30_ accuracy of 74.8% (95% CI, 70.9%–78.5%). Cystatin C-based equations exhibited in general negative bias with EKFC eGFRcys having the lowest median bias of −3.1 (95% CI, −5.0 to −1.4) ml/min per 1.73 m^2^. CKiD U25 eGFRcys, EKFC eGFRcys, and r-LMR eGFRcys had P_30_ accuracies at or above the threshold of 80%, with overlapping CIs.

### Accuracy of Selected eGFR Equations in Different Age Groups

P_10_ and P_30_ accuracies for different age groups are presented in Table 3. CKiD (2012) eGFRcr-cys had the highest P_30_ accuracy of all equations in all the age groups, ranging from 91.7% to 97.1%, except in patients over 16 years of age, in whom the highest result was achieved by CKiD U25 eGFRcr-cys, with a P_30_ accuracy of 93.2% (95% CI, 86.5%–98.6%). CKiD U25 eGFRcr-cys, EKFC-Height eGFRcr-cys, and r-LMR eGFRcr-cys surpassed the P_30_ threshold of 80% in all age groups. EKFC eGFRcr-cys had a P_30_ accuracy of at least 85.1% in patients over the age of 5, but it did not reach the P_30_ threshold of 80% in patients aged under 5 years. All combination equations presented overlapping 95% CIs with all other combination equations in all groups but CKiD (2012) eGFRcr-cys was the only equation to have CIs entirely over the P_30_ threshold of 80% in all groups. Creatinine-based equations did not reach the P_30_ threshold of 80% in any group, except for single groups with EKFC eGFRcr, EKFC-Height eGFRcr, and r-LMR eGFRcr. CKiD U25 eGFRcys and r-LMR eGFRcys reached the P_30_ threshold of 80% in patients over 2 years of age.

**Table 3:** Accuracy of selected GFR estimating equations in different age groups

Equation analytes	Equation	P_10_ (95% CI), %					P_30_ (95% CI), %				
		Age group (yr):					Age group (yr):				
		< 2 (*n* = 72)	2–< 5 (*n* = 91)	5–< 10 (*n* = 108)	10–< 16 (*n* = 171)	16–< 20 (*n* = 74)	< 2 (*n* = 72)	2–< 5 (*n* = 91)	5–< 10 (*n* = 108)	10–< 16 (*n* = 171)	16–< 20 (*n* = 74)
Creatinine-based equations	CKiD (2009) eGFRcr	9.7 (4.2–16.7)	4.4 (1.1–8.8)	15.7 (9.3–23.1)	26.9 (20.5–33.9)	29.7 (20.3–40.5)	26.4 (16.7–37.5)	26.4 (17.6–35.2)	45.4 (36.1–54.6)	68.4 (61.4–75.4)	79.7 (70.3–87.8)
	CKiD U25 eGFRcr	16.7 (8.3–25.0)	23.1 (14.3–31.9)	31.5 (23.1–40.7)	35.7 (28.7–42.7)	21.6 (12.2–31.1)	55.6 (44.4–66.7)	54.9 (45.1–64.8)	68.5 (59.3–76.9)	77.8 (71.3–83.6)	75.7 (66.2–85.1)
	EKFC eGFRcr	9.7 (4.2–16.7)	18.7 (11.0–27.5)	37.0 (27.8–46.3)	32.7 (25.7–39.8)	24.3 (14.9–33.8)	44.4 (33.3–55.6)	61.5 (51.6–71.4)	81.5 (74.1–88.9)	73.7 (67.3–80.1)	74.3 (63.5–83.8)
	EKFC-Height eGFRcr	16.7 (8.3–25.0)	24.2 (15.4–33.0)	34.3 (25.9–43.5)	39.2 (32.2–46.8)	29.7 (20.3–40.5)	58.3 (47.2–69.4)	63.7 (53.8–73.6)	82.4 (75.0–88.9)	79.5 (73.1–85.4)	82.4 (73.0–90.5)
	r-LMR eGFRcr	6.9 (1.4–13.9)	26.4 (17.6–35.2)	41.7 (32.4–50.9)	39.8 (32.7–47.4)	29.7 (20.3–40.5)	44.4 (33.3–55.6)	67.0 (57.1–76.9)	82.4 (75.0–88.9)	77.2 (70.8–83.0)	81.1 (71.6–89.2)
Cystatin C-based equations	CKiD (2012) eGFRcys	13.9 (6.9–22.2)	28.6 (19.8–38.5)	32.4 (24.1–41.7)	18.1 (12.3–24.0)	27.0 (17.6–37.8)	50.0 (38.9–61.1)	79.1 (70.3–86.8)	78.7 (70.4–86.1)	67.8 (60.8–74.9)	74.3 (63.5–83.8)
	CKiD U25 eGFRcys	9.7 (4.2–16.7)	34.1 (24.2–44.0)	31.5 (23.1–40.7)	32.7 (25.7–39.8)	23.0 (13.5–32.4)	58.3 (47.2–69.4)	84.6 (76.9–91.2)	88.0 (81.5–93.5)	80.7 (74.9–86.5)	82.4 (73.0–90.5)
	EKFC eGFRcys	18.1 (9.7–27.8)	35.2 (25.3–45.1)	37.0 (27.8–46.3)	33.3 (26.3–40.4)	27.0 (17.6–37.8)	66.7 (55.6–77.8)	76.9 (68.1–85.7)	86.1 (79.6–92.6)	83.0 (77.2–88.3)	81.1 (71.6–89.2)
	r-LMR eGFRcys	15.3 (6.9–23.6)	36.3 (26.4–46.2)	37.0 (27.8–46.3)	33.3 (26.3–40.4)	36.5 (25.7–47.3)	65.3 (54.2–76.4)	81.3 (72.5–89.0)	87.0 (80.6–92.6)	83.0 (77.2–88.3)	85.1 (77.0–93.2)
Combination equations	CKiD (2012) eGFRcr-cys	38.9 (27.8–50.0)	46.2 (36.3–56.0)	52.8 (43.5–62.0)	49.1 (41.5–56.7)	44.6 (33.8–55.4)	91.7 (84.7–97.2)	92.3 (86.8–97.8)	96.3 (92.6–99.1)	97.1 (94.2–99.4)	91.9 (85.1–97.3)
	CKiD U25 eGFRcr-cys	40.3 (29.2–51.4)	37.4 (27.5–47.3)	47.2 (38.0–56.5)	52.0 (44.4–59.6)	43.2 (32.4–54.1)	88.9 (80.6–95.8)	82.4 (74.7–90.1)	89.8 (84.3–95.4)	93.6 (89.5–97.1)	93.2 (86.5–98.6)
	EKFC eGFRcr-cys	43.1 (31.9–54.2)	36.3 (26.4–46.2)	53.7 (44.4–63.0)	45.6 (38.0–53.2)	28.4 (18.9–39.2)	77.8 (68.1–87.5)	79.1 (70.3–86.8)	90.7 (85.2–95.4)	91.8 (87.7–95.9)	85.1 (77.0–93.2)
	EKFC-Height eGFRcr-cys	41.7 (30.6–52.8)	37.4 (27.5–47.3)	54.6 (45.4–63.9)	45.6 (38.0–53.2)	33.8 (23.0–44.6)	83.3 (75.0–91.7)	80.2 (71.4–87.9)	87.0 (80.6–92.6)	92.4 (88.3–95.9)	87.8 (79.7–94.6)
	r-LMR eGFRcr-cys	38.9 (27.8–50.0)	39.6 (29.7–49.5)	51.9 (42.6–61.1)	48.5 (40.9–56.1)	36.5 (25.7–47.3)	80.6 (70.8–88.9)	85.7 (78.0–92.3)	89.8 (84.3–95.4)	92.4 (88.3–95.9)	90.5 (83.8–95.9)

CI, confidence interval; CKiD, Chronic Kidney Disease in Children Study; CKiD (2009) eGFRcr, eGFR based on CKiD equation (2009) using creatinine; CKiD (2012) eGFRcr-cys, eGFR based on CKiD equation (2012) using creatinine, cystatin C and blood urea nitrogen; CKiD (2012) eGFRcys, eGFR based on CKiD equation (2012) using cystatin C; CKiD U25 eGFRcr, eGFR based on CKiD under 25 equation using creatinine; CKiD U25 eGFRcr-cys, eGFR based on the mean of CKiD U25 eGFRcr and CKiD U25 eGFRcys equations; CKiD U25 eGFRcys, eGFR based on CKiD under 25 equation using cystatin C; eGFR, estimated GFR; EKFC, European Kidney Function Consortium; EKFC eGFRcr, eGFR based on EKFC equation using creatinine and Q(age); EKFC-Height eGFRcr, eGFR based on EKFC equation using creatinine and Q (height); EKFC eGFRcr-cys, eGFR based on the mean of EKFC eGFRcr and EKFC eGFRcys equations; EKFC-Height eGFRcr-cys, eGFR based on the mean of EKFC-Height eGFRcr and EKFC eGFRcys equations; EKFC eGFRcys, eGFR based on EKFC equation using cystatin C; r-LMR, Lund-Malmö Revised (LMR) equation re-expressed (r-LMR); r-LMR eGFRcr, eGFR based on r-LMR equation using SCr; r-LMR eGFRcr-cys, eGFR based on the mean of r-LMR eGFRcr and r-LMR eGFRcys equations; r-LMR eGFRcys, eGFR based on r-LMR equation using cystatin C.

The P_10_ and P_30_ values are the percentage of estimated GFR values within 10% and 30%, respectively, of the measured GFR values.

### Accuracy of Selected eGFR Equations in Different mGFR Categories

P_10_ and P_30_ accuracies for different mGFR categories are presented in Table 4. CKiD (2012) eGFRcr-cys and CKiD U25 eGFRcr-cys exceeded the P_30_ threshold of 90% in patients with mGFR >60 ml/min per 1.73 m^2^. CKiD (2012) eGFRcr-cys had P_30_ accuracies of 96.3% (95% CI, 93.8%–98.3%) at mGFR of 60 to < 90 ml/min per 1.73 m^2^ and 98.1% (95% CI, 95.5%–100%) at mGFR ≥ 90 ml/min per 1.73 m^2^. Both equations together with EKFC-Height eGFRcr-cys also reached the P_30_ threshold of 80% in patients with mGFR of 30 to < 60 ml/min per 1.73 m^2^. However, EKFC eGFRcr-cys was the only equation to reach the P_30_ threshold of 80% in patients with mGFR < 30 ml/min per 1.73 m^2^, with a P_30_ accuracy of 85.0% (95% CI, 70.0%–100.0%). Some single analyte equations had high P_30_ accuracies in single categories but generally had a P_30_ accuracy of under 80%.

**Table 4:** Accuracy of selected GFR estimating equations in different mGFR categories

Equation analytes	Equation	P_10_ (95% CI), %				P_30_ (95% CI), %			
		mGFR category (ml/min per 1.73 m^2^):				mGFR category (ml/min per 1.73 m^2^):			
		<30 (*n* = 20)	30–< 60 (*n* = 97)	60–< 90 (*n* = 242)	≥ 90 (*n* = 157)	< 30 (*n* = 20)	30–< 60 (*n* = 97)	60–< 90 (*n* = 242)	≥ 90 (*n* = 157)
Creatinine-based equations	CKiD (2009) eGFRcr	25.0 (10.0–45.0)	15.5 (8.2–22.7)	16.5 (12.0–21.5)	22.9 (16.6–29.9)	65.0 (45.0–85.0)	55.7 (45.4–66.0)	49.2 (43.0–55.4)	52.2 (44.6–59.9)
	CKiD U25 eGFRcr	35.0 (15.0–55.0)	24.7 (16.5–34.0)	28.5 (22.7–34.3)	28.0 (21.0–35.0)	65.0 (45.0–85.0)	71.1 (61.9–80.4)	62.8 (56.6–69.0)	75.8 (68.8–82.2)
	EKFC eGFRcr	20.0 (5.0–40.0)	19.6 (12.4–27.8)	20.7 (15.7–26.0)	41.4 (33.8–49.0)	65.0 (45.0–85.0)	60.8 (51.5–70.1)	55.0 (48.8–61.2)	96.8 (93.6–99.4)
	EKFC-Height eGFRcr	15.0 (0.0–30.0)	28.9 (19.6–38.1)	24.0 (18.6–29.3)	45.2 (37.6–52.9)	60.0 (40.0–80.0)	74.2 (64.9–82.5)	62.0 (55.8–68.2)	96.8 (93.6–99.4)
	r-LMR eGFRcr	25.0 (10.0–45.0)	24.7 (16.5–34.0)	22.7 (17.8–28.1)	51.0 (43.3–58.6)	70.0 (50.0–90.0)	53.6 (43.3–63.9)	67.4 (61.6–73.1)	92.4 (87.9–96.2)
Cystatin C-based equations	CKiD (2012) eGFRcys	45.0 (25.0–65.0)	38.1 (28.9–47.4)	25.2 (19.8–30.6)	9.6 (5.1–14.6)	65.0 (45.0–85.0)	90.7 (84.5–95.9)	74.0 (68.2–79.3)	53.5 (45.9–61.1)
	CKiD U25 eGFRcys	55.0 (35.0–75.0)	34.0 (24.7–43.3)	31.4 (25.6–37.2)	15.9 (10.2–21.7)	65.0 (45.0–85.0)	86.6 (79.4–92.8)	81.4 (76.4–86.4)	75.8 (68.8–82.2)
	EKFC eGFRcys	40.0 (20.0–60.0)	30.9 (21.6–40.2)	30.6 (24.8–36.4)	31.8 (24.8–39.5)	70.0 (50.0–90.0)	78.4 (70.1–86.6)	78.1 (72.7–83.1)	85.4 (79.6–90.4)
	r-LMR eGFRcys	45.0 (25.0–65.0)	30.9 (21.6–40.2)	35.5 (29.8–41.7)	27.4 (20.4–34.4)	75.0 (55.0–90.0)	74.2 (64.9–82.5)	82.6 (77.7–87.2)	84.7 (79.0–89.8)
Combination equations	CKiD (2012) eGFRcr-cys	40.0 (20.0–60.0)	50.5 (40.2–60.8)	46.7 (40.5–52.9)	47.1 (39.5–54.8)	75.0 (55.0–90.0)	88.7 (82.5–94.8)	96.3 (93.8–98.3)	98.1 (95.5–100.0)
	CKiD U25 eGFRcr-cys	35.0 (15.0–55.0)	46.4 (36.1–56.7)	40.9 (34.7–47.1)	53.5 (45.9–61.1)	75.0 (55.0–90.0)	85.6 (78.4–91.8)	90.1 (86.4–93.8)	94.9 (91.1–98.1)
	EKFC eGFRcr-cys	30.0 (10.0–50.0)	36.1 (26.8–45.4)	32.6 (26.9–38.4)	64.3 (56.7–72.0)	85.0 (70.0–100.0)	79.4 (71.1–86.6)	81.0 (76.0–86.0)	99.4 (98.1–100.0)
	EKFC-Height eGFRcr-cys	20.0 (5.0–40.0)	37.1 (27.8–46.4)	35.1 (29.3–41.3)	64.3 (56.7–72.0)	70.0 (50.0–90.0)	83.5 (76.3–90.7)	82.2 (77.3–86.8)	99.4 (98.1–100.0)
	r-LMR eGFRcr-cys	35.0 (15.0–55.0)	37.1 (27.8–46.4)	39.7 (33.5–45.9)	58.0 (50.3–65.6)	75.0 (55.0–90.0)	76.3 (68.0–84.5)	88.0 (83.9–92.1)	99.4 (98.1–100.0)

CI, confidence interval; CKiD, Chronic Kidney Disease in Children Study; CKiD (2009) eGFRcr, eGFR based on CKiD equation (2009) using creatinine; CKiD (2012) eGFRcr-cys, eGFR based on CKiD equation (2012) using creatinine, cystatin C and blood urea nitrogen; CKiD (2012) eGFRcys, eGFR based on CKiD equation (2012) using cystatin C; CKiD U25 eGFRcr, eGFR based on CKiD under 25 equation using creatinine; CKiD U25 eGFRcr-cys, eGFR based on the mean of CKiD U25 eGFRcr and CKiD U25 eGFRcys equations; CKiD U25 eGFRcys, eGFR based on CKiD under 25 equation using cystatin C; eGFR, estimated glomerular filtration rate; EKFC, European Kidney Function Consortium; EKFC eGFRcr, eGFR based on EKFC equation using creatinine and Q (age); EKFC-Height eGFRcr, eGFR based on EKFC equation using creatinine and Q(height); EKFC eGFRcr-cys, eGFR based on the mean of EKFC eGFRcr and EKFC eGFRcys equations; EKFC-Height eGFRcr-cys, eGFR based on the mean of EKFC-Height eGFRcr and EKFC eGFRcys equations; EKFC eGFRcys, eGFR based on EKFC equation using cystatin C; mGFR, measured GFR; r-LMR, Lund-Malmö Revised (LMR) equation re-expressed (r-LMR); r-LMR eGFRcr, eGFR based on r-LMR equation using SCr; r-LMR eGFRcr-cys, eGFR based on the mean of r-LMR eGFRcr and r-LMR eGFRcys equations; r-LMR eGFRcys, eGFR based on r-LMR equation using cystatin C.

The P_10_ and P_30_ values are the percentage of estimated GFR values within 10% and 30%, respectively, of the measured GFR values.

### Effect of Different Analyzers

In the sensitivity analysis of the effect of different analyzers, there was no significant effect on relative error with CKiD U25 eGFRcr (*P* = 0.27). However, the effect was significant with CKiD U25 eGFRcys (*P* < 0.05) and CKiD U25 eGFRcr-cys (*P* < 0.05) (Supplementary Figure S2). In the respective cohorts, CKiD U25 eGFRcr-cys had a P_30_ accuracy of 88.4% (95% CI, 85.0%–91.6%) with Abbott Architect and a P_30_ accuracy of 94.9% (95% CI, 91.2%–98.5%) with Siemens Atellica Solution. More detailed comparisons in bias, precision and accuracy for the different analyzers are presented in Supplementary Tables S2–S4.

### Remarks From the Supplementary Analysis

None of the supplementary equations had consistently higher P_30_ accuracy than the selected combination equations across the age groups or mGFR categories (Supplementary Tables S5–S7). Additional visualizations for the selected combination equations are presented in Supplementary Figures S3–S9.

We also analyzed the equations’ accuracy between patients with different diagnoses. The demographics of these patient groups are presented in Supplementary Table S8. No major differences in P_30_ accuracies could be observed between these groups when using CKiD (2012) eGFRcr-cys equation with its P_30_ accuracy ranging from 92.9% to 100% and having overlapping CIs between the groups. However, in patients with a malignant neoplasm and no prior solid organ transplantation, the highest P_10_ accuracy of all equations was 27.8% (95% CI, 11.1%–50.0%) compared with the other groups whose highest P_10_ accuracy ranged from 47.5% (95% CI, 41.3%–53.7%) to 50.0% (95% CI, 21.4%–78.6%; Supplementary Table S9).

Additionally, we analyzed the differences between sexes with the demographics presented in Supplementary Table S10, and the performance of the GFR estimating equations in Supplementary Table S11. Regarding median bias, IQR, and P_10_ and P_30_ accuracies, all selected equations presented overlapping 95% CIs between sexes.

## Discussion

The aim of our study was to evaluate the performance of GFR estimating equations in a cohort of Finnish patients under the age of 20 years and assess the accuracy of GFR estimation across different age groups and GFR categories. We found that creatinine-cystatin C-based combination equations were able to provide highly accurate estimations of mGFR with CKiD (2012) eGFRcr-cys and CKiD U25 eGFRcr-cys surpassing the P_30_ threshold of 90% for optimal equations. CKiD (2012) eGFRcr-cys was the only equation to have a P_30_ 95% CI entirely over the 90% threshold. None of the single-analyte equations achieved comparable results to the combination equations, with combination equations exhibiting lower bias, narrower IQR, and higher P_10_ and P_30_ accuracies (Table 2). Similar findings have been produced by previous studies.^5^^,^^6^

The need for relatively frequent GFR assessment is particularly important in children with progressive kidney impairment and in those with a history of solid organ transplantation or malignancy. In recent years, novel eGFR equations have been developed that demonstrate good correlation with mGFR in children with normal renal function or mild to moderate kidney impairment.^18^, ^19^, ^20^, ^21^, ^22^, ^23^ However, current knowledge regarding the accuracy of these new equations in specific patient subgroups is based on only a few studies.^7^^,^^32^, ^33^, ^34^ In our cohort, 273 patients had undergone previous solid organ transplantation and 32 had a diagnosis of malignant neoplasm at the time of GFR measurement. These groups may exhibit subnormal kidney function owing to their underlying conditions or treatments. However, GFR estimates have been reported to be less reliable in patients with a malignancy or previous transplantation.^7^^,^^32^ These factors may influence the levels of creatinine or cystatin C, and thus compromise the reliability of GFR estimation.^1^ In our analysis, the P_30_ accuracy of the best-performing CKiD (2012) eGFRcr-cys equation was comparable across all patient groups with a P_30_ accuracy ranging from 92.9% to 100% and having overlapping CIs between different groups. Patients with malignant neoplasms exhibited 21.1 percentage points lower P_10_ accuracy when comparing this group’s highest P_10_ accuracy with the mean of the other groups’ highest P_10_ accuracies (Supplementary Table S9). However, all groups had overlapping P_10_ CIs, and the differing group had only a small number of patients (*n* = 18). Therefore, we do not consider this to have significantly affected our overall results and we cannot confidently make conclusions on the performance of the evaluated eGFR formulae in patients with a malignancy.

We found combination equations to reach high accuracy, with CKiD (2012) eGFRcr-cys and CKiD U25 eGFRcr-cys reaching P_30_ accuracies of 94.6% (95% CI, 92.6%–96.5%) and 90.1% (95% CI, 87.4%–92.6%), respectively, in the entire cohort (Table 2). In a study by Roussel *et al.*,^5^ involving 307 children, CKiD (2012) eGFRcr-cys and CKiD U25 eGFRcr-cys achieved similar P_30_ accuracies of 95.4% and 91.2%, respectively. Moreover, a study by de Souza *et al.*,^35^ involving 73 pediatric kidney transplant recipients, found creatinine-cystatin C-based equations to be more accurate than single-analyte equations and found CKiD (2012) eGFRcr-cys to be the best-performing equation, with a P_30_ accuracy of 98% (95% CI, 96%–99%). Additionally, in our cohort CKiD (2012) eGFRcr-cys was optimally accurate in all age groups, even in patients under 2 years of age, and CKiD U25 eGFRcr-cys had a P_30_ accuracy of at least 82.4% in all age groups (Table 3). Moreover, Roussel *et al.* reported that CKiD (2012) eGFRcr-cys and CKiD U25 eGFRcr-cys show good performance in all age groups, with the equations showing only a small effect of age (*R*^2^ < 0.05).^5^

In our analysis, none of the single analyte equations, or their 95% CIs, reached the P_30_ threshold of 90% and no creatinine-based equation reached the P_30_ threshold of 80%. Additionally, Roussel *et al.*^5^ found creatinine-based formulas to have inadequate performance, with none of them reaching P_30_ accuracy of 80%. In a study with a multicenter cohort involving 2293 children and 1816 young adults, Nyman *et al.*^36^ found single-analyte equations CKiD U25 eGFRcr and CKiD U25 eGFRcys to be similarly accurate in children and in young adults, both surpassing the P_30_ threshold of 80% in both groups. In our analysis CKiD U25 eGFRcys performed similarly, with a P_30_ accuracy of 80.0% (95% CI, 76.6%–83.5%), but CKiD U25 eGFRcr had a P_30_ accuracy of 68.4% (95% CI, 64.3%–72.5%) in the entire cohort. Additionally, de Souza *et al.*^35^ found creatinine-based equations to be accurate, with P_30_ accuracies of over or close to 90% across different mGFR categories. We suspect that these divergent findings primarily reflect the differences between the study cohorts: The median age in our cohort was lower (9.1 [IQR, 3.3–14.1] years) than that reported by de Souza *et al.*^35^ (11.5 [IQR, 7.7–15.1] years) and Nyman *et al.*^36^ (children, 11.9 [95% distribution, 2.3–17.8] years). Therefore, the observed discrepancies in the accuracy of creatinine-based equations could be explained by our younger patient cohort because we saw CKiD U25 eGFRcr to have a P_30_ accuracy of ∼55% in patients under the age of 5 years compared with a P_30_ accuracy of > 75% in patients over the age of 10 years (Table 3). However, we cannot confirm this reasoning because these studies did not provide detailed information on the performance of GFR estimation relative to their cohort’s age distribution. Additionally in contrast to de Souza *et al.*,^35^ our cohort included not only kidney transplant recipients but also patients with other solid organ transplants which could affect the results.

CKiD (2012) eGFRcr-cys, CKiD U25 eGFRcr-cys, and EKFC-Height eGFRcr-cys presented clinically acceptable P_30_ accuracy of over 80% in patients with mGFR ≥ 30 ml/min per 1.73 m^2^. However, in patients with mGFR < 30 ml/min per 1.73 m^2^, these equations did not reach the P_30_ threshold of 80% (Table 4). We suspect that this mainly results from the higher requirement of absolute accuracy to reach the same percentile threshold with smaller values. However, we think it should be considered that this category included far fewer patients (*n* = 20) than any other category, which may have limited the credibility of the results in this category. This is shown by the wide 95% CIs in this category. Additionally, Roussel *et al.*^5^ found CKiD (2012) eGFRcr-cys to be optimally accurate both in patients with mGFR <75 ml/min per 1.73 m^2^ and in those with mGFR > 75 ml/min per 1.73 m^2^, with P_30_ accuracies of 90% and 98%, respectively. This is consistent with our results seen in Table 4.

Despite CKiD U25 eGFRcr-cys achieving optimal P_30_ accuracy of 90.1% (95% CI, 87.4%–92.6%), EKFC eGFRcr-cys, EKFC-Height eGFRcr-cys, and r-LMR eGFRcr-cys had rather similar P_30_ accuracies ranging from 86.4% to 88.8%, with overlapping 95% CIs with CKiD U25 eGFRcr-cys. Additionally, all these equations presented similar median biases (2.6–4.0 ml/min per 1.73 m^2^) and P_10_ accuracies (42.8%–45.5%), with overlapping 95% CIs (Table 2). Therefore, based on this study, we recognize the better performance of combination equations compared with single-analyte equations, but we cannot recommend any of the previously mentioned equations over another. It should also be noted that CKiD U25 eGFRcr-cys and EKFC-Height eGFRcr-cys require information on the patient’s height and, therefore, cannot be automatically reported by laboratories. EKFC eGFRcr-cys and r-LMR eGFRcr-cys do not use height as a parameter.

CKiD (2012) eGFRcr-cys was the only equation to achieve optimal P_30_ accuracy (94.6%), with a 95% CI entirely over the 90% threshold (92.6%–96.5%) in the entire cohort. CKiD (2012) eGFRcr-cys also had the lowest median bias (0.1 [95% CI, −1.1 to 1.0] ml/min per 1.73 m^2^), and it was the only equation to reach 90% P_30_ accuracy in all age groups, with the lower limits of the 95% CIs ranging from 84.7% to 94.2%. However, its use could be considered controversial, because it was developed before the calibration of cystatin C assays to the IFCC reference material. Although CKiD (2012) eGFRcr-cys has demonstrated good performance in the previous research using cystatin C measured with methods traceable to IFCC reference material, these findings could argue for its use even after calibration of cystatin C.^5^ However, to confirm this, additional studies are required. Additionally, CKiD (2012) eGFRcr-cys was the only equation to use blood urea nitrogen as a biomarker which could contribute to its higher accuracy. Therefore, we think that the use of blood urea nitrogen in combination with other biomarkers in eGFR equations should be further investigated together with calibrated creatinine and cystatin C assays.

Although we found some equations to have nearly perfect P_30_ accuracy, the P_10_ accuracy of all equations was generally under 50% (Table 2). However, this problem is not unique to pediatric patients and there is evidence suggesting an individual-level discrepancy between eGFR and mGFR when using the current eGFR equations in adults.^37^ Thirty percent variance in kidney function can be significant in clinical practice; therefore, further developments in GFR estimation are required. Future equations should aim to achieve even higher P_10_ accuracy to provide more accurate estimates of GFR.

Our study has some limitations. First, our cohort only included Finnish children and young adults that could affect the applicability of our results in other populations. Second, we could not collect information on patients’ medications or ethnicity. Third, for some patients the date of laboratory value sampling differed from the date of GFR measurement. However, we did not observe significant difference in the sensitivity analysis. Fourth, the cohort consisted of laboratory values measured on different analyzers which affected the results of cystatin C-based and combination equations (Supplementary Figure S2 and Supplementary Tables S2, S3 and S4). However, because both analyzers are traceable to the IFCC reference material and both are commonly used in practice, we consider the inclusion of results from both analyzers valuable for the clinical applicability of our results. Finally, we have based our analysis of P_30_ accuracy mainly on the thresholds of 80% and 90% suggested by the Kidney Disease: Improving Global Outcomes guidelines.^1^ Although we find these thresholds to be helpful when evaluating the clinical applicability of an equation, we acknowledge the arbitrariness of any threshold. Therefore, we have complemented our analysis by taking other metrics, such as bias, and their 95% CIs into consideration when evaluating the performance of an equation and when comparing equations against each other.

The major strength of this study was the large dataset which was independent from the data sets used to create the analyzed GFR estimating equations. Additionally, all our laboratory data was produced in the same accredited laboratory organization which minimizes the possibility of random variation in our data. We think our data set captured a realistic representation of a clinical patient setting with our minimal exclusion criteria and with that we find our results to be better applicable in actual clinical practice. Our patient cohort also included a significant number of patients under the age of 2 years who have been underrepresented in other studies.

In conclusion, we found that creatinine-cystatin C-based combination equations could accurately estimate GFR in our cohort of children and adolescents. None of the single-analyte equations achieved consistently adequate accuracy for clinical use.

## Disclosure

TL reports consultancy and speaker fees from Labquality Ltd., Aurevia Ltd. KP is an internal clinician in SGS Fimko and reports receiving speaker fee from Labquality Ltd, Finland. All the other authors declared no competing interests.

## Supplementary Material

Supplementary File (PDF)

**Supplementary References. Supplementary Item S1.** List of mathematical expressions of analyzed GFR estimating equations. **Figure S1.** Effect of the measurement date difference of laboratory values and mGFR on the relative difference of CKiD U25 eGFRcr-cys and mGFR. **Figure S2.** Effect of different analyzers on the relative difference of CKiD U25 equations and mGFR. **Figure S3.** Scatterplots with local regression of mGFR and eGFR values for CKiD (2012) eGFRcr-cys equation in all patients as well as in different age groups. **Figure S4.** Scatterplots with local regression of mGFR and eGFR values for CKiD U25 eGFRcr-cys equation in all patients as well as in different age groups. **Figure S5.** Scatterplots with local regression of mGFR and eGFR values for EKFC eGFRcr-cys equation in all patients as well as in different age groups. **Figure S6.** Scatterplots with local regression of mGFR and eGFR values for EKFC-Height eGFRcr-cys equation in all patients as well as in different age groups. **Figure S7.** Scatterplots with local regression of mGFR and eGFR values for r-LMR eGFRcr-cys equation in all patients as well as in different age groups. **Figure S8.** Scatterplots with local regression of age and difference of selected combination eGFR equations and mGFR in all patients. **Figure S9.** Scatterplots with local regression of age and relative difference of selected combination eGFR equations and mGFR in all patients. **Table S1.** GFR estimating equations, their abbreviations and parameters. **Table S2.** Performance of selected GFR estimating equations divided by the used analyzer. **Table S3.** Accuracy of selected GFR estimating combination equations in different age groups and mGFR categories divided by the used analyzer. **Table S4.** Accuracy of selected GFR estimating combination equations in different diagnosis groups and by sex divided by the used analyzer. **Table S5.** Performance of all analyzed GFR estimating equations in the entire cohort (*N* = 516). **Table S6.** Accuracy of all analyzed GFR estimating equations in different age groups. **Table S7.** Accuracy of all analyzed GFR estimating equations in different mGFR categories. **Table S8.** Demographics of the final cohort in patients with different transplant and malignant neoplasm diagnoses. **Table S9.** Accuracy of all analyzed GFR estimating equations in different diagnosis groups based on transplantations (T) and malignant neoplasm diagnoses (MN). **Table S10.** Demographics of the final cohort by sex. **Table S11.** Performance of all analyzed GFR estimating equations by sex. STARD 2015 Checklist.
