# Supplementary material for: Performance of GFR Estimation in Finnish Children and Adolescents
Source: Kidney Int Rep. 2026 Sep 17;11(12):107082. doi: 10.1016/j.ekir.2026.107082 (PMC13697211; doi:10.1016/j.ekir.2026.107082)
Supplement: Supplementary file 1 [file mmc1.pdf]

Supplementary Material

Table of contents

Supplementary Table S1. GFR estimating equations, their abbreviations and parameters ___ *2*

Supplementary Table S2. Performance of selected GFR estimating equations divided by the used analyzer ___ *3*

Supplementary Table S3. Accuracy of selected GFR estimating combination equations in different age groups and mGFR categories divided by the used analyzer ___ *4*

Supplementary Table S4. Accuracy of selected GFR estimating combination equations in different diagnosis groups and by sex divided by the used analyzer ___ *5*

Supplementary Table S5. Performance of all analyzed GFR estimating equations in the entire cohort (N = 516) ___ *6*

Supplementary Table S6. Accuracy of all analyzed GFR estimating equations in different age groups ___ *7*

Supplementary Table S7. Accuracy of all analyzed GFR estimating equations in different mGFR categories ___ *8*

Supplementary Table S8. Demographics of the final cohort in patients with different transplant and malignant neoplasm diagnoses ___ *9*

Supplementary Table S9. Accuracy of all analyzed GFR estimating equations in different diagnosis groups based on transplantations (T) and malignant neoplasm diagnoses (MN) ___ *10*

Supplementary Table S10. Demographics of the final cohort by sex ___ *11*

Supplementary Table S11. Performance of all analyzed GFR estimating equations by sex ___ *12*

Supplementary Figure S1. Effect of the measurement date difference of laboratory values and mGFR on the relative difference of CKiD U25 eGFRcr-cys and mGFR. ___ *13*

Supplementary Figure S2. Effect of different analyzers on the relative difference of CKiD U25 equations and mGFR. ___ *14*

Supplementary Figure S3. Scatterplots with local regression of mGFR and eGFR values for CKiD (2012) eGFRcr-cys equation in all patients as well as in different age groups. ___ *15*

Supplementary Figure S4. Scatterplots with local regression of mGFR and eGFR values for CKiD U25 eGFRcr-cys equation in all patients as well as in different age groups. ___ *16*

Supplementary Figure S5. Scatterplots with local regression of mGFR and eGFR values for EKFC eGFRcr-cys equation in all patients as well as in different age groups. ___ *17*

Supplementary Figure S6. Scatterplots with local regression of mGFR and eGFR values for EKFC-Height eGFRcr-cys equation in all patients as well as in different age groups. ___ *18*

Supplementary Figure S7. Scatterplots with local regression of mGFR and eGFR values for r-LMR eGFRcr-cys equation in all patients as well as in different age groups. ___ *19*

Supplementary Figure S8. Scatterplots with local regression of age and difference of selected combination eGFR equations and mGFR in all patients. ___ *20*

Supplementary Figure S9. Scatterplots with local regression of age and relative difference of selected combination eGFR equations and mGFR in all patients. ___ *21*

Supplementary Item S1. List of mathematical expressions of analyzed GFR estimating equations ___ *22*

Supplementary Item S2. STARD 2015 Checklist ___ *26*

Supplementary References ___ *29*

Supplementary Table S1. GFR estimating equations, their abbreviations and parameters

Equation	Abbreviation	Parameters
**Creatinine-based equations**		
Chronic Kidney Disease Epidemiology Collaboration (CKD-EPI) equation (2009) with adjusted SCr values in individuals under 40 (CKD-EPI40)[S1]	CKD-EPI40 eGFRcr	SCr, Age, Sex
Chronic Kidney Disease in Children (CKiD) Study equation (2009) using SCr, "Schwartz bedside"[16]	CKiD (2009) eGFRcr	SCr, Height
Chronic Kidney Disease in Children (CKiD) under 25 (U25) equation using SCr[18]	CKiD U25 eGFRcr	SCr, Age, Sex, Height
European Kidney Function Consortium (EKFC) equation using SCr, based on Q(age)[19]	EKFC eGFRcr	SCr, Age, Sex
European Kidney Function Consortium (EKFC) equation using SCr, based on Q(height)[19]	EKFC-Height eGFRcr	SCr, Age, Height
Full Age Spectrum (FAS) equation using SCr, based on Q(age)[S2,3]	FAS eGFRcr	SCr, Age, Sex
Full Age Spectrum (FAS) equation using SCr, based on Q(height)[S2,3]	FAS-Height eGFRcr	SCr, Age, Sex, Height
Flanders Metadata (FM) equation[S4]	FM eGFRcr	SCr, Age, Height
Gao quadratic (GQ) equation[S5]	GQ eGFRcr	SCr, Age, Sex, Height
Lund-Malmö revised using SCr, including aged < 18 yr (LMR18)[S6]	LMR18 eGFRcr	SCr, Age, Sex
Lund-Malmö Revised (LMR) equation re-expressed (r-LMR) using SCr, including aged < 18 yr[22,23]	r-LMR eGFRcr	SCr, Age, Sex
Schwartz-Lyon equation[S7]	Schwartz-Lyon eGFRcr	SCr, Age, Sex, Height
**Cystatin C-based equations**		
Berg equation[S8]	Berg eGFRcys	CysC
Caucasian, Asian, pediatric and adult (CAPA) equation[S9]	CAPA eGFRcys	CysC, Age
**Chronic Kidney Disease Epidemiology Collaboration (CKD-EPI) equation (2012) using CysC[S10]	CKD-EPI (2012) eGFRcys	CysC, Age, Sex
*Chronic Kidney Disease in Children (CKiD) Study equation (2012) using CysC, "Schwartz Cystatin C"[17]	CKiD (2012) eGFRcys	CysC*
Chronic Kidney Disease in Children (CKiD) under 25 (U25) equation using CysC[18]	CKiD U25 eGFRcys	CysC, Age, Sex
**European Kidney Function Consortium (EKFC) equation using CysC[20,21]	EKFC eGFRcys	CysC, Age
Full Age Spectrum (FAS) equation using CysC, based on Q(age)[S11]	FAS eGFRcys	CysC, Age
Lund-Malmö Revised (LMR) equation re-expressed (r-LMR) using cystatin C[23]	r-LMR eGFRcys	CysC, Age, Sex
**Creatinine-Cystatin C-based combination equations**		
*Chronic Kidney Disease in Children (CKiD) Study combination equation (2012) using SCr and CysC[17]	CKiD (2012) eGFRcr-cys	SCr, CysC*, BUN, Sex, Height
Mean of CKiD U25 eGFRcr and CKiD U25 eGFRcys equations[18]	CKiD U25 eGFRcr-cys	SCr, CysC, Age, Sex, Height
Mean of EKFC eGFRcr and EKFC eGFRcys[20]	EKFC eGFRcr-cys	SCr, CysC, Age, Sex
***Mean of EKFC-Height eGFRcr and EKFC eGFRcys[19,20]	EKFC-Height eGFRcr-cys	SCr, CysC, Age, Height
Full Age Spectrum (FAS) combination equation using SCr and CysC, based on Q_{SCr}(age)[S11]	FAS eGFRcr-cys	SCr, CysC, Age, Sex
Full Age Spectrum (FAS) combination equation using SCr and CysC, based on Q_{SCr}(height)[S11]	FAS-Height eGFRcr-cys	SCr, CysC, Age, Sex, Height
Mean of r-LMR eGFRcr and r-LMR eGFRcys equations[23]	r-LMR eGFRcr-cys	SCr, CysC, Age, Sex

*Equation was developed with cystatin C not calibrated to IFCC reference material. **Equation was originally developed for an adult population but has later been validated for pediatric patients. ***The equation was constructed by the authors and not presented in the reference articles.
BUN, blood urea nitrogen; CysC, cystatin C; eGFR, estimated GFR; SCr, plasma/serum creatinine

Supplementary Table S2. Performance of selected GFR estimating equations divided by the used analyzer

	Equation	Median bias (95% CI), ml/min/1.73m²	IQR (95% CI), ml/min/1.73m²	P_{10} (95% CI), %	P_{30} (95% CI), %
Creatinine- based equations	**CKiD (2009) eGFRcr**	17.2 (14.4 to 20.0)<br>26.1 (16.2 to 33.7)	25.8 (23.3 to 30.8)<br>39.0 (33.2 to 45.5)	21.1 (17.2 to 25.3)<br>11.7 (6.6 to 17.5)	55.4 (50.4 to 60.4)<br>42.3 (34.3 to 50.4)
	**CKiD U25 eGFRcr**	10.4 (9.4 to 13.1)<br>16.5 (11.3 to 21.7)	24.1 (20.2 to 27.1)<br>25.6 (22.5 to 30.7)	28.8 (24.3 to 33.2)<br>25.5 (18.2 to 32.8)	69.9 (65.2 to 74.4)<br>64.2 (56.2 to 72.3)
	**EKFC eGFRcr**	13.1 (10.3 to 14.6)<br>15.0 (12.1 to 17.4)	21.7 (18.8 to 24.4)<br>22.0 (18.4 to 25.8)	27.4 (23.0 to 31.9)<br>24.8 (17.5 to 32.1)	68.9 (64.1 to 73.4)<br>70.1 (62.0 to 77.4)
	**EKFC-Height eGFRcr**	9.9 (8.3 to 12.5)<br>11.2 (9.8 to 15.7)	19.4 (16.9 to 22.5)<br>22.0 (18.0 to 26.9)	30.3 (25.9 to 35.1)<br>32.8 (24.8 to 40.9)	74.9 (70.4 to 79.2)<br>74.5 (67.2 to 81.8)
	**r-LMR eGFRcr**	10.2 (8.3 to 11.8)<br>10.9 (8.3 to 15.6)	20.9 (18.1 to 23.5)<br>20.6 (16.3 to 24.3)	31.9 (27.2 to 36.7)<br>31.4 (24.1 to 39.4)	72.6 (68.1 to 77.0)<br>72.3 (65.0 to 79.6)
Cystatin C-based equations	**CKiD (2012) eGFRcys**	-9.7 (-12.0 to -8.2)<br>-25.1 (-28.8 to -22.4)	21.7 (19.1 to 24.7)<br>19.9 (16.1 to 26.1)	29.0 (24.5 to 33.5)<br>8.8 (4.4 to 13.9)	76.0 (71.5 to 80.2)<br>55.5 (47.4 to 63.5)
	**CKiD U25 eGFRcys**	-4.7 (-6.2 to -2.6)<br>-18.9 (-21.2 to -16.1)	20.8 (18.5 to 23.8)<br>19.2 (15.6 to 24.6)	31.9 (27.2 to 36.7)<br>17.5 (11.7 to 24.1)	82.8 (78.9 to 86.5)<br>72.3 (65.0 to 79.6)
	**EKFC eGFRcys**	0.2 (-1.4 to 2.7)<br>-12.5 (-16.1 to -9.8)	21.9 (19.1 to 25.3)<br>20.2 (16.3 to 24.4)	32.5 (27.7 to 37.2)<br>28.5 (21.2 to 36.5)	81.8 (77.8 to 85.5)<br>75.2 (67.9 to 82.5)
	**r-LMR eGFRcys**	-1.5 (-3.6 to -0.1)<br>-14.4 (-16.7 to -11.0)	21.0 (18.4 to 23.9)<br>20.1 (16.0 to 24.9)	35.6 (30.9 to 40.4)<br>24.1 (16.8 to 31.4)	83.1 (79.2 to 86.8)<br>76.6 (69.3 to 83.2)
Creatinine-Cystatin C-based combination equations	**CKiD (2012) eGFRcr-cys**	1.7 (0.3 to 2.6)<br>-3.6 (-7.4 to -2.2)	14.5 (12.3 to 17.2)<br>15.7 (11.0 to 19.0)	45.9 (40.9 to 50.9)<br>51.1 (43.1 to 59.1)	93.9 (91.6 to 96.3)<br>96.4 (92.7 to 99.3)
	**CKiD U25 eGFRcr-cys**	4.1 (3.0 to 5.2)<br>-1.1 (-2.4 to 1.1)	17.2 (14.2 to 18.8)<br>12.8 (10.3 to 17.2)	42.5 (37.5 to 47.5)<br>54.0 (46.0 to 62.0)	88.4 (85.0 to 91.6)<br>94.9 (91.2 to 98.5)
	**EKFC eGFRcr-cys**	6.2 (4.1 to 8.3)<br>0.1 (-1.7 to 2.6)	18.0 (16.4 to 20.3)<br>15.8 (12.3 to 18.2)	38.8 (34.0 to 43.8)<br>54.0 (46.0 to 62.0)	83.1 (79.2 to 86.8)<br>95.6 (92.0 to 98.5)
	**EKFC-Height eGFRcr-cys**	4.7 (3.5 to 6.4)<br>0.6 (-3.7 to 1.9)	17.5 (15.2 to 20.1)<br>15.5 (11.1 to 17.1)	40.4 (35.4 to 45.4)<br>53.3 (45.3 to 61.3)	84.4 (80.7 to 87.9)<br>94.9 (91.2 to 98.5)
	**r-LMR eGFRcr-cys**	4.4 (3.0 to 5.5)<br>-1.5 (-3.0 to 0.6)	17.5 (15.5 to 18.8)<br>13.6 (10.2 to 18.6)	41.7 (36.7 to 46.7)<br>52.6 (44.5 to 60.6)	86.0 (82.3 to 89.4)<br>96.4 (92.7 to 99.3)

Results are presented in different colors depending on the analyzer used for laboratory values. Results using Abbott Architect (n = 379) are presented in red (above) and results using Siemens Atellica Solution (n = 137) are presented in blue (below). Biases are presented as medians of estimated GFR (eGFR) – measured GFR (mGFR). Similarly, interquartile ranges (IQR) are for the differences of eGFR and mGFR. The P_{10} and P_{30} values are the percentage of eGFR values within 10% and 30%, respectively, of the mGFR values. The 95% confidence intervals for the given statistical values were calculated using 10^7 resamples and are presented in parentheses. Abbreviations of the equations are explained in Supplementary Table S1 and their mathematical expressions in Supplementary Item S1. CI, confidence interval; eGFR, estimated GFR

Supplementary Table S3. Accuracy of selected GFR estimating combination equations in different age groups and mGFR categories divided by the used analyzer

		P_{10} (95% CI), %					P_{30} (95% CI), %				
		Age group (years):					Age group (years):				
	Equation	<2<br>(n = 42)<br>(n = 30)	2 to <5<br>(n = 58)<br>(n = 33)	5 to <10<br>(n = 91)<br>(n = 17)	10 to <16<br>(n = 132)<br>(n = 39)	16 to <20<br>(n = 56)<br>(n = 18)	<2<br>(n = 42)<br>(n = 30)	2 to <5<br>(n = 58)<br>(n = 33)	5 to <10<br>(n = 91)<br>(n = 17)	10 to <16<br>(n = 132)<br>(n = 39)	16 to <20<br>(n = 56)<br>(n = 18)
Creatinine-Cystatin C-based combination equations	CKiD (2012) eGFRcr-cys	33.3 (19.0 to 47.6)<br>46.7 (30.0 to 63.3)	43.1 (31.0 to 55.2)<br>51.5 (33.3 to 69.7)	49.5 (39.6 to 59.3)<br>70.6 (47.1 to 88.2)	49.2 (40.9 to 57.6)<br>48.7 (33.3 to 64.1)	44.6 (32.1 to 57.1)<br>44.4 (22.2 to 66.7)	85.7 (73.8 to 95.2)<br>100.0 (100.0 to 100.0)	91.4 (82.8 to 98.3)<br>93.9 (84.8 to 100.0)	95.6 (91.2 to 98.9)<br>100.0 (100.0 to 100.0)	96.2 (92.4 to 99.2)<br>100.0 (100.0 to 100.0)	94.6 (87.5 to 100.0)<br>83.3 (66.7 to 100.0)
	CKiD U25 eGFRcr-cys	21.4 (9.5 to 33.3)<br>66.7 (50.0 to 83.3)	34.5 (22.4 to 46.6)<br>42.4 (27.3 to 60.6)	45.1 (35.2 to 54.9)<br>58.8 (35.3 to 82.4)	50.8 (42.4 to 59.1)<br>56.4 (41.0 to 71.8)	42.9 (30.4 to 55.4)<br>44.4 (22.2 to 66.7)	83.3 (71.4 to 92.9)<br>96.7 (90.0 to 100.0)	79.3 (69.0 to 89.7)<br>87.9 (75.8 to 97.0)	87.9 (81.3 to 94.5)<br>100.0 (100.0 to 100.0)	91.7 (86.4 to 96.2)<br>100.0 (100.0 to 100.0)	94.6 (87.5 to 100.0)<br>88.9 (72.2 to 100.0)
	EKFC eGFRcr-cys	31.0 (16.7 to 45.2)<br>60.0 (43.3 to 76.7)	31.0 (19.0 to 43.1)<br>45.5 (27.3 to 63.6)	51.6 (41.8 to 61.5)<br>64.7 (41.2 to 88.2)	41.7 (33.3 to 50.0)<br>59.0 (43.6 to 74.4)	25.0 (14.3 to 37.5)<br>38.9 (16.7 to 61.1)	64.3 (50.0 to 78.6)<br>96.7 (90.0 to 100.0)	72.4 (60.3 to 82.8)<br>90.9 (78.8 to 100.0)	89.0 (82.4 to 94.5)<br>100.0 (100.0 to 100.0)	89.4 (84.1 to 94.7)<br>100.0 (100.0 to 100.0)	83.9 (73.2 to 92.9)<br>88.9 (72.2 to 100.0)
	EKFC-Height eGFRcr-cys	23.8 (11.9 to 38.1)<br>66.7 (50.0 to 83.3)	31.0 (19.0 to 43.1)<br>48.5 (30.3 to 66.7)	51.6 (41.8 to 61.5)<br>70.6 (47.1 to 88.2)	45.5 (37.1 to 53.8)<br>46.2 (30.8 to 61.5)	32.1 (19.6 to 44.6)<br>38.9 (16.7 to 61.1)	71.4 (57.1 to 83.3)<br>100.0 (100.0 to 100.0)	74.1 (62.1 to 84.5)<br>90.9 (78.8 to 100.0)	85.7 (78.0 to 92.3)<br>94.1 (82.4 to 100.0)	90.9 (85.6 to 95.5)<br>97.4 (92.3 to 100.0)	87.5 (78.6 to 94.6)<br>88.9 (72.2 to 100.0)
	r-LMR eGFRcr-cys	28.6 (16.7 to 42.9)<br>53.3 (36.7 to 70.0)	36.2 (24.1 to 48.3)<br>45.5 (27.3 to 63.6)	49.5 (39.6 to 59.3)<br>64.7 (41.2 to 88.2)	45.5 (37.1 to 53.8)<br>59.0 (43.6 to 74.4)	35.7 (23.2 to 48.2)<br>38.9 (16.7 to 61.1)	69.0 (54.8 to 83.3)<br>96.7 (90.0 to 100.0)	81.0 (70.7 to 91.4)<br>93.9 (84.8 to 100.0)	87.9 (81.3 to 94.5)<br>100.0 (100.0 to 100.0)	90.2 (84.8 to 94.7)<br>100.0 (100.0 to 100.0)	91.1 (82.1 to 98.2)<br>88.9 (72.2 to 100.0)

		P_{10} (95% CI), %				P_{30} (95% CI), %			
		mGFR category (ml/min/1.73m²):				mGFR category (ml/min/1.73m²):			
	Equation	<30<br>(n = 13)<br>(n = 7)	30 to <60<br>(n = 83)<br>(n = 14)	60 to <90<br>(n = 184)<br>(n = 58)	≥90<br>(n = 99)<br>(n = 58)	<30<br>(n = 13)<br>(n = 7)	30 to <60<br>(n = 83)<br>(n = 14)	60 to <90<br>(n = 184)<br>(n = 58)	≥90<br>(n = 99)<br>(n = 58)
Creatinine-Cystatin C-based combination equations	CKiD (2012) eGFRcr-cys	23.1 (0.0 to 46.2)<br>71.4 (42.9 to 100.0)	48.2 (37.3 to 59.0)<br>64.3 (35.7 to 85.7)	45.7 (38.6 to 52.7)<br>50.0 (37.9 to 62.1)	47.5 (37.4 to 57.6)<br>46.6 (34.5 to 58.6)	61.5 (38.5 to 84.6)<br>100.0 (100.0 to 100.0)	88.0 (80.7 to 94.0)<br>92.9 (78.6 to 100.0)	96.2 (93.5 to 98.9)<br>96.6 (91.4 to 100.0)	99.0 (97.0 to 100.0)<br>96.6 (91.4 to 100.0)
	CKiD U25 eGFRcr-cys	30.8 (7.7 to 53.8)<br>42.9 (14.3 to 85.7)	43.4 (32.5 to 54.2)<br>64.3 (35.7 to 85.7)	38.0 (31.0 to 45.1)<br>50.0 (37.9 to 62.1)	51.5 (41.4 to 61.6)<br>56.9 (44.8 to 69.0)	61.5 (38.5 to 84.6)<br>100.0 (100.0 to 100.0)	84.3 (75.9 to 91.6)<br>92.9 (78.6 to 100.0)	88.6 (83.7 to 92.9)<br>94.8 (87.9 to 100.0)	94.9 (89.9 to 99.0)<br>94.8 (87.9 to 100.0)
	EKFC eGFRcr-cys	23.1 (0.0 to 46.2)<br>42.9 (14.3 to 85.7)	33.7 (24.1 to 44.6)<br>50.0 (21.4 to 78.6)	28.8 (22.3 to 35.3)<br>44.8 (32.8 to 56.9)	63.6 (54.5 to 72.7)<br>65.5 (53.4 to 77.6)	76.9 (53.8 to 100.0)<br>100.0 (100.0 to 100.0)	77.1 (67.5 to 85.5)<br>92.9 (78.6 to 100.0)	77.2 (71.2 to 83.2)<br>93.1 (86.2 to 98.3)	100.0 (100.0 to 100.0)<br>98.3 (94.8 to 100.0)
	EKFC-Height eGFRcr-cys	15.4 (0.0 to 38.5)<br>28.6 (0.0 to 57.1)	36.1 (26.5 to 47.0)<br>42.9 (21.4 to 71.4)	31.5 (25.0 to 38.6)<br>46.6 (34.5 to 58.6)	63.6 (54.5 to 72.7)<br>65.5 (53.4 to 77.6)	69.2 (46.2 to 92.3)<br>71.4 (42.9 to 100.0)	81.9 (73.5 to 90.4)<br>92.9 (78.6 to 100.0)	78.3 (72.3 to 84.2)<br>94.8 (87.9 to 100.0)	100.0 (100.0 to 100.0)<br>98.3 (94.8 to 100.0)
	r-LMR eGFRcr-cys	30.8 (7.7 to 53.8)<br>42.9 (14.3 to 85.7)	33.7 (24.1 to 44.6)<br>57.1 (28.6 to 78.6)	35.3 (28.3 to 42.4)<br>53.4 (41.4 to 65.5)	61.6 (51.5 to 70.7)<br>51.7 (39.7 to 63.8)	61.5 (38.5 to 84.6)<br>100.0 (100.0 to 100.0)	74.7 (65.1 to 83.1)<br>85.7 (64.3 to 100.0)	85.3 (79.9 to 90.2)<br>96.6 (91.4 to 100.0)	100.0 (100.0 to 100.0)<br>98.3 (94.8 to 100.0)

Results are presented in different colors depending on the analyzer used for laboratory values. Results using Abbott Architect are presented in red (above) and results using Siemens Atellica Solution are presented in blue (below). The P_{10} and P_{30} values are the percentage of estimated GFR values within 10% and 30%, respectively, of the measured GFR values. The 95% confidence intervals were calculated using 10^7 resamples and are presented in parentheses. Abbreviations of the equations are explained in Supplementary Table S1 and their mathematical expressions in Supplementary Item S1. CI, confidence interval; eGFR, estimated GFR; mGFR, measured GFR

Supplementary Table S4. Accuracy of selected GFR estimating combination equations in different diagnosis groups and by sex divided by the used analyzer

		P_{10} (95% CI), %				P_{30} (95% CI), %			
		Diagnosis group:				Diagnosis group:			
	Equation	T + MN<br>(n = 12)<br>(n = 2)	T (No MN)<br>(n = 193)<br>(n = 66)	MN (No T)<br>(n = 15)<br>(n = 3)	No T or MN<br>(n = 159)<br>(n = 66)	T + MN<br>(n = 12)<br>(n = 2)	T (No MN)<br>(n = 193)<br>(n = 66)	MN (No T)<br>(n = 15)<br>(n = 3)	No T or MN<br>(n = 159)<br>(n = 66)
Creatinine-Cystatin C-based combination equations	CKiD (2012) eGFRcr-cys	58.3 (33.3 to 83.3)<br>0.0 (0.0 to 0.0)	50.3 (43.0 to 57.5)<br>39.4 (27.3 to 51.5)	13.3 (0.0 to 33.3)<br>100.0 (100.0 to 100.0)	42.8 (35.2 to 50.3)<br>62.1 (50.0 to 74.2)	91.7 (75.0 to 100.0)<br>100.0 (100.0 to 100.0)	95.3 (92.2 to 97.9)<br>93.9 (87.9 to 98.5)	100.0 (100.0 to 100.0)<br>100.0 (100.0 to 100.0)	91.8 (87.4 to 95.6)<br>98.5 (95.5 to 100.0)
	CKiD U25 eGFRcr-cys	58.3 (33.3 to 83.3)<br>0.0 (0.0 to 0.0)	43.0 (36.3 to 50.3)<br>50.0 (37.9 to 62.1)	13.3 (0.0 to 33.3)<br>66.7 (0.0 to 100.0)	43.4 (35.8 to 50.9)<br>59.1 (47.0 to 71.2)	91.7 (75.0 to 100.0)<br>50.0 (0.0 to 100.0)	91.7 (87.6 to 95.3)<br>93.9 (87.9 to 98.5)	66.7 (40.0 to 86.7)<br>100.0 (100.0 to 100.0)	86.2 (80.5 to 91.2)<br>97.0 (92.4 to 100.0)
	EKFC eGFRcr-cys	50.0 (25.0 to 75.0)<br>0.0 (0.0 to 0.0)	42.0 (35.2 to 48.7)<br>56.1 (43.9 to 68.2)	6.7 (0.0 to 20.0)<br>33.3 (0.0 to 100.0)	37.1 (29.6 to 44.7)<br>54.5 (42.4 to 66.7)	75.0 (50.0 to 100.0)<br>100.0 (100.0 to 100.0)	87.0 (82.4 to 91.7)<br>95.5 (89.4 to 100.0)	60.0 (33.3 to 86.7)<br>100.0 (100.0 to 100.0)	81.1 (74.8 to 86.8)<br>95.5 (89.4 to 100.0)
	EKFC-Height eGFRcr-cys	50.0 (25.0 to 75.0)<br>0.0 (0.0 to 0.0)	40.9 (34.2 to 47.7)<br>56.1 (43.9 to 68.2)	13.3 (0.0 to 33.3)<br>33.3 (0.0 to 100.0)	41.5 (34.0 to 49.1)<br>53.0 (40.9 to 65.2)	75.0 (50.0 to 100.0)<br>100.0 (100.0 to 100.0)	89.1 (84.5 to 93.3)<br>95.5 (89.4 to 100.0)	66.7 (40.0 to 86.7)<br>100.0 (100.0 to 100.0)	81.1 (74.8 to 86.8)<br>93.9 (87.9 to 98.5)
	r-LMR eGFRcr-cys	58.3 (33.3 to 83.3)<br>0.0 (0.0 to 0.0)	38.9 (32.1 to 45.6)<br>50.0 (37.9 to 62.1)	13.3 (0.0 to 33.3)<br>66.7 (0.0 to 100.0)	46.5 (39.0 to 54.1)<br>56.1 (43.9 to 68.2)	83.3 (58.3 to 100.0)<br>100.0 (100.0 to 100.0)	88.6 (83.9 to 92.7)<br>97.0 (92.4 to 100.0)	60.0 (33.3 to 86.7)<br>100.0 (100.0 to 100.0)	85.5 (79.9 to 90.6)<br>95.5 (89.4 to 100.0)

		P_{10} (95% CI), %		P_{30} (95% CI), %	
	Equation	Female<br>(n = 160)<br>(n = 59)	Male<br>(n = 219)<br>(n = 78)	Female<br>(n = 160)<br>(n = 59)	Male<br>(n = 219)<br>(n = 78)
Creatinine-Cystatin C-based combination equations	CKiD (2012) eGFRcr-cys	45.0 (37.5 to 52.5)<br>50.8 (37.3 to 62.7)	46.6 (40.2 to 53.0)<br>51.3 (39.7 to 62.8)	95.6 (92.5 to 98.8)<br>96.6 (91.5 to 100.0)	92.7 (89.0 to 95.9)<br>96.2 (91.0 to 100.0)
	CKiD U25 eGFRcr-cys	41.9 (34.4 to 49.4)<br>54.2 (40.7 to 66.1)	42.9 (36.5 to 49.3)<br>53.8 (42.3 to 65.4)	91.2 (86.9 to 95.6)<br>93.2 (86.4 to 98.3)	86.3 (81.7 to 90.9)<br>96.2 (91.0 to 100.0)
	EKFC eGFRcr-cys	34.4 (26.9 to 41.9)<br>52.5 (39.0 to 64.4)	42.0 (35.6 to 48.4)<br>55.1 (43.6 to 66.7)	80.6 (74.4 to 86.2)<br>94.9 (88.1 to 100.0)	84.9 (79.9 to 89.5)<br>96.2 (91.0 to 100.0)
	EKFC-Height eGFRcr-cys	37.5 (30.0 to 45.0)<br>54.2 (40.7 to 66.1)	42.5 (36.1 to 48.9)<br>52.6 (41.0 to 64.1)	82.5 (76.2 to 88.1)<br>94.9 (88.1 to 100.0)	85.8 (81.3 to 90.4)<br>94.9 (89.7 to 98.7)
	r-LMR eGFRcr-cys	39.4 (31.9 to 46.9)<br>54.2 (40.7 to 66.1)	43.4 (37.0 to 49.8)<br>51.3 (39.7 to 62.8)	86.9 (81.2 to 91.9)<br>98.3 (94.9 to 100.0)	85.4 (80.4 to 90.0)<br>94.9 (89.7 to 98.7)

Results are presented in different colors depending on the analyzer used for laboratory values. Results using Abbott Architect are presented in red (above) and results using Siemens Atellica Solution are presented in blue (below). The P_{10} and P_{30} values are the percentage of estimated GFR values within 10% and 30%, respectively, of the measured GFR values. The 95% confidence intervals were calculated using 10^7 resamples and are presented in parentheses. Abbreviations of the equations are explained in Supplementary Table S1 and their mathematical expressions in Supplementary Item S1. CI, confidence interval; eGFR, estimated GFR; mGFR, measured GFR

Supplementary Table S5. Performance of all analyzed GFR estimating equations in the entire cohort (N = 516)

	Equation	Median bias (95% CI), ml/min/1.73m²	IQR (95% CI), ml/min/1.73m²	P_{10} (95% CI), %	P_{30} (95% CI), %
Creatinine-based equations	CKD-EPI40 eGFRcr	9.8 (8.3 to 12.3)	23.0 (20.9 to 26.4)	30.2 (26.4 to 34.3)	70.7 (66.9 to 74.6)
	CKiD (2009) eGFRcr	18.5 (15.9 to 21.8)	31.1 (27.1 to 36.2)	18.6 (15.3 to 22.1)	51.9 (47.7 to 56.2)
	CKiD U25 eGFRcr	11.9 (10.1 to 14.9)	24.4 (21.7 to 27.2)	27.9 (24.0 to 31.8)	68.4 (64.3 to 72.5)
	EKFC eGFRcr	13.7 (11.9 to 15.1)	22.2 (19.6 to 24.2)	26.7 (23.1 to 30.6)	69.2 (65.1 to 73.1)
	EKFC-Height eGFRcr	10.6 (9.2 to 12.6)	20.1 (18.0 to 22.9)	31.0 (27.1 to 35.1)	74.8 (70.9 to 78.5)
	FAS eGFRcr	20.3 (17.8 to 22.8)	29.0 (25.3 to 31.9)	18.8 (15.5 to 22.3)	53.5 (49.2 to 57.8)
	FAS-Height eGFRcr	16.5 (13.9 to 18.8)	28.1 (24.2 to 31.1)	23.1 (19.6 to 26.7)	59.7 (55.4 to 64.0)
	FM eGFRcr	12.5 (10.7 to 14.6)	24.9 (21.8 to 27.3)	26.0 (22.3 to 29.8)	69.2 (65.1 to 73.1)
	GQ eGFRcr	15.9 (14.5 to 17.1)	19.9 (17.4 to 21.9)	20.7 (17.2 to 24.2)	65.3 (61.2 to 69.4)
	LMR18 eGFRcr	10.0 (9.0 to 12.0)	20.9 (18.9 to 22.9)	31.0 (27.1 to 35.1)	74.4 (70.5 to 78.1)
	r-LMR eGFRcr	10.4 (8.8 to 11.6)	20.9 (18.4 to 23.2)	31.8 (27.7 to 35.9)	72.5 (68.6 to 76.4)
	Schwartz-Lyon eGFRcr	10.5 (7.5 to 12.9)	26.7 (22.6 to 29.2)	30.2 (26.4 to 34.3)	68.6 (64.5 to 72.5)
Cystatin C-based equations	Berg eGFRcys	-0.8 (-2.9 to 1.0)	24.7 (21.5 to 27.3)	30.2 (26.4 to 34.3)	75.2 (71.5 to 78.9)
	CAPA eGFRcys	3.6 (1.1 to 5.3)	24.7 (22.6 to 28.0)	28.3 (24.4 to 32.2)	71.7 (67.8 to 75.6)
	CKD-EPI (2012) eGFRcys	-1.5 (-3.7 to 0.2)	25.1 (22.2 to 27.9)	29.7 (25.8 to 33.7)	73.6 (69.8 to 77.3)
	CKiD (2012) eGFRcys	-14.3 (-17.0 to -11.6)	23.6 (21.8 to 26.0)	23.6 (20.0 to 27.3)	70.5 (66.7 to 74.4)
	CKiD U25 eGFRcys	-8.6 (-10.5 to -6.2)	23.1 (21.1 to 25.1)	28.1 (24.2 to 32.0)	80.0 (76.6 to 83.5)
	EKFC eGFRcys	-3.1 (-5.0 to -1.4)	23.2 (20.8 to 25.6)	31.4 (27.5 to 35.5)	80.0 (76.6 to 83.3)
	FAS eGFRcys	-0.6 (-2.9 to 1.1)	22.9 (20.6 to 25.4)	31.8 (27.7 to 35.9)	80.8 (77.3 to 84.1)
	r-LMR eGFRcys	-5.2 (-7.1 to -3.6)	22.0 (19.8 to 23.8)	32.6 (28.5 to 36.6)	81.4 (77.9 to 84.7)
Creatinine-Cystatin C-based combination equations	CKiD (2012) eGFRcr-cys	0.1 (-1.1 to 1.0)	14.6 (13.2 to 16.3)	47.3 (43.0 to 51.6)	94.6 (92.6 to 96.5)
	CKiD U25 eGFRcr-cys	2.8 (1.5 to 3.9)	15.7 (13.9 to 18.3)	45.5 (41.3 to 49.8)	90.1 (87.4 to 92.6)
	EKFC eGFRcr-cys	4.0 (3.0 to 5.9)	17.6 (15.6 to 19.4)	42.8 (38.6 to 47.1)	86.4 (83.3 to 89.3)
	EKFC-Height eGFRcr-cys	3.2 (2.1 to 4.0)	17.1 (15.3 to 19.2)	43.8 (39.5 to 48.1)	87.2 (84.3 to 89.9)
	FAS eGFRcr-cys	7.0 (5.5 to 8.6)	18.3 (16.5 to 20.3)	35.7 (31.6 to 39.7)	81.4 (77.9 to 84.7)
	FAS-Height eGFRcr-cys	6.0 (4.7 to 7.0)	17.9 (15.4 to 20.3)	38.6 (34.3 to 42.8)	82.6 (79.3 to 85.9)
	r-LMR eGFRcr-cys	2.6 (1.0 to 3.7)	16.9 (15.1 to 18.8)	44.6 (40.3 to 48.8)	88.8 (86.0 to 91.5)

Biases are presented as medians of estimated GFR (eGFR) – measured GFR (mGFR). Similarly, interquartile ranges (IQR) are for the differences of eGFR and mGFR. The P_{10} and P_{30} values are the percentage of eGFR values within 10% and 30%, respectively, of the mGFR values. The 95% confidence intervals for the given statistical values were calculated using 10^7 resamples and are presented in parentheses. Abbreviations of the equations are explained in Supplementary Table S1 and their mathematical expressions in Supplementary Item S1. CI, confidence interval; eGFR, estimated GFR

Supplementary Table S6. Accuracy of all analyzed GFR estimating equations in different age groups

		P_{10} (95% CI), %					P_{30} (95% CI), %				
		Age group (years):					Age group (years):				
	Equation	<2 (n = 72)	2 to <5 (n = 91)	5 to <10 (n = 108)	10 to <16 (n = 171)	16 to < 20 (n = 74)	<2 (n = 72)	2 to <5 (n = 91)	5 to <10 (n = 108)	10 to <16 (n = 171)	16 to < 20 (n = 74)
Creatinine-based equations	CKD-EPI40 eGFRcr	12.5 (5.6 to 20.8)	23.1 (14.3 to 31.9)	38.9 (29.6 to 48.1)	38.0 (31.0 to 45.6)	25.7 (16.2 to 36.5)	45.8 (34.7 to 56.9)	67.0 (57.1 to 76.9)	80.6 (73.1 to 88.0)	74.3 (67.8 to 80.7)	77.0 (67.6 to 86.5)
	CKiD (2009) eGFRcr	9.7 (4.2 to 16.7)	4.4 (1.1 to 8.8)	15.7 (9.3 to 23.1)	26.9 (20.5 to 33.9)	29.7 (20.3 to 40.5)	26.4 (16.7 to 37.5)	26.4 (17.6 to 35.2)	45.4 (36.1 to 54.6)	68.4 (61.4 to 75.4)	79.7 (70.3 to 87.8)
	CKiD U25 eGFRcr	16.7 (8.3 to 25.0)	23.1 (14.3 to 31.9)	31.5 (23.1 to 40.7)	35.7 (28.7 to 42.7)	21.6 (12.2 to 31.1)	55.6 (44.4 to 66.7)	54.9 (45.1 to 64.8)	68.5 (59.3 to 76.9)	77.8 (71.3 to 83.6)	75.7 (66.2 to 85.1)
	EKFC eGFRcr	9.7 (4.2 to 16.7)	18.7 (11.0 to 27.5)	37.0 (27.8 to 46.3)	32.7 (25.7 to 39.8)	24.3 (14.9 to 33.8)	44.4 (33.3 to 55.6)	61.5 (51.6 to 71.4)	81.5 (74.1 to 88.9)	73.7 (67.3 to 80.1)	74.3 (63.5 to 83.8)
	EKFC-Height eGFRcr	16.7 (8.3 to 25.0)	24.2 (15.4 to 33.0)	34.3 (25.9 to 43.5)	39.2 (32.2 to 46.8)	29.7 (20.3 to 40.5)	58.3 (47.2 to 69.4)	63.7 (53.8 to 73.6)	82.4 (75.0 to 88.9)	79.5 (73.1 to 85.4)	82.4 (73.0 to 90.5)
	FAS eGFRcr	9.7 (4.2 to 16.7)	9.9 (4.4 to 16.5)	27.8 (19.4 to 36.1)	22.2 (16.4 to 28.7)	17.6 (9.5 to 27.0)	36.1 (25.0 to 47.2)	40.7 (30.8 to 50.5)	63.0 (53.7 to 72.2)	61.4 (53.8 to 68.4)	54.1 (43.2 to 64.9)
	FAS-Height eGFRcr	8.3 (2.8 to 15.3)	20.9 (13.2 to 29.7)	25.9 (17.6 to 34.3)	27.5 (21.1 to 34.5)	25.7 (16.2 to 36.5)	33.3 (22.2 to 44.4)	48.4 (38.5 to 58.2)	62.0 (52.8 to 71.3)	71.9 (64.9 to 78.4)	67.6 (56.8 to 78.4)
	FM eGFRcr	18.1 (9.7 to 27.8)	27.5 (18.7 to 37.4)	25.9 (17.6 to 34.3)	27.5 (21.1 to 34.5)	28.4 (18.9 to 39.2)	73.6 (62.5 to 83.3)	59.3 (49.5 to 69.2)	63.0 (53.7 to 72.2)	73.1 (66.1 to 79.5)	77.0 (67.6 to 86.5)
	GQ eGFRcr	11.1 (4.2 to 19.4)	5.5 (1.1 to 11.0)	21.3 (13.9 to 29.6)	30.4 (23.4 to 37.4)	25.7 (16.2 to 36.5)	50.0 (38.9 to 61.1)	42.9 (33.0 to 52.7)	71.3 (63.0 to 79.6)	73.1 (66.1 to 79.5)	81.1 (71.6 to 89.2)
	LMR18 eGFRcr	6.9 (1.4 to 13.9)	28.6 (19.8 to 38.5)	40.7 (31.5 to 50.0)	38.6 (31.6 to 46.2)	25.7 (16.2 to 36.5)	48.6 (37.5 to 59.7)	68.1 (58.2 to 78.0)	84.3 (76.9 to 90.7)	78.9 (72.5 to 84.8)	82.4 (73.0 to 90.5)
	r-LMR eGFRcr	6.9 (1.4 to 13.9)	26.4 (17.6 to 35.2)	41.7 (32.4 to 50.9)	39.8 (32.7 to 47.4)	29.7 (20.3 to 40.5)	44.4 (33.3 to 55.6)	67.0 (57.1 to 76.9)	82.4 (75.0 to 88.9)	77.2 (70.8 to 83.0)	81.1 (71.6 to 89.2)
	Schwartz-Lyon eGFRcr	16.7 (8.3 to 25.0)	19.8 (12.1 to 28.6)	31.5 (23.1 to 40.7)	35.7 (28.7 to 42.7)	41.9 (31.1 to 52.7)	37.5 (26.4 to 48.6)	49.5 (39.6 to 59.3)	71.3 (63.0 to 79.6)	83.0 (77.2 to 88.3)	85.1 (77.0 to 93.2)
Cystatin C-based equations	Berg eGFRcys	19.4 (11.1 to 29.2)	33.0 (23.1 to 42.9)	36.1 (26.9 to 45.4)	30.4 (23.4 to 37.4)	28.4 (18.9 to 39.2)	68.1 (56.9 to 79.2)	74.7 (65.9 to 83.5)	75.0 (66.7 to 83.3)	77.8 (71.3 to 83.6)	77.0 (67.6 to 86.5)
	CAPA eGFRcys	31.9 (20.8 to 43.1)	19.8 (12.1 to 28.6)	29.6 (21.3 to 38.0)	31.6 (24.6 to 38.6)	25.7 (16.2 to 36.5)	68.1 (56.9 to 79.2)	59.3 (49.5 to 69.2)	70.4 (62.0 to 78.7)	76.6 (70.2 to 83.0)	81.1 (71.6 to 89.2)
	CKD-EPI (2012) eGFRcys	20.8 (12.5 to 30.6)	31.9 (22.0 to 41.8)	32.4 (24.1 to 41.7)	29.2 (22.8 to 36.3)	32.4 (21.6 to 43.2)	65.3 (54.2 to 76.4)	71.4 (61.5 to 80.2)	74.1 (65.7 to 82.4)	75.4 (69.0 to 81.9)	79.7 (70.3 to 87.8)
	CKiD (2012) eGFRcys	13.9 (6.9 to 22.2)	28.6 (19.8 to 38.5)	32.4 (24.1 to 41.7)	18.1 (12.3 to 24.0)	27.0 (17.6 to 37.8)	50.0 (38.9 to 61.1)	79.1 (70.3 to 86.8)	78.7 (70.4 to 86.1)	67.8 (60.8 to 74.9)	74.3 (63.5 to 83.8)
	CKiD U25 eGFRcys	9.7 (4.2 to 16.7)	34.1 (24.2 to 44.0)	31.5 (23.1 to 40.7)	32.7 (25.7 to 39.8)	23.0 (13.5 to 32.4)	58.3 (47.2 to 69.4)	84.6 (76.9 to 91.2)	88.0 (81.5 to 93.5)	80.7 (74.9 to 86.5)	82.4 (73.0 to 90.5)
	EKFC eGFRcys	18.1 (9.7 to 27.8)	35.2 (25.3 to 45.1)	37.0 (27.8 to 46.3)	33.3 (26.3 to 40.4)	27.0 (17.6 to 37.8)	66.7 (55.6 to 77.8)	76.9 (68.1 to 85.7)	86.1 (79.6 to 92.6)	83.0 (77.2 to 88.3)	81.1 (71.6 to 89.2)
	FAS eGFRcys	22.2 (12.5 to 31.9)	35.2 (25.3 to 45.1)	34.3 (25.9 to 43.5)	32.7 (25.7 to 39.8)	31.1 (20.3 to 41.9)	75.0 (65.3 to 84.7)	75.8 (67.0 to 84.6)	84.3 (76.9 to 90.7)	81.9 (76.0 to 87.7)	85.1 (77.0 to 93.2)
	r-LMR eGFRcys	15.3 (6.9 to 23.6)	36.3 (26.4 to 46.2)	37.0 (27.8 to 46.3)	33.3 (26.3 to 40.4)	36.5 (25.7 to 47.3)	65.3 (54.2 to 76.4)	81.3 (72.5 to 89.0)	87.0 (80.6 to 92.6)	83.0 (77.2 to 88.3)	85.1 (77.0 to 93.2)
Creatinine-Cystatin C-based combination equations	CKiD (2012) eGFRcr-cys	38.9 (27.8 to 50.0)	46.2 (36.3 to 56.0)	52.8 (43.5 to 62.0)	49.1 (41.5 to 56.7)	44.6 (33.8 to 55.4)	91.7 (84.7 to 97.2)	92.3 (86.8 to 97.8)	96.3 (92.6 to 99.1)	97.1 (94.2 to 99.4)	91.9 (85.1 to 97.3)
	CKiD U25 eGFRcr-cys	40.3 (29.2 to 51.4)	37.4 (27.5 to 47.3)	47.2 (38.0 to 56.5)	52.0 (44.4 to 59.6)	43.2 (32.4 to 54.1)	88.9 (80.6 to 95.8)	82.4 (74.7 to 90.1)	89.8 (84.3 to 95.4)	93.6 (89.5 to 97.1)	93.2 (86.5 to 98.6)
	EKFC eGFRcr-cys	43.1 (31.9 to 54.2)	36.3 (26.4 to 46.2)	53.7 (44.4 to 63.0)	45.6 (38.0 to 53.2)	28.4 (18.9 to 39.2)	77.8 (68.1 to 87.5)	79.1 (70.3 to 86.8)	90.7 (85.2 to 95.4)	91.8 (87.7 to 95.9)	85.1 (77.0 to 93.2)
	EKFC-Height eGFRcr-cys	41.7 (30.6 to 52.8)	37.4 (27.5 to 47.3)	54.6 (45.4 to 63.9)	45.6 (38.0 to 53.2)	33.8 (23.0 to 44.6)	83.3 (75.0 to 91.7)	80.2 (71.4 to 87.9)	87.0 (80.6 to 92.6)	92.4 (88.3 to 95.9)	87.8 (79.7 to 94.6)
	FAS eGFRcr-cys	37.5 (26.4 to 48.6)	24.2 (15.4 to 33.0)	40.7 (31.5 to 50.0)	41.5 (33.9 to 49.1)	27.0 (17.6 to 37.8)	81.9 (72.2 to 90.3)	70.3 (60.4 to 79.1)	83.3 (75.9 to 89.8)	86.0 (80.7 to 91.2)	81.1 (71.6 to 89.2)
	FAS-Height eGFRcr-cys	37.5 (26.4 to 48.6)	30.8 (22.0 to 40.7)	38.9 (29.6 to 48.1)	44.4 (36.8 to 52.0)	35.1 (24.3 to 45.9)	80.6 (70.8 to 88.9)	74.7 (65.9 to 83.5)	82.4 (75.0 to 88.9)	87.7 (82.5 to 92.4)	82.4 (73.0 to 90.5)
	r-LMR eGFRcr-cys	38.9 (27.8 to 50.0)	39.6 (29.7 to 49.5)	51.9 (42.6 to 61.1)	48.5 (40.9 to 56.1)	36.5 (25.7 to 47.3)	80.6 (70.8 to 88.9)	85.7 (78.0 to 92.3)	89.8 (84.3 to 95.4)	92.4 (88.3 to 95.9)	90.5 (83.8 to 95.9)

The P_{10} and P_{30} values are the percentage of estimated GFR values within 10% and 30%, respectively, of the measured GFR values. The 95% confidence intervals were calculated using 10^7 resamples and are presented in parentheses. Abbreviations of the equations are explained in Supplementary Table S1 and their mathematical expressions in Supplementary Item S1. CI, confidence interval; eGFR, estimated GFR

Supplementary Table S7. Accuracy of all analyzed GFR estimating equations in different mGFR categories

		P_{10} (95% CI), %				P_{30} (95% CI), %			
	Equation	mGFR category (ml/min/1.73m²): <30 (n = 20)	30 to <60 (n = 97)	60 to <90 (n = 242)	≥90 (n = 157)	mGFR category (ml/min/1.73m²): <30 (n = 20)	30 to <60 (n = 97)	60 to <90 (n = 242)	≥90 (n = 157)
Creatinine-based equations	CKD-EPI40 eGFRcr	20.0 (5.0 to 40.0)	27.8 (19.6 to 37.1)	24.0 (18.6 to 29.3)	42.7 (35.0 to 50.3)	50.0 (30.0 to 70.0)	68.0 (58.8 to 77.3)	58.7 (52.5 to 64.9)	93.6 (89.8 to 97.5)
	CKiD (2009) eGFRcr	25.0 (10.0 to 45.0)	15.5 (8.2 to 22.7)	16.5 (12.0 to 21.5)	22.9 (16.6 to 29.9)	65.0 (45.0 to 85.0)	55.7 (45.4 to 66.0)	49.2 (43.0 to 55.4)	52.2 (44.6 to 59.9)
	CKiD U25 eGFRcr	35.0 (15.0 to 55.0)	24.7 (16.5 to 34.0)	28.5 (22.7 to 34.3)	28.0 (21.0 to 35.0)	65.0 (45.0 to 85.0)	71.1 (61.9 to 80.4)	62.8 (56.6 to 69.0)	75.8 (68.8 to 82.2)
	EKFC eGFRcr	20.0 (5.0 to 40.0)	19.6 (12.4 to 27.8)	20.7 (15.7 to 26.0)	41.4 (33.8 to 49.0)	65.0 (45.0 to 85.0)	60.8 (51.5 to 70.1)	55.0 (48.8 to 61.2)	96.8 (93.6 to 99.4)
	EKFC-Height eGFRcr	15.0 (0.0 to 30.0)	28.9 (19.6 to 38.1)	24.0 (18.6 to 29.3)	45.2 (37.6 to 52.9)	60.0 (40.0 to 80.0)	74.2 (64.9 to 82.5)	62.0 (55.8 to 68.2)	96.8 (93.6 to 99.4)
	FAS eGFRcr	20.0 (5.0 to 40.0)	20.6 (13.4 to 28.9)	16.1 (11.6 to 21.1)	21.7 (15.3 to 28.0)	65.0 (45.0 to 85.0)	50.5 (40.2 to 60.8)	49.2 (43.0 to 55.4)	60.5 (52.9 to 68.2)
	FAS-Height eGFRcr	40.0 (20.0 to 60.0)	15.5 (8.2 to 22.7)	21.9 (16.9 to 27.3)	27.4 (20.4 to 34.4)	60.0 (40.0 to 80.0)	64.9 (55.7 to 74.2)	57.9 (51.7 to 64.0)	59.2 (51.6 to 66.9)
	FM eGFRcr	45.0 (25.0 to 65.0)	21.6 (13.4 to 29.9)	26.0 (20.7 to 31.8)	26.1 (19.1 to 33.1)	70.0 (50.0 to 90.0)	75.3 (66.0 to 83.5)	64.5 (58.3 to 70.2)	72.6 (65.6 to 79.6)
	GQ eGFRcr	15.0 (0.0 to 30.0)	11.3 (5.2 to 17.5)	13.2 (9.1 to 17.8)	38.9 (31.2 to 46.5)	40.0 (20.0 to 60.0)	49.5 (39.2 to 59.8)	54.1 (47.9 to 60.3)	95.5 (92.4 to 98.7)
	LMR18 eGFRcr	25.0 (10.0 to 45.0)	23.7 (15.5 to 32.0)	22.3 (17.4 to 27.7)	49.7 (42.0 to 57.3)	70.0 (50.0 to 90.0)	58.8 (48.5 to 68.0)	68.6 (62.8 to 74.4)	93.6 (89.8 to 97.5)
	r-LMR eGFRcr	25.0 (10.0 to 45.0)	24.7 (16.5 to 34.0)	22.7 (17.8 to 28.1)	51.0 (43.3 to 58.6)	70.0 (50.0 to 90.0)	53.6 (43.3 to 63.9)	67.4 (61.6 to 73.1)	92.4 (87.9 to 96.2)
	Schwartz-Lyon eGFRcr	30.0 (10.0 to 50.0)	30.9 (21.6 to 40.2)	31.0 (25.2 to 36.8)	28.7 (21.7 to 35.7)	60.0 (40.0 to 80.0)	73.2 (63.9 to 81.4)	65.3 (59.1 to 71.1)	72.0 (65.0 to 79.0)
Cystatin C-based equations	Berg eGFRcys	25.0 (10.0 to 45.0)	27.8 (19.6 to 37.1)	29.3 (23.6 to 35.1)	33.8 (26.8 to 41.4)	70.0 (50.0 to 90.0)	73.2 (63.9 to 81.4)	72.3 (66.5 to 77.7)	81.5 (75.2 to 87.3)
	CAPA eGFRcys	15.0 (0.0 to 30.0)	26.8 (18.6 to 36.1)	24.8 (19.4 to 30.2)	36.3 (28.7 to 43.9)	50.0 (30.0 to 70.0)	69.1 (59.8 to 78.4)	67.4 (61.6 to 73.1)	82.8 (76.4 to 88.5)
	CKD-EPI (2012) eGFRcys	15.0 (0.0 to 30.0)	32.0 (22.7 to 41.2)	29.3 (23.6 to 35.1)	30.6 (23.6 to 37.6)	70.0 (50.0 to 90.0)	73.2 (63.9 to 81.4)	69.4 (63.6 to 75.2)	80.9 (74.5 to 86.6)
	CKiD (2012) eGFRcys	45.0 (25.0 to 65.0)	38.1 (28.9 to 47.4)	25.2 (19.8 to 30.6)	9.6 (5.1 to 14.6)	65.0 (45.0 to 85.0)	90.7 (84.5 to 95.9)	74.0 (68.2 to 79.3)	53.5 (45.9 to 61.1)
	CKiD U25 eGFRcys	55.0 (35.0 to 75.0)	34.0 (24.7 to 43.3)	31.4 (25.6 to 37.2)	15.9 (10.2 to 21.7)	65.0 (45.0 to 85.0)	86.6 (79.4 to 92.8)	81.4 (76.4 to 86.4)	75.8 (68.8 to 82.2)
	EKFC eGFRcys	40.0 (20.0 to 60.0)	30.9 (21.6 to 40.2)	30.6 (24.8 to 36.4)	31.8 (24.8 to 39.5)	70.0 (50.0 to 90.0)	78.4 (70.1 to 86.6)	78.1 (72.7 to 83.1)	85.4 (79.6 to 90.4)
	FAS eGFRcys	20.0 (5.0 to 40.0)	27.8 (19.6 to 37.1)	34.3 (28.5 to 40.5)	31.8 (24.8 to 39.5)	65.0 (45.0 to 85.0)	74.2 (64.9 to 82.5)	81.8 (76.9 to 86.4)	85.4 (79.6 to 90.4)
	r-LMR eGFRcys	45.0 (25.0 to 65.0)	30.9 (21.6 to 40.2)	35.5 (29.8 to 41.7)	27.4 (20.4 to 34.4)	75.0 (55.0 to 90.0)	74.2 (64.9 to 82.5)	82.6 (77.7 to 87.2)	84.7 (79.0 to 89.8)
Creatinine-Cystatin C-based combination equations	CKiD (2012) eGFRcr-cys	40.0 (20.0 to 60.0)	50.5 (40.2 to 60.8)	46.7 (40.5 to 52.9)	47.1 (39.5 to 54.8)	75.0 (55.0 to 90.0)	88.7 (82.5 to 94.8)	96.3 (93.8 to 98.3)	98.1 (95.5 to 100.0)
	CKiD U25 eGFRcr-cys	35.0 (15.0 to 55.0)	46.4 (36.1 to 56.7)	40.9 (34.7 to 47.1)	53.5 (45.9 to 61.1)	75.0 (55.0 to 90.0)	85.6 (78.4 to 91.8)	90.1 (86.4 to 93.8)	94.9 (91.1 to 98.1)
	EKFC eGFRcr-cys	30.0 (10.0 to 50.0)	36.1 (26.8 to 45.4)	32.6 (26.9 to 38.4)	64.3 (56.7 to 72.0)	85.0 (70.0 to 100.0)	79.4 (71.1 to 86.6)	81.0 (76.0 to 86.0)	99.4 (98.1 to 100.0)
	EKFC-Height eGFRcr-cys	20.0 (5.0 to 40.0)	37.1 (27.8 to 46.4)	35.1 (29.3 to 41.3)	64.3 (56.7 to 72.0)	70.0 (50.0 to 90.0)	83.5 (76.3 to 90.7)	82.2 (77.3 to 86.8)	99.4 (98.1 to 100.0)
	FAS eGFRcr-cys	30.0 (10.0 to 50.0)	27.8 (19.6 to 37.1)	29.8 (24.0 to 35.5)	50.3 (42.7 to 58.0)	65.0 (45.0 to 85.0)	71.1 (61.9 to 80.4)	78.5 (73.1 to 83.5)	94.3 (90.4 to 97.5)
	FAS-Height eGFRcr-cys	25.0 (10.0 to 45.0)	30.9 (21.6 to 40.2)	35.1 (29.3 to 41.3)	50.3 (42.7 to 58.0)	65.0 (45.0 to 85.0)	76.3 (68.0 to 84.5)	79.3 (74.0 to 84.3)	93.6 (89.8 to 97.5)
	r-LMR eGFRcr-cys	35.0 (15.0 to 55.0)	37.1 (27.8 to 46.4)	39.7 (33.5 to 45.9)	58.0 (50.3 to 65.6)	75.0 (55.0 to 90.0)	76.3 (68.0 to 84.5)	88.0 (83.9 to 92.1)	99.4 (98.1 to 100.0)

The P_{10} and P_{30} values are the percentage of estimated GFR values within 10% and 30%, respectively, of the measured GFR values. The 95% confidence intervals were calculated using 10^7 resamples and are presented in parentheses. Abbreviations of the equations are explained in Supplementary Table S1 and their mathematical expressions in Supplementary Item S1. CI, confidence interval; eGFR, estimated GFR; mGFR, measured GFR

Supplementary Table S8. Demographics of the final cohort in patients with different transplant and malignant neoplasm diagnoses

Characteristic	Transplant and malignant neoplasm diagnosis (n = 14)	Transplant (no malignant neoplasm diagnosis) (n = 259)	Malignant neoplasm diagnosis (no transplant) (n = 18)	No transplant or malignant neoplasm diagnosis (n = 225)
**Female**	7 (50%)	125 (48%)	8 (44%)	79 (35%)
**Age, years**	10.3 (8.1–14.8)	8.7 (2.6–14.0)	5.1 (3.0–11.0)	10.7 (4.4–14.3)
**Weight, kg**	33.2 (24.0–54.3)	24.5 (12.4–42.9)	18.1 (13.3–29.1)	34.2 (17.0–53.4)
**Height, cm**	138 (124–160)	125 (87–151)	108 (96–136)	140 (103–163)
**BMI, kg/m²**	16.9 (15.4–21.5)	17.2 (15.9–19.2)	16.0 (14.8–17.7)	17.3 (15.7–20.6)
**P-Creatinine, µmol/l**	54.5 (50.5–67.2)	48.0 (32.0–70.0)	37.0 (29.2–64.0)	50.0 (38.0–70.0)
**P-Cystatin C, mg/l**	1.2 (1.1–1.6)	1.3 (1.1–1.6)	1.1 (0.9–1.4)	1.1 (0.9–1.3)
**P-Urea, mmol/l**	6.3 (5.3–7.8)	6.8 (5.1–9.4)	5.7 (4.5–7.1)	5.6 (4.6–7.5)
**mGFR, ml/min/1.73m²**	69 (61–78)	73 (59–90)	72 (65–84)	83 (68–96)
**mGFR <30**	0 (0%)	2 (1%)	0 (0%)	18 (8%)
**mGFR: 30 to <60**	3 (21%)	65 (25%)	4 (22%)	25 (11%)
**mGFR: 60 to <90**	10 (71%)	125 (48%)	13 (72%)	94 (42%)
**mGFR ≥90**	1 (7%)	67 (26%)	1 (6%)	88 (39%)

Age, weight, height, body mass index (BMI), creatinine, cystatin C, urea and measured glomerular filtration rate (mGFR) are presented as medians with interquartile ranges in parentheses. Other characteristics are presented as the number of patients and the proportion of the patient group. Transplant and malignant neoplasm diagnoses refer to patients that have the respective diagnosis prior to the measuring of GFR. Transplants include kidney, liver, heart, lung and small bowel transplants. BMI, body mass index; mGFR, measured GFR

Supplementary Table S9. Accuracy of all analyzed GFR estimating equations in different diagnosis groups based on transplantations (T) and malignant neoplasm diagnoses (MN)

		P_{10} (95% CI), %				P_{30} (95% CI), %			
		Diagnosis group:				Diagnosis group:			
	Equation	T + MN (n = 14)	T (No MN) (n = 259)	MN (No T) (n = 18)	No T or MN (n = 225)	T + MN (n = 14)	T (No MN) (n = 259)	MN (No T) (n = 18)	No T or MN (n = 225)
Creatinine-based equations	CKD-EPI40 eGFRcr	35.7 (14.3 to 64.3)	29.0 (23.6 to 34.4)	22.2 (5.6 to 44.4)	32.0 (25.8 to 38.2)	64.3 (35.7 to 85.7)	67.6 (61.8 to 73.4)	66.7 (44.4 to 88.9)	75.1 (69.3 to 80.4)
	CKiD (2009) eGFRcr	14.3 (0.0 to 35.7)	18.5 (13.9 to 23.6)	11.1 (0.0 to 27.8)	19.6 (14.7 to 24.9)	71.4 (50.0 to 92.9)	48.6 (42.5 to 54.8)	22.2 (5.6 to 44.4)	56.9 (50.2 to 63.1)
	CKiD U25 eGFRcr	21.4 (0.0 to 42.9)	27.0 (21.6 to 32.4)	11.1 (0.0 to 27.8)	30.7 (24.9 to 36.9)	71.4 (50.0 to 92.9)	66.0 (60.2 to 71.8)	61.1 (38.9 to 83.3)	71.6 (65.8 to 77.3)
	EKFC eGFRcr	21.4 (0.0 to 42.9)	22.8 (17.8 to 27.8)	22.2 (5.6 to 44.4)	32.0 (25.8 to 38.2)	57.1 (28.6 to 78.6)	65.3 (59.5 to 71.0)	61.1 (38.9 to 83.3)	75.1 (69.3 to 80.4)
	EKFC-Height eGFRcr	35.7 (14.3 to 64.3)	29.3 (23.9 to 35.1)	11.1 (0.0 to 27.8)	34.2 (28.0 to 40.4)	78.6 (57.1 to 100.0)	74.9 (69.5 to 79.9)	66.7 (44.4 to 88.9)	75.1 (69.3 to 80.4)
	FAS eGFRcr	7.1 (0.0 to 21.4)	17.8 (13.1 to 22.4)	5.6 (0.0 to 16.7)	21.8 (16.4 to 27.1)	50.0 (21.4 to 78.6)	48.6 (42.5 to 54.8)	38.9 (16.7 to 61.1)	60.4 (54.2 to 66.7)
	FAS-Height eGFRcr	21.4 (0.0 to 42.9)	22.0 (17.0 to 27.0)	5.6 (0.0 to 16.7)	25.8 (20.0 to 31.6)	71.4 (50.0 to 92.9)	58.3 (52.1 to 64.1)	44.4 (22.2 to 66.7)	61.8 (55.6 to 68.0)
	FM eGFRcr	28.6 (7.1 to 50.0)	23.9 (18.9 to 29.3)	16.7 (0.0 to 33.3)	28.9 (23.1 to 35.1)	64.3 (35.7 to 85.7)	68.7 (62.9 to 74.1)	55.6 (33.3 to 77.8)	71.1 (64.9 to 76.9)
	GQ eGFRcr	7.1 (0.0 to 21.4)	18.1 (13.5 to 22.8)	5.6 (0.0 to 16.7)	25.8 (20.0 to 31.6)	50.0 (21.4 to 78.6)	62.9 (57.1 to 68.7)	38.9 (16.7 to 61.1)	71.1 (64.9 to 76.9)
	LMR18 eGFRcr	14.3 (0.0 to 35.7)	27.0 (21.6 to 32.4)	22.2 (5.6 to 44.4)	37.3 (31.1 to 43.6)	57.1 (28.6 to 78.6)	69.1 (63.3 to 74.5)	66.7 (44.4 to 88.9)	82.2 (76.9 to 87.1)
	r-LMR eGFRcr	21.4 (0.0 to 42.9)	27.0 (21.6 to 32.4)	22.2 (5.6 to 44.4)	38.7 (32.4 to 44.9)	64.3 (35.7 to 85.7)	66.4 (60.6 to 72.2)	55.6 (33.3 to 77.8)	81.3 (76.0 to 86.2)
	Schwartz-Lyon eGFRcr	28.6 (7.1 to 50.0)	28.6 (23.2 to 34.0)	16.7 (0.0 to 33.3)	33.3 (27.1 to 39.6)	78.6 (57.1 to 100.0)	66.4 (60.6 to 72.2)	55.6 (33.3 to 77.8)	71.6 (65.8 to 77.3)
Cystatin C-based equations	Berg eGFRcys	42.9 (21.4 to 71.4)	28.6 (23.2 to 34.0)	11.1 (0.0 to 27.8)	32.9 (26.7 to 39.1)	85.7 (64.3 to 100.0)	76.8 (71.4 to 81.9)	38.9 (16.7 to 61.1)	75.6 (69.8 to 80.9)
	CAPA eGFRcys	28.6 (7.1 to 50.0)	30.9 (25.5 to 36.7)	0.0 (0.0 to 0.0)	27.6 (21.8 to 33.3)	78.6 (57.1 to 100.0)	78.8 (73.7 to 83.8)	33.3 (11.1 to 55.6)	66.2 (60.0 to 72.4)
	CKD-EPI (2012) eGFRcys	42.9 (21.4 to 71.4)	27.8 (22.4 to 33.2)	11.1 (0.0 to 27.8)	32.4 (26.2 to 38.7)	85.7 (64.3 to 100.0)	74.1 (68.7 to 79.5)	50.0 (27.8 to 72.2)	74.2 (68.4 to 80.0)
	CKiD (2012) eGFRcys	35.7 (14.3 to 64.3)	19.7 (15.1 to 24.7)	27.8 (11.1 to 50.0)	27.1 (21.3 to 32.9)	78.6 (57.1 to 100.0)	64.1 (58.3 to 69.9)	77.8 (55.6 to 94.4)	76.9 (71.1 to 82.2)
	CKiD U25 eGFRcys	21.4 (0.0 to 42.9)	29.7 (24.3 to 35.5)	11.1 (0.0 to 27.8)	28.0 (22.2 to 33.8)	100.0 (100.0 to 100.0)	72.6 (67.2 to 78.0)	72.2 (50.0 to 88.9)	88.0 (83.6 to 92.0)
	EKFC eGFRcys	35.7 (14.3 to 64.3)	29.3 (23.9 to 35.1)	22.2 (5.6 to 44.4)	34.2 (28.0 to 40.4)	92.9 (78.6 to 100.0)	78.0 (73.0 to 83.0)	61.1 (38.9 to 83.3)	83.1 (78.2 to 88.0)
	FAS eGFRcys	28.6 (7.1 to 50.0)	32.0 (26.3 to 37.8)	22.2 (5.6 to 44.4)	32.4 (26.2 to 38.7)	85.7 (64.3 to 100.0)	83.8 (79.2 to 88.0)	55.6 (33.3 to 77.8)	79.1 (73.8 to 84.4)
	r-LMR eGFRcys	42.9 (21.4 to 71.4)	30.5 (25.1 to 36.3)	16.7 (0.0 to 33.3)	35.6 (29.3 to 41.8)	100.0 (100.0 to 100.0)	77.2 (72.2 to 82.2)	61.1 (38.9 to 83.3)	86.7 (82.2 to 91.1)
Creatinine-Cystatin C-based combination equations	CKiD (2012) eGFRcr-cys	50.0 (21.4 to 78.6)	47.5 (41.3 to 53.7)	27.8 (11.1 to 50.0)	48.4 (41.8 to 55.1)	92.9 (78.6 to 100.0)	95.0 (92.3 to 97.3)	100.0 (100.0 to 100.0)	93.8 (90.7 to 96.9)
	CKiD U25 eGFRcr-cys	50.0 (21.4 to 78.6)	44.8 (38.6 to 51.0)	22.2 (5.6 to 44.4)	48.0 (41.3 to 54.7)	85.7 (64.3 to 100.0)	92.3 (88.8 to 95.4)	72.2 (50.0 to 88.9)	89.3 (85.3 to 93.3)
	EKFC eGFRcr-cys	42.9 (21.4 to 71.4)	45.6 (39.4 to 51.7)	11.1 (0.0 to 27.8)	42.2 (36.0 to 48.9)	78.6 (57.1 to 100.0)	89.2 (85.3 to 92.7)	66.7 (44.4 to 88.9)	85.3 (80.4 to 89.8)
	EKFC-Height eGFRcr-cys	42.9 (21.4 to 71.4)	44.8 (38.6 to 51.0)	16.7 (0.0 to 33.3)	44.9 (38.2 to 51.6)	78.6 (57.1 to 100.0)	90.7 (86.9 to 94.2)	72.2 (50.0 to 88.9)	84.9 (80.0 to 89.3)
	FAS eGFRcr-cys	35.7 (14.3 to 64.3)	38.6 (32.8 to 44.4)	11.1 (0.0 to 27.8)	34.2 (28.0 to 40.4)	64.3 (35.7 to 85.7)	86.9 (82.6 to 90.7)	50.0 (27.8 to 72.2)	78.7 (73.3 to 84.0)
	FAS-Height eGFRcr-cys	42.9 (21.4 to 71.4)	39.8 (34.0 to 45.9)	22.2 (5.6 to 44.4)	38.2 (32.0 to 44.4)	71.4 (50.0 to 92.9)	88.0 (83.8 to 91.9)	55.6 (33.3 to 77.8)	79.1 (73.8 to 84.4)
	r-LMR eGFRcr-cys	50.0 (21.4 to 78.6)	41.7 (35.9 to 47.9)	22.2 (5.6 to 44.4)	49.3 (42.7 to 56.0)	85.7 (64.3 to 100.0)	90.7 (87.3 to 94.2)	66.7 (44.4 to 88.9)	88.4 (84.0 to 92.4)

The P_{10} and P_{30} values are the percentage of estimated GFR values within 10% and 30%, respectively, of the measured GFR values. The 95% confidence intervals were calculated using 10^7 resamples and are presented in parentheses. Abbreviations of the equations are explained in Supplementary Table S1 and their mathematical expressions in Supplementary Item S1. CI, confidence interval; eGFR, estimated GFR

Supplementary Table S10. Demographics of the final cohort by sex

Characteristic	Female (n = 219)	Male (n = 297)
**Age, yr**	10.0 (3.3–14.5)	8.8 (3.4–13.9)
**Weight, kg**	30.1 (12.9–46.4)	27.0 (15.0–48.0)
**Height, cm**	134 (91–156)	128 (95–160)
**BMI, kg/m²**	17.4 (15.8–19.9)	17.1 (15.7–19.5)
**P-Creatinine, µmol/l**	48.0 (31.5–65.0)	50.0 (35.0–75.0)
**P-Cystatin C, mg/l**	1.2 (1.0–1.4)	1.2 (1.0–1.5)
**P-Urea, mmol/l**	5.6 (4.3–7.4)	6.6 (5.1–9.1)
**mGFR, ml/min/1.73m²**	79 (62–94)	76 (61–93)
**mGFR <30**	6 (3%)	14 (5%)
**mGFR: 30 to <60**	45 (21%)	52 (18%)
**mGFR: 60 to <90**	100 (46%)	142 (48%)
**mGFR ≥90**	68 (31%)	89 (30%)
**Transplant diagnosis**	132 (60%)	141 (47%)
**Malignant neoplasm diagnosis**	15 (7%)	17 (6%)

Age, weight, height, body mass index (BMI), creatinine, cystatin C, urea and measured glomerular filtration rate (mGFR) are presented as medians with interquartile ranges in parentheses. Other characteristics are presented as the number of patients and the proportion of the patient group. Transplant and malignant neoplasm diagnoses refer to patients that have the respective diagnosis prior to the measuring of GFR. Transplants include kidney, liver, heart, lung and small bowel transplants. BMI, body mass index; mGFR, measured GFR

Supplementary Table S11. Performance of all analyzed GFR estimating equations by sex

		Median bias (95% CI), ml/min/1.73m^2		IQR (95% CI), ml/min/1.73m^2		P10 (95% CI), %		P30 (95% CI), %	
	Equation	Female (n = 219)	Male (n = 297)	Female (n = 219)	Male (n = 297)	Female (n = 219)	Male (n = 297)	Female (n = 219)	Male (n = 297)
Creatinine-based equations	CKD-EPI40 eGFRcr	10.8 (8.3 to 13.8)	9.2 (6.5 to 12.3)	22.3 (18.2 to 25.5)	23.8 (20.9 to 29.0)	29.2 (23.3 to 35.2)	31.0 (25.9 to 36.4)	71.7 (65.8 to 77.6)	70.0 (64.6 to 75.1)
	CKiD (2009) eGFRcr	22.6 (18.3 to 26.1)	16.1 (13.6 to 19.4)	36.8 (30.1 to 42.3)	29.4 (25.0 to 33.0)	14.6 (10.0 to 19.6)	21.5 (16.8 to 26.3)	46.1 (39.7 to 52.5)	56.2 (50.5 to 62.0)
	CKiD U25 eGFRcr	10.9 (9.0 to 14.5)	12.8 (10.0 to 16.5)	25.5 (20.7 to 29.8)	24.1 (20.6 to 26.9)	25.6 (20.1 to 31.5)	29.6 (24.6 to 35.0)	70.3 (64.4 to 76.3)	67.0 (61.6 to 72.4)
	EKFC eGFRcr	15.1 (13.3 to 17.3)	12.4 (9.6 to 14.0)	20.9 (17.0 to 23.4)	22.5 (19.7 to 26.2)	23.7 (18.3 to 29.7)	29.0 (23.9 to 34.3)	66.7 (60.3 to 73.1)	71.0 (65.7 to 76.1)
	EKFC-Height eGFRcr	13.0 (10.0 to 15.2)	9.4 (6.7 to 11.2)	22.2 (17.4 to 26.1)	19.2 (17.0 to 21.9)	27.4 (21.5 to 33.3)	33.7 (28.3 to 39.1)	71.2 (65.3 to 77.2)	77.4 (72.7 to 82.2)
	FAS eGFRcr	24.1 (20.8 to 28.0)	17.6 (13.3 to 20.2)	27.9 (24.1 to 32.6)	27.5 (22.6 to 31.9)	14.2 (9.6 to 18.7)	22.2 (17.5 to 26.9)	44.7 (38.4 to 51.1)	59.9 (54.2 to 65.3)
	FAS-Height eGFRcr	18.6 (15.3 to 23.3)	14.8 (11.7 to 17.6)	33.3 (27.1 to 37.8)	23.6 (20.6 to 28.2)	21.5 (16.0 to 26.9)	24.2 (19.5 to 29.3)	54.8 (48.4 to 61.2)	63.3 (57.9 to 68.7)
	FM eGFRcr	15.1 (12.0 to 18.5)	9.6 (6.9 to 13.1)	23.6 (19.7 to 28.9)	23.0 (20.6 to 26.6)	19.2 (14.2 to 24.7)	31.0 (25.9 to 36.4)	63.9 (57.5 to 70.3)	73.1 (68.0 to 78.1)
	GQ eGFRcr	15.9 (13.6 to 17.6)	15.9 (14.4 to 17.7)	18.9 (15.1 to 22.3)	20.5 (17.4 to 22.6)	20.1 (15.1 to 25.6)	21.2 (16.5 to 25.9)	63.5 (57.1 to 69.9)	66.7 (61.3 to 72.1)
	LMR18 eGFRcr	11.5 (9.5 to 13.3)	9.3 (7.3 to 11.6)	19.5 (16.3 to 22.4)	22.1 (18.9 to 24.8)	28.3 (22.4 to 34.2)	33.0 (27.6 to 38.4)	75.3 (69.4 to 80.8)	73.7 (68.7 to 78.8)
	r-LMR eGFRcr	9.8 (7.8 to 11.6)	10.9 (8.8 to 13.3)	19.4 (16.2 to 22.2)	22.1 (19.2 to 25.1)	33.3 (27.4 to 39.7)	30.6 (25.6 to 36.0)	76.3 (70.3 to 81.7)	69.7 (64.3 to 74.7)
	Schwartz-Lyon eGFRcr	12.8 (9.3 to 15.9)	8.7 (6.3 to 12.6)	30.4 (26.1 to 35.3)	23.0 (19.8 to 26.9)	27.9 (21.9 to 33.8)	32.0 (26.6 to 37.4)	63.0 (56.6 to 69.4)	72.7 (67.7 to 77.8)
Cystatin C-based equations	Berg eGFRcys	1.1 (-0.8 to 4.0)	-2.9 (-5.0 to 0.0)	25.2 (20.6 to 29.1)	23.9 (20.0 to 27.3)	30.1 (24.2 to 36.1)	30.3 (25.3 to 35.7)	74.4 (68.5 to 79.9)	75.8 (70.7 to 80.5)
	CAPA eGFRcys	4.4 (1.2 to 7.7)	2.4 (-0.0 to 5.4)	24.3 (21.2 to 29.7)	24.6 (21.2 to 28.7)	29.7 (23.7 to 35.6)	27.3 (22.2 to 32.3)	73.5 (67.6 to 79.5)	70.4 (65.0 to 75.4)
	CKD-EPI (2012) eGFRcys	-2.9 (-4.5 to 0.5)	-0.9 (-3.8 to 1.2)	24.8 (20.4 to 28.9)	25.9 (21.8 to 29.5)	30.6 (24.7 to 37.0)	29.0 (23.9 to 34.3)	75.8 (69.9 to 81.3)	72.1 (67.0 to 77.1)
	CKiD (2012) eGFRcys	-12.1 (-17.0 to -9.5)	-15.1 (-18.5 to -12.4)	24.3 (20.7 to 27.7)	23.3 (20.4 to 26.5)	25.1 (19.6 to 31.1)	22.6 (17.8 to 27.3)	72.1 (66.2 to 78.1)	69.4 (64.0 to 74.4)
	CKiD U25 eGFRcys	-9.5 (-12.6 to -5.7)	-8.1 (-10.7 to -5.6)	23.2 (20.0 to 27.5)	22.5 (19.8 to 25.2)	28.8 (22.8 to 34.7)	27.6 (22.6 to 32.7)	77.6 (72.1 to 83.1)	81.8 (77.4 to 86.2)
	EKFC eGFRcys	-1.3 (-3.7 to 1.1)	-4.9 (-7.1 to -3.0)	22.7 (19.8 to 27.3)	22.6 (19.5 to 25.9)	32.4 (26.5 to 38.8)	30.6 (25.6 to 36.0)	80.8 (75.3 to 85.8)	79.5 (74.7 to 83.8)
	FAS eGFRcys	1.7 (-1.0 to 4.1)	-2.3 (-4.6 to -0.3)	23.0 (19.9 to 27.4)	21.5 (19.3 to 25.8)	31.1 (25.1 to 37.4)	32.3 (26.9 to 37.7)	80.4 (74.9 to 85.4)	81.1 (76.8 to 85.5)
	r-LMR eGFRcys	-5.7 (-8.7 to -3.2)	-5.0 (-7.3 to -3.1)	22.8 (19.4 to 26.4)	21.3 (18.6 to 24.4)	34.7 (28.3 to 41.1)	31.0 (25.9 to 36.4)	82.2 (77.2 to 87.2)	80.8 (76.1 to 85.2)
Creatinine-Cystatin C-based combination equations	CKiD (2012) eGFRcr-cys	-0.9 (-2.6 to 0.7)	0.3 (-0.6 to 1.9)	15.6 (12.8 to 19.4)	14.1 (12.4 to 16.1)	46.6 (40.2 to 53.0)	47.8 (42.1 to 53.5)	95.9 (93.2 to 98.2)	93.6 (90.6 to 96.3)
	CKiD U25 eGFRcr-cys	2.5 (0.4 to 4.0)	3.1 (1.4 to 4.4)	16.8 (13.4 to 20.4)	16.3 (13.2 to 18.4)	45.2 (38.8 to 51.6)	45.8 (40.1 to 51.5)	91.8 (88.1 to 95.4)	88.9 (85.2 to 92.3)
	EKFC eGFRcr-cys	6.2 (3.6 to 8.3)	3.2 (1.6 to 5.0)	17.5 (15.4 to 21.3)	16.6 (14.3 to 19.2)	39.3 (32.9 to 45.7)	45.5 (39.7 to 51.2)	84.5 (79.5 to 89.0)	87.9 (84.2 to 91.6)
	EKFC-Height eGFRcr-cys	3.8 (2.5 to 6.8)	2.8 (1.0 to 3.7)	18.7 (15.2 to 21.9)	15.1 (13.2 to 18.1)	42.0 (35.6 to 48.4)	45.1 (39.4 to 50.8)	85.8 (81.3 to 90.4)	88.2 (84.5 to 91.9)
	FAS eGFRcr-cys	9.8 (7.6 to 11.7)	5.0 (3.4 to 6.3)	18.9 (16.0 to 22.5)	17.4 (14.4 to 19.6)	28.8 (22.8 to 34.7)	40.7 (35.4 to 46.5)	77.6 (72.1 to 83.1)	84.2 (79.8 to 88.2)
	FAS-Height eGFRcr-cys	7.7 (5.9 to 10.0)	4.8 (3.5 to 6.5)	19.3 (15.9 to 23.1)	15.5 (12.9 to 19.1)	32.4 (26.5 to 38.8)	43.1 (37.4 to 48.8)	79.0 (73.5 to 84.5)	85.2 (81.1 to 89.2)
	r-LMR eGFRcr-cys	1.6 (-0.6 to 3.8)	3.1 (1.5 to 4.4)	18.2 (14.4 to 21.2)	17.2 (14.7 to 19.0)	43.4 (37.0 to 49.8)	45.5 (39.7 to 51.2)	90.0 (85.8 to 93.6)	87.9 (84.2 to 91.6)

Biases are presented as medians of estimated GFR (eGFR) – measured GFR (mGFR). Similarly, interquartile ranges (IQR) are for the differences of eGFR and mGFR. The P10 and P30 values are the percentage of eGFR values within 10% and 30%, respectively, of the mGFR values. The 95% confidence intervals for the given statistical values were calculated using 107 resamples and are presented in parentheses. Abbreviations of the equations are explained in Supplementary Table S1 and their mathematical expressions in Supplementary Item S1. CI, confidence interval; eGFR, estimated GFR

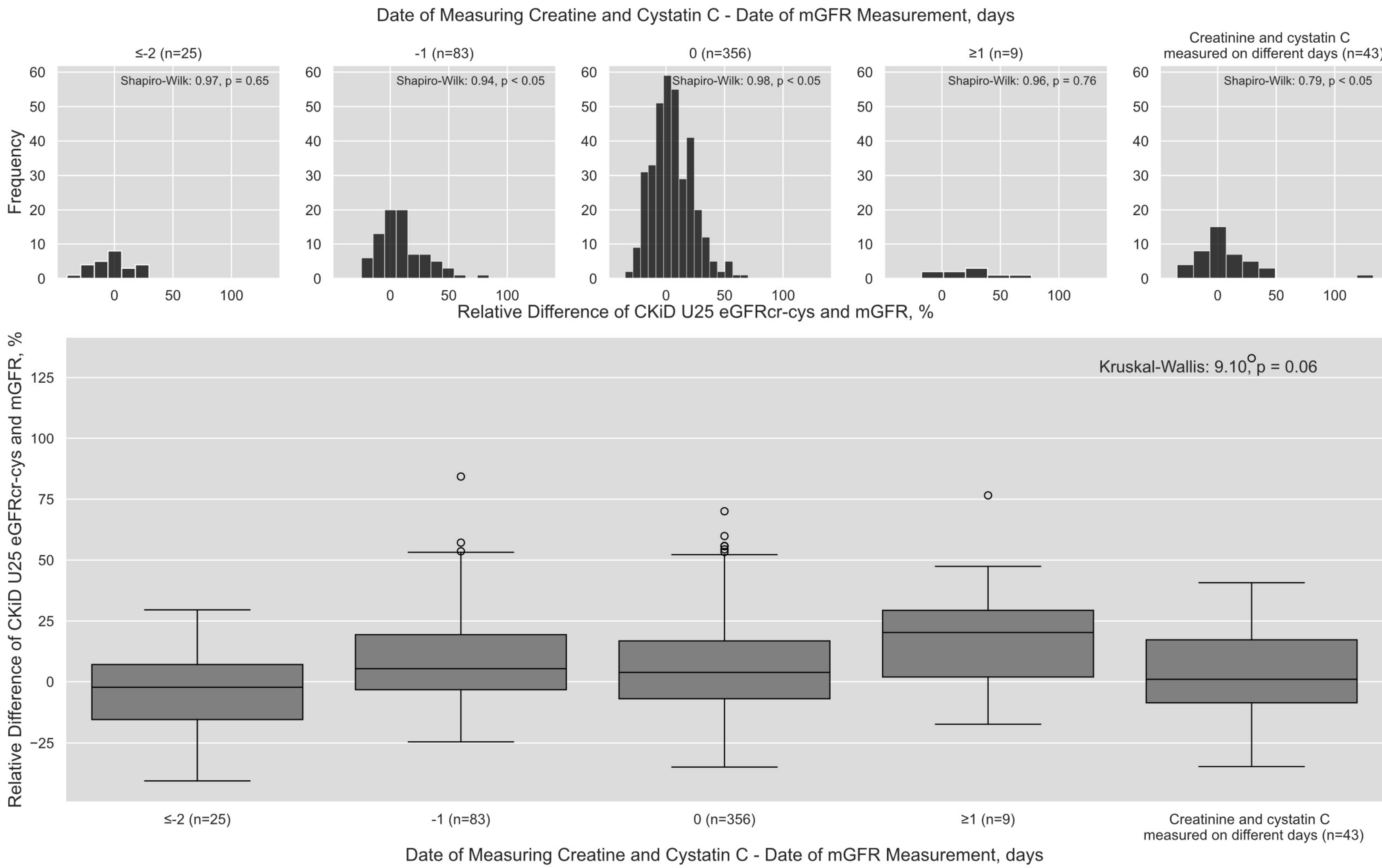

Supplementary Figure S1. Effect of the measurement date difference of laboratory values and mGFR on the relative difference of CKiD U25 eGFRcr-cys and mGFR. The relative difference of eGFR and mGFR ([eGFR – mGFR] / mGFR) is presented in percentages and the measurement date difference in days. The measurement date difference represents the difference of days between the measurement of laboratory values (creatinine and cystatin C) and mGFR. These date differences were grouped to maintain sufficiently large groups for meaningful statistical analysis. The distributions of the relative differences in these groups are presented in the histograms above the boxplot. The solid box shows the quartiles of the dataset with the whiskers extending to the rest of the dataset except the outliers which are presented as points. The horizontal line in the solid box represents the median of the dataset. Shapiro-Wilk test results are presented for each group and the result of the Kruskal-Wallis test is presented for the analysis between all the groups. CKiD, Chronic Kidney Disease in Children Study; CKiD U25 eGFRcr, eGFR based on CKiD under 25 equation using creatinine; CKiD U25 eGFRcr-cys, eGFR based on the mean of CKiD U25 eGFRcr and CKiD U25 eGFRcys equations; CKiD U25 eGFRcys, eGFR based on CKiD under 25 equation using cystatin C; eGFR, estimated GFR; mGFR, measured GFR

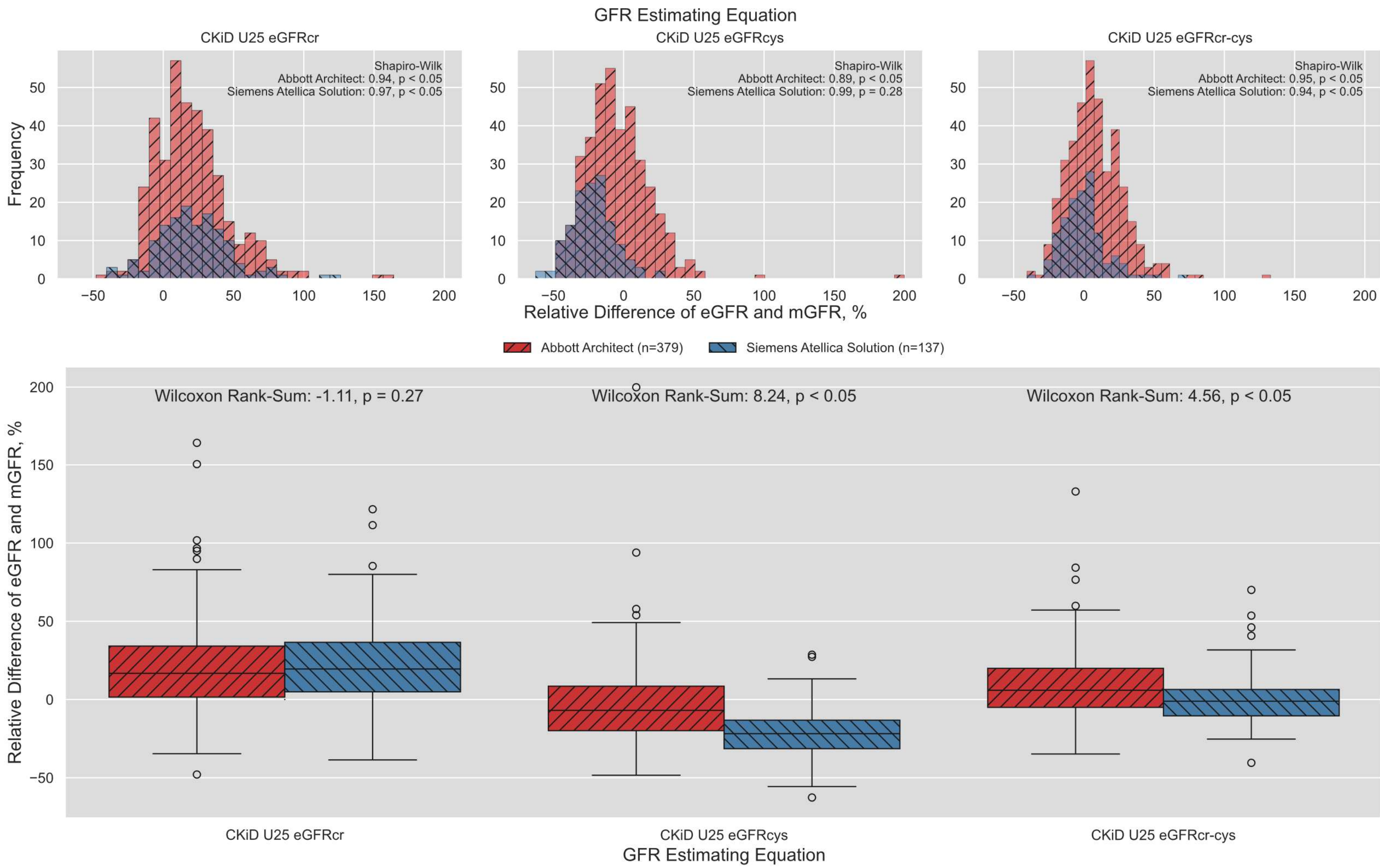

Supplementary Figure S2. Effect of different analyzers on the relative difference of CKiD U25 equations and mGFR. The relative difference of eGFR and mGFR ([eGFR – mGFR] / mGFR) is presented in percentages. Results are presented in different colors depending on the analyzer used for laboratory values. Results using Abbott Architect (n = 379) are presented in red (left) and results using Siemens Atellica Solution (n = 137) are presented in blue (right). The distributions of the relative differences in these groups are presented in the histograms above the boxplot. The solid box shows the quartiles of the dataset with the whiskers extending to the rest of the dataset except the outliers which are presented as points. The horizontal line in the solid box represents the median of the dataset. Shapiro-Wilk test results for both groups and Wilcoxon rank-sum test between the different analyzers are presented for all the analyzed equations. CKiD, Chronic Kidney Disease in Children Study; CKiD U25 eGFRcr, eGFR based on CKiD under 25 equation using creatinine; CKiD U25 eGFRcr-cys, eGFR based on the mean of CKiD U25 eGFRcr and CKiD U25 eGFRcys equations; CKiD U25 eGFRcys, eGFR based on CKiD under 25 equation using cystatin C; eGFR, estimated GFR; mGFR, measured GFR

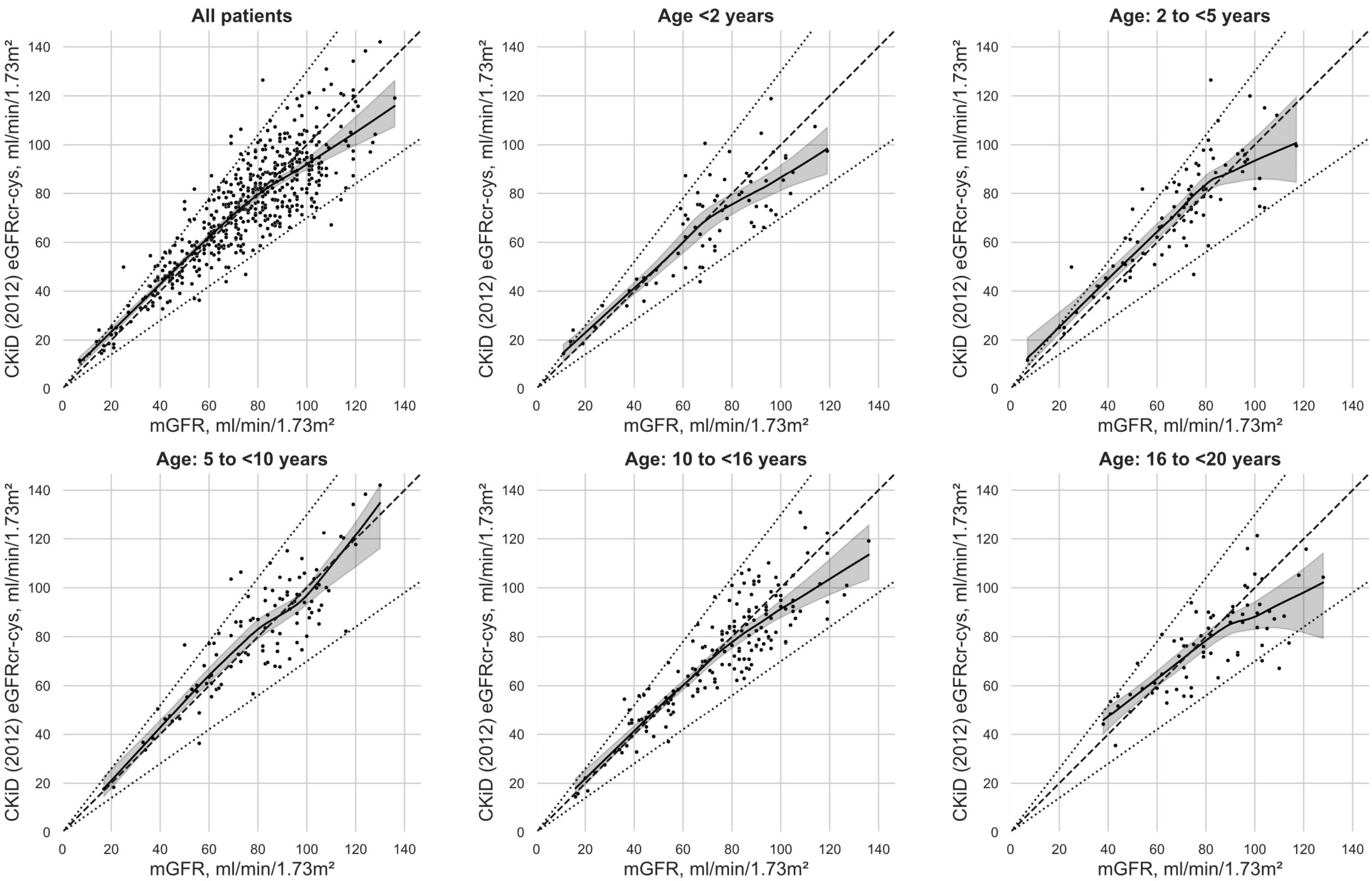

Supplementary Figure S3. Scatterplots with local regression of mGFR and eGFR values for CKiD (2012) eGFRcr-cys equation in all patients as well as in different age groups. The values are presented in ml/min/1.73m^2. The grey area represents the 95% confidence interval for the LOWESS regression (solid line) calculated using bootstrapping (10 000 resamples). The dashed line represents the identity line, and the dotted lines show the lower and higher 30% difference limits of mGFR and eGFR. CKiD, Chronic Kidney Disease in Children Study; CKiD (2012) eGFRcr-cys, eGFR based on CKiD equation (2012) using creatinine, cystatin C and blood urea nitrogen; eGFR, estimated GFR; mGFR, measured GFR

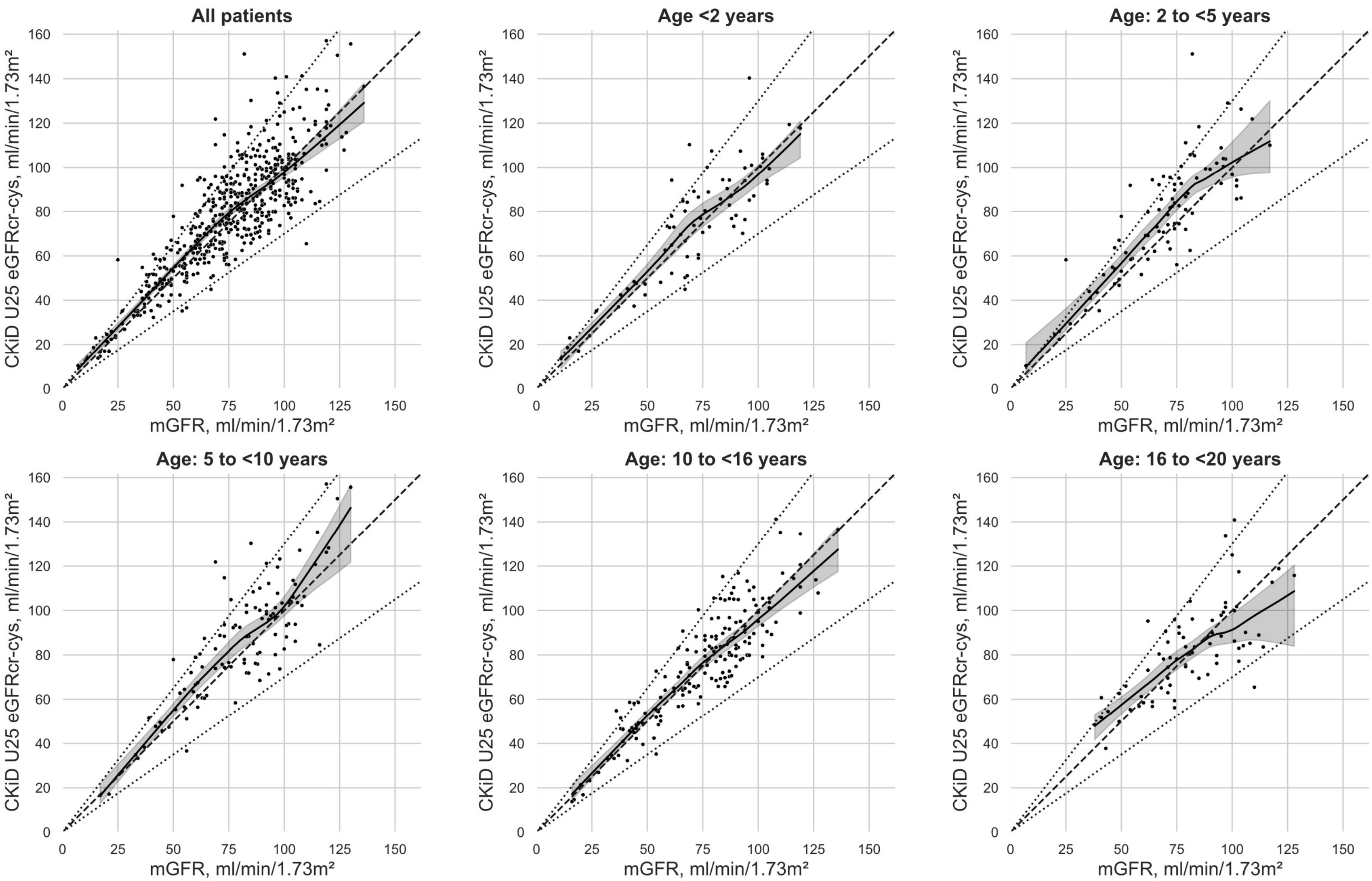

Supplementary Figure S4. Scatterplots with local regression of mGFR and eGFR values for CKiD U25 eGFRcr-cys equation in all patients as well as in different age groups. The values are presented in ml/min/1.73m². The grey area represents the 95% confidence interval for the LOWESS regression (solid line) calculated using bootstrapping (10 000 resamples). The dashed line represents the identity line, and the dotted lines show the lower and higher 30% difference limits of mGFR and eGFR. CKiD, Chronic Kidney Disease in Children Study; CKiD U25 eGFRcr, eGFR based on CKiD under 25 equation using creatinine; CKiD U25 eGFRcr-cys, eGFR based on the mean of CKiD U25 eGFRcr and CKiD U25 eGFRcys equations; CKiD U25 eGFRcys, eGFR based on CKiD under 25 equation using cystatin C; eGFR, estimated GFR; mGFR, measured GFR

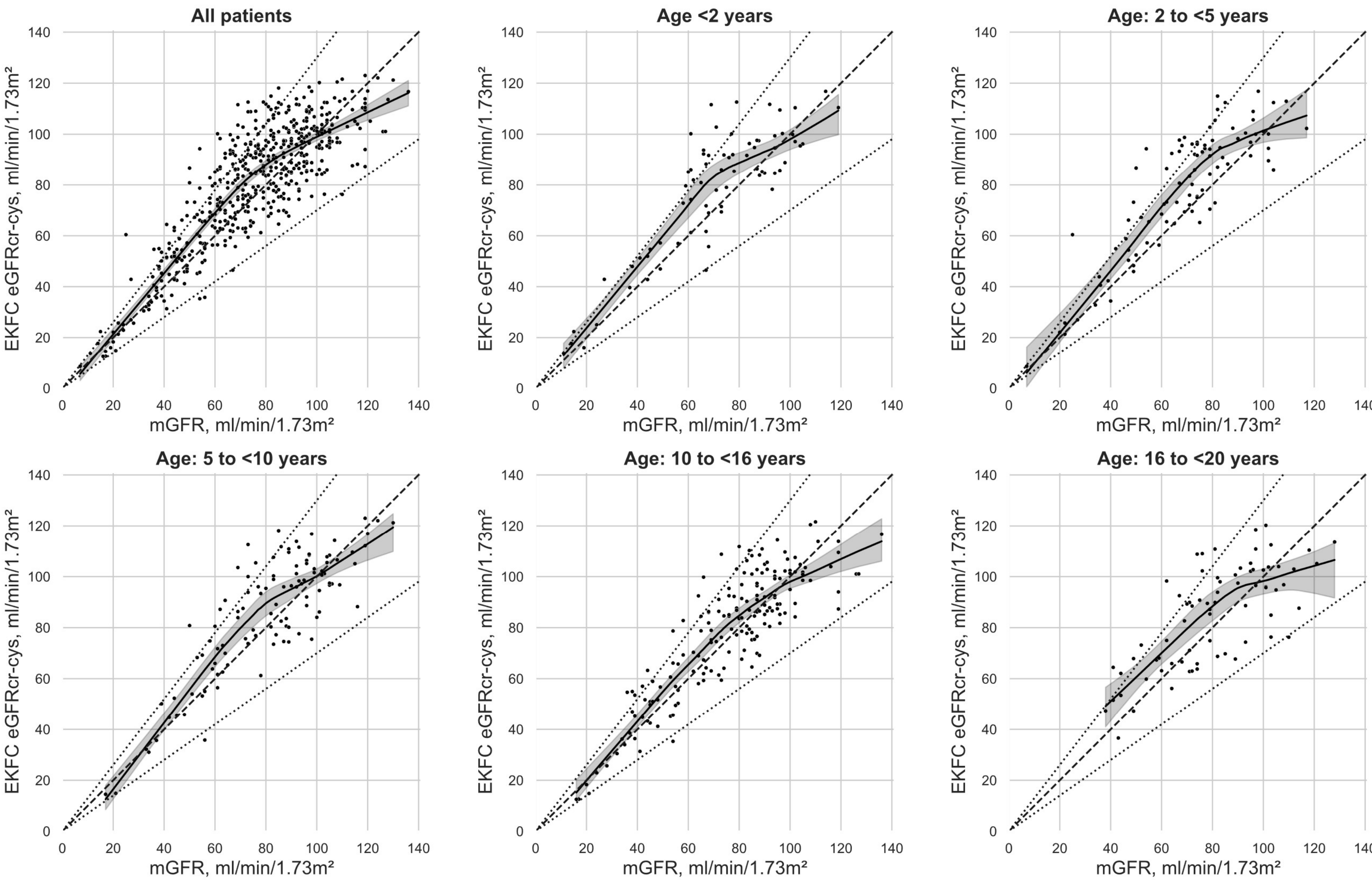

Supplementary Figure S5. Scatterplots with local regression of mGFR and eGFR values for EKFC eGFRcr-cys equation in all patients as well as in different age groups. The values are presented in ml/min/1.73m^2. The grey area represents the 95% confidence interval for the LOWESS regression (solid line) calculated using bootstrapping (10 000 resamples). The dashed line represents the identity line, and the dotted lines show the lower and higher 30% difference limits of mGFR and eGFR. EKFC, European Kidney Function Consortium; EKFC eGFRcr, eGFR based on EKFC equation using creatinine and Q(age); EKFC eGFRcr-cys, eGFR based on the mean of EKFC eGFRcr and EKFC eGFRcys equations; EKFC eGFRcys, eGFR based on EKFC equation using cystatin C; eGFR, estimated GFR; mGFR, measured GFR

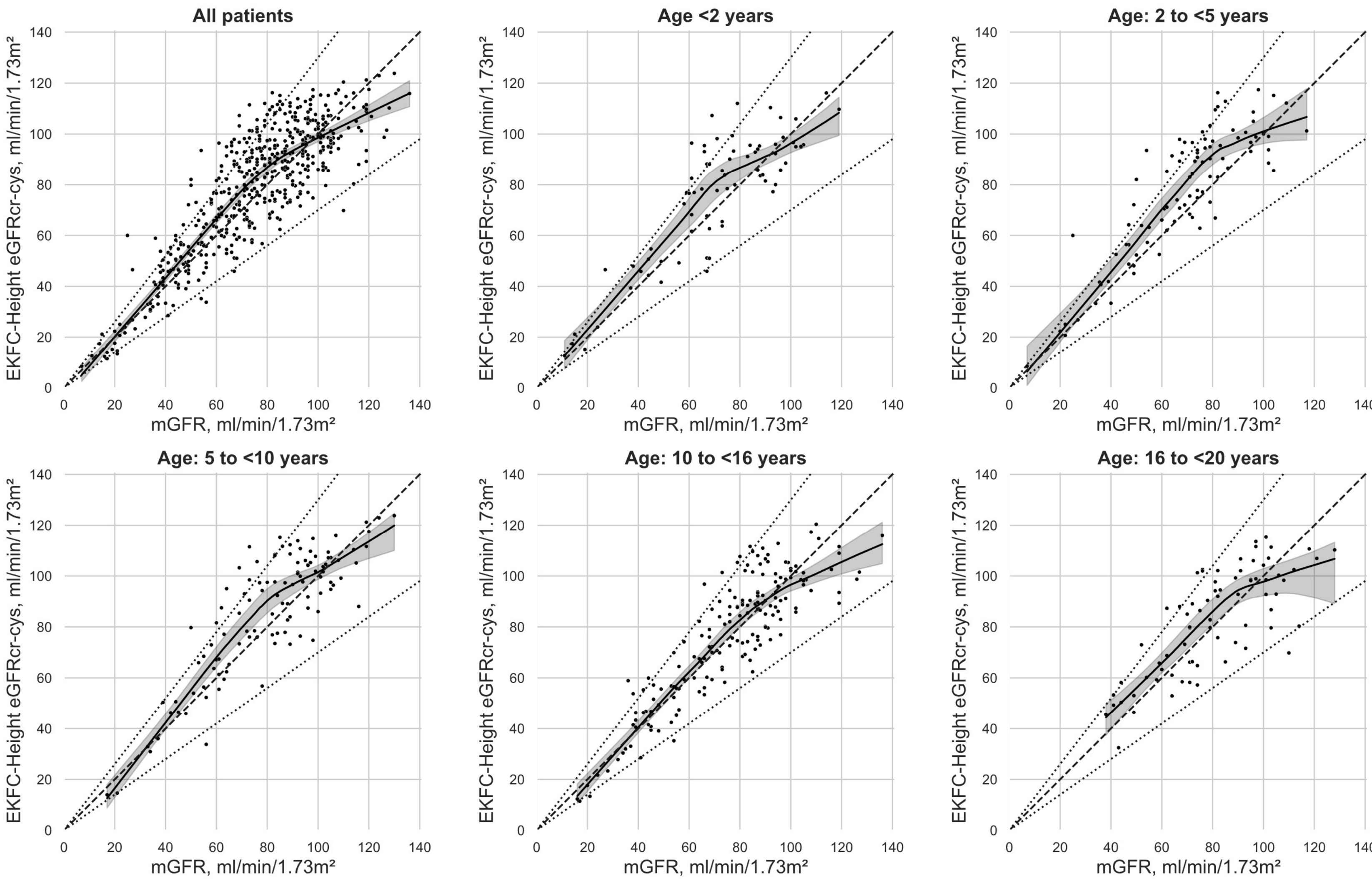

Supplementary Figure S6. Scatterplots with local regression of mGFR and eGFR values for EKFC-Height eGFRcr-cys equation in all patients as well as in different age groups. The values are presented in ml/min/1.73m^2. The grey area represents the 95% confidence interval for the LOWESS regression (solid line) calculated using bootstrapping (10 000 resamples). The dashed line represents the identity line, and the dotted lines show the lower and higher 30% difference limits of mGFR and eGFR. EKFC, European Kidney Function Consortium; EKFC-Height eGFRcr, eGFR based on EKFC equation using creatinine and Q(height); EKFC-Height eGFRcr-cys, eGFR based on the mean of EKFC-Height eGFRcr and EKFC eGFRcys equations; EKFC eGFRcys, eGFR based on EKFC equation using cystatin C; eGFR, estimated GFR; mGFR, measured GFR

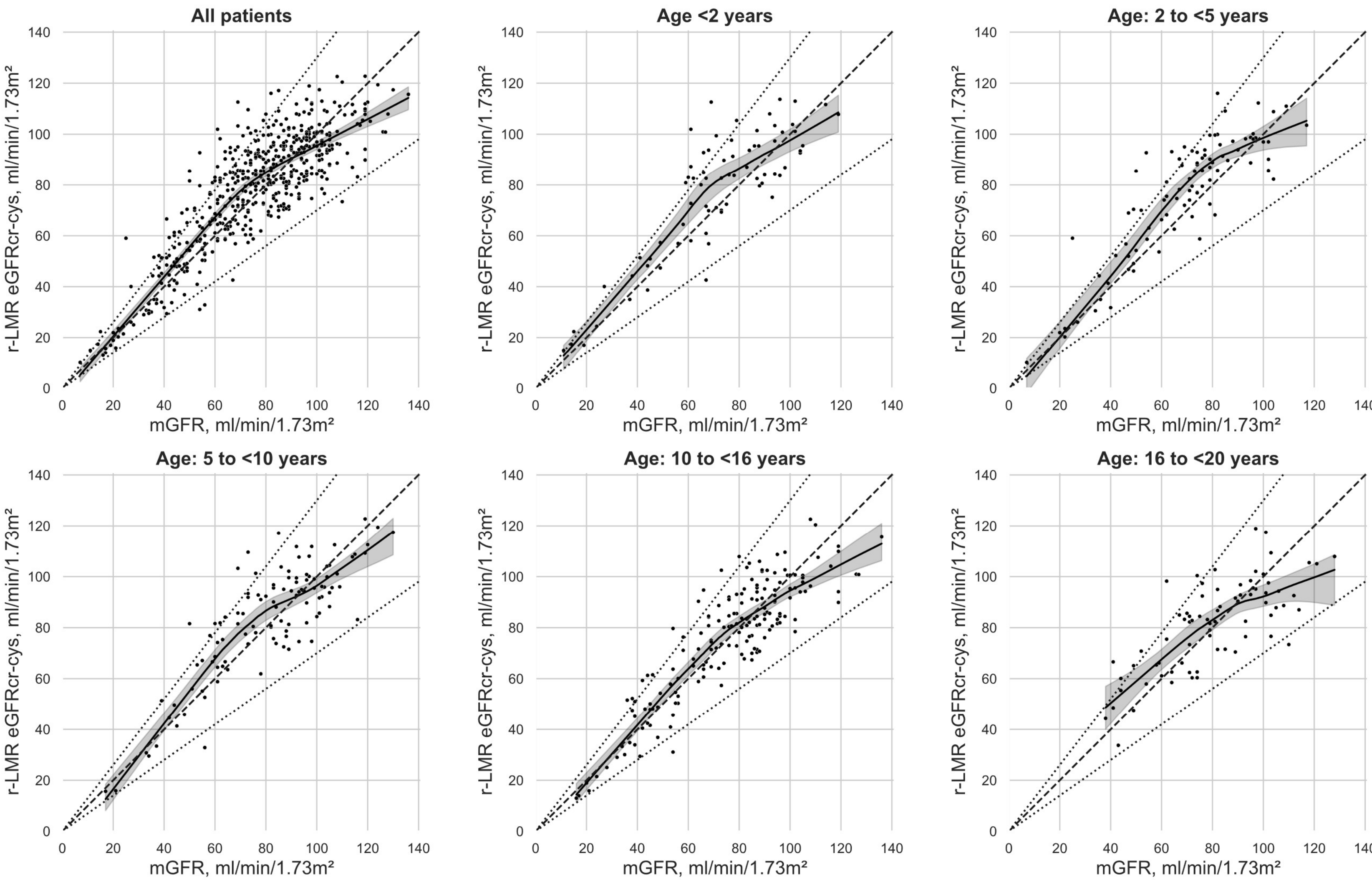

Supplementary Figure S7. Scatterplots with local regression of mGFR and eGFR values for r-LMR eGFRcr-cys equation in all patients as well as in different age groups. The values are presented in ml/min/1.73m^2. The grey area represents the 95% confidence interval for the LOWESS regression (solid line) calculated using bootstrapping (10 000 resamples). The dashed line represents the identity line, and the dotted lines show the lower and higher 30% difference limits of mGFR and eGFR. r-LMR, Lund-Malmö Revised (LMR) equation re-expressed (r-LMR); r-LMR eGFRcr, eGFR based on r-LMR equation using creatinine; r-LMR eGFRcr-cys, eGFR based on the mean of r-LMR eGFRcr and r-LMR eGFRcys equations; r-LMR eGFRcys, eGFR based on r-LMR equation using cystatin C; eGFR, estimated GFR; mGFR, measured GFR

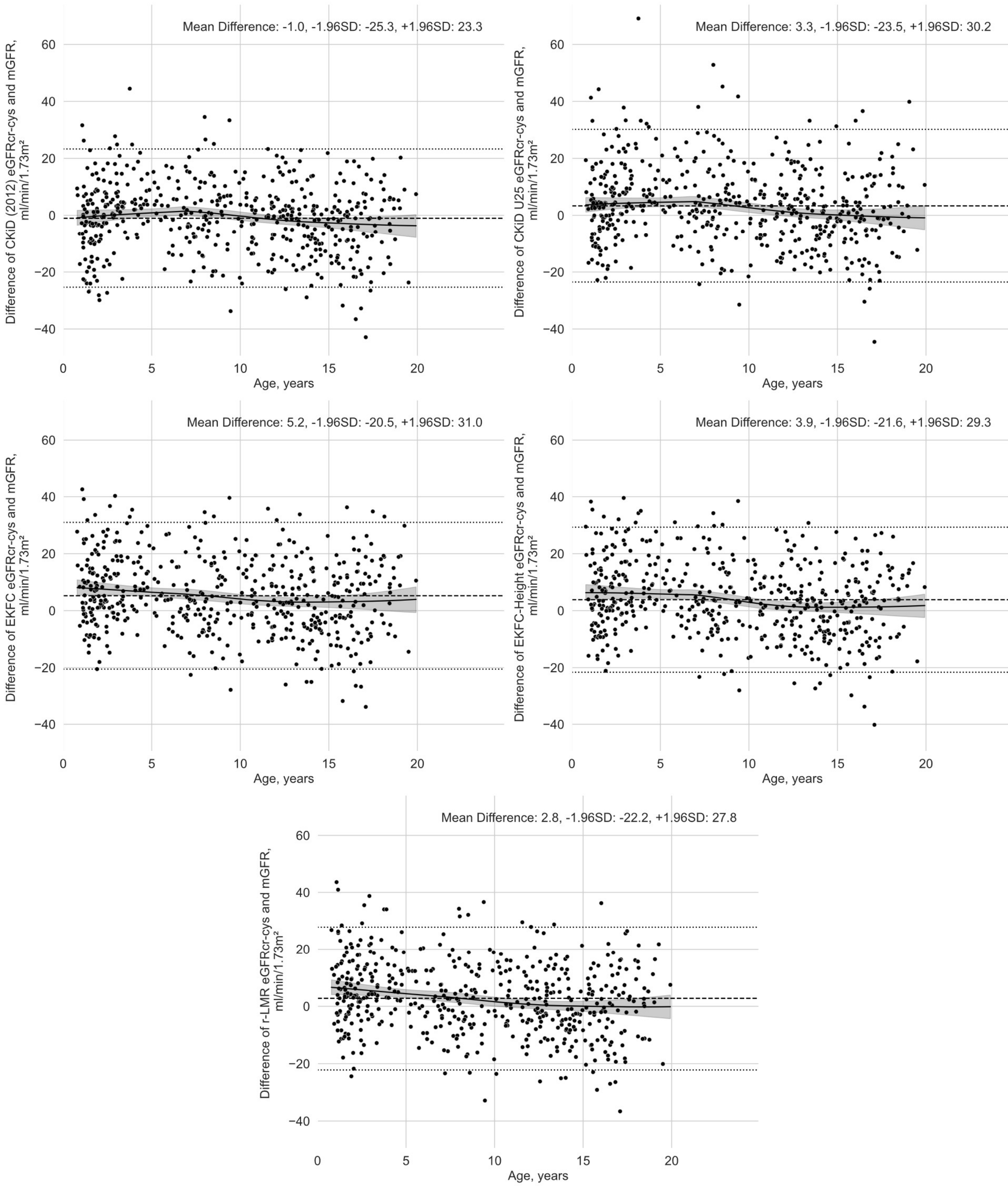

Supplementary Figure S8. Scatterplots with local regression of age and difference of selected combination eGFR equations and mGFR in all patients. The difference of eGFR and mGFR is presented in ml/min/1.73m^2 and age in years. The grey area represents the 95% confidence interval for the LOWESS regression (solid line) calculated using bootstrapping (10 000 resamples). The dashed line represents the mean difference of eGFR equation and mGFR (eGFR – mGFR), and the dotted lines show the ±1.96 standard deviations from the mean difference representing the 95% limits of agreement. Abbreviations of the equations are explained in Supplementary Table S1 and their mathematical expressions in Supplementary Item S1. eGFR, estimated GFR; mGFR, measured GFR; SD, standard deviation

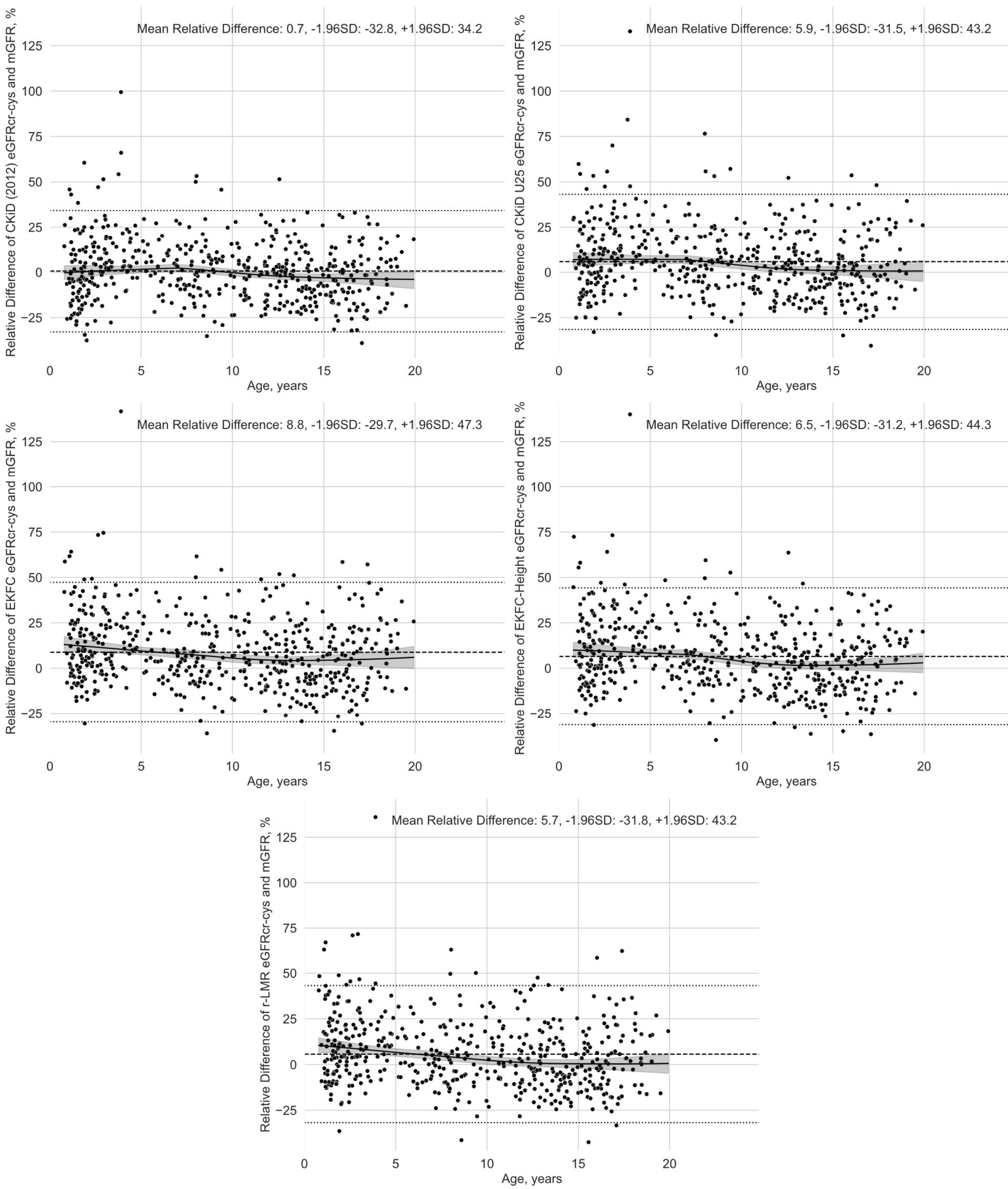

Supplementary Figure S9. Scatterplots with local regression of age and relative difference of selected combination eGFR equations and mGFR in all patients. The relative difference of eGFR and mGFR is presented in percentages and age in years. The grey area represents the 95% confidence interval for the LOWESS regression (solid line) calculated using bootstrapping (10 000 resamples). The dashed line represents the mean relative difference of eGFR equation and mGFR ([eGFR – mGFR] / mGFR), and the dotted lines show the ±1.96 standard deviations from the mean relative difference representing the 95% limits of agreement. Abbreviations of the equations are explained in Supplementary Table S1 and their mathematical expressions in Supplementary Item S1. eGFR, estimated GFR; mGFR, measured GFR; SD, standard deviation

Supplementary Item S1. List of mathematical expressions of analyzed GFR estimating equations

In all equations plasma/serum creatinine (SCr) is expressed in µmol/l, cystatin C (CysC) in mg/l, urea in mmol/l, age in years and height in cm. Some equations include the conversion of SCr from µmol/l to mg/dl by dividing it by 88.4 and the conversion of urea in mmol/l to blood urea nitrogen (BUN) in mg/dl by dividing it by 0.357. Additionally, some equations express height in meters instead of centimeters. However, required unit conversions are detailed under each equation. The CKiD (2012) eGFRcys and CKiD (2012) eGFRcr-cys equations were developed with non-calibrated values of cystatin C but we calculated our estimations using cystatin C traceable to the IFCC reference material.

Creatinine-based equations

CKD-EPI40 eGFRcr

Male: $\ln(SCr_{40}) = \ln(SCr) + 0.259 \times (40 - Age) - 0.543 \times \ln(40 / Age) - 0.00763 \times (40^2 - Age^2) + 0.0000790 \times (40^3 - Age^3)$

Female: $\ln(SCr_{40}) = \ln(SCr) + 0.177 \times (40 - Age) - 0.223 \times \ln(40 / Age) - 0.00596 \times (40^2 - Age^2) + 0.0000686 \times (40^3 - Age^3)$

$SCr_{40} = \exp(\ln(SCr_{40}))$

Male:

$SCr_{40} \leq 80$: $eGFR = 141 \times (SCr_{40} / 80)^{-0.411} \times 0.993^{(\max(Age, 40))}$

$SCr_{40} > 80$: $eGFR = 141 \times (SCr_{40} / 80)^{-1.209} \times 0.993^{(\max(Age, 40))}$

Female:

$SCr_{40} \leq 62$: $eGFR = 144 \times (SCr_{40} / 62)^{-0.329} \times 0.993^{(\max(Age, 40))}$

$SCr_{40} > 62$: $eGFR = 144 \times (SCr_{40} / 62)^{-1.209} \times 0.993^{(\max(Age, 40))}$

CKiD (2009) eGFRcr

$eGFR = 36.5 \times Height / SCr$

CKiD U25 eGFRcr

$SCr = SCr / 88.4$ $Height = Height / 100$

$Age < 12$:

Male: $k = 39.0 \times 1.008^{(Age - 12)}$

Female: $k = 36.1 \times 1.008^{(Age - 12)}$

$12 \leq Age < 18$:

Male: $k = 39.0 \times 1.045^{(Age - 12)}$

Female: $k = 36.1 \times 1.023^{(Age - 12)}$

$18 \leq Age \leq 25$:

Male: $k = 50.8$

Female: $k = 41.4$

$eGFR = k \times (Height / SCr)$

EKFC eGFRcr

$Age \leq 25$:

Male: $\ln(Q) = 3.200 + 0.259 \times Age - 0.543 \times \ln(Age) - 0.00763 \times (Age^2) + 0.0000790 \times (Age^3)$

Female: $\ln(Q) = 3.080 + 0.177 \times Age - 0.223 \times \ln(Age) - 0.00596 \times (Age^2) + 0.0000686 \times (Age^3)$

$Q = \exp(\ln(Q))$

$Age > 25$:

Male: $Q = 80$

Female: $Q = 62$

$Age \leq 40$: $AgeFactor = 1$

$Age > 40$: $AgeFactor = 0.990^{(Age - 40)}$

$SCr / Q < 1.0$: $eGFR = 107.3 \times (SCr / Q)^{-0.322} \times AgeFactor$

$SCr / Q \geq 1.0$: $eGFR = 107.3 \times (SCr / Q)^{-1.132} \times AgeFactor$

EKFC-Height eGFRcr

$SCr = SCr / 88.4$ $Height = Height / 100$

$Height \leq 1.81$: $Q = 3.94 - 13.4 \times Height + 17.6 \times Height^2 - 9.84 \times Height^3 + 2.04 \times Height^4$

$Height > 1.81$: $Q = 0.90$

$Age \leq 40$: $AgeFactor = 1$

$Age > 40$: $AgeFactor = 0.990^{(Age - 40)}$

$SCr / Q < 1.0$: $eGFR = 107.3 \times (SCr / Q)^{-0.322} \times AgeFactor$

$SCr / Q \geq 1.0$: $eGFR = 107.3 \times (SCr / Q)^{-1.132} \times AgeFactor$

FAS eGFRcr

$SCr = SCr / 88.4$

$Age < 20$:

Male: $Q = 0.21 + 0.057 \times Age - 0.0075 \times Age^2 + 0.00064 \times Age^3 - 0.000016 \times Age^4$

Female: $Q = 0.23 + 0.034 \times Age - 0.0018 \times Age^2 + 0.00017 \times Age^3 - 0.0000051 \times Age^4$

$Age \geq 20$:

Male: $Q = 0.90$

Female: $Q = 0.70$
Age ≤ 40: $eGFR = 107.3 / (SCr / Q)$
Age > 40: $eGFR = 107.3 / (SCr / Q) \times 0.988^{(Age - 40)}$

FAS-Height eGFRcr
$SCr = SCr / 88.4$ Height = Height / 100
Age < 20: $Q = 3.94 - 13.4 \times Height + 17.6 \times Height^2 - 9.84 \times Height^3 + 2.04 \times Height^4$
Age ≥ 20:
Male: $Q = 0.90$
Female: $Q = 0.70$
Age ≤ 40: $eGFR = 107.3 / (SCr / Q)$
Age > 40: $eGFR = 107.3 / (SCr / Q) \times 0.988^{(Age - 40)}$

FM eGFRcr
$SCr = SCr / 88.4$
$k = 0.0414 \times \ln(Age) + 0.3018$
$eGFR = k \times Height / SCr$

GQ eGFRcr
$SCr = SCr / 88.4$
Female: SexFactor = 25.68
Male: SexFactor = 21.53
$eGFR = 0.68 \times (Height / SCr) - 0.0008 \times (Height / SCr)^2 + 0.48 \times Age - SexFactor$

LMR18 eGFRcr
Age < 18:
Male: $\ln(SCr_{adj}) = \ln(SCr) + 0.259 \times (18 - Age) - 0.543 \times \ln(18 / Age) - 0.00763 \times (18^2 - Age^2) + 0.0000790 \times (18^3 – Age^3)$
Female: $\ln(SCr_{adj}) = \ln(SCr) + 0.177 \times (18 - Age) - 0.223 \times \ln(18 / Age) - 0.00596 \times (18^2 - Age^2) + 0.0000686 \times (18^3 – Age^3)$
$SCr_{adj} = \exp(\ln(SCr_{adj}))$
Age ≥ 18: $SCr_{adj} = SCr$
Male:
$SCr_{adj} < 180$: $X = 2.56 + 0.00968 \times (180 - SCr_{adj})$
$SCr_{adj} \geq 180$: $X = 2.56 - 0.926 \times \ln(SCr_{adj} / 180)$
Female:
$SCr_{adj} < 150$: $X = 2.50 + 0.0121 \times (150 - SCr_{adj})$
$SCr_{adj} \geq 150$: $X = 2.50 - 0.926 \times \ln(SCr_{adj} / 150)$
$eGFR = \exp(X - 0.0158 \times \max(Age, 18) + 0.438 \times \ln(\max(Age, 18)))$

r-LMR eGFRcr
Age < 18:
Male: $SCr_{adj} = \exp(\ln(SCr) + 0.259 \times (18 - Age) - 0.543 \times \ln(18 / Age) - 0.00763 \times (18^2 - Age^2) + 0.0000790 \times (18^3 – Age^3))$
Female: $SCr_{adj} = \exp(\ln(SCr) + 0.177 \times (18 - Age) - 0.223 \times \ln(18 / Age) - 0.00596 \times (18^2 - Age^2) + 0.0000686 \times (18^3 – Age^3))$
Age ≥ 18: $SCr_{adj} = SCr$
Male: $Q = 80$
Female: $Q = 62$
$SCr_{adj} / Q < 2.33$: $X = 4.3087 - 0.7623 \times SCr_{adj} / Q$
$SCr_{adj} / Q \geq 2.33$: $X = 3.3145 - 0.9260 \times \ln(SCr_{adj} / Q)$
$eGFR = \exp(X - 0.0158 \times \max(Age, 18) + 0.438 \times \ln(\max(Age, 18)))$

Schwartz-Lyon eGFRcr
Age > 13 and Male: $k = 36.5$
Female or (Age ≤ 13 and Male): $k = 32.5$
$eGFR = k \times Height / SCr$

Cystatin C-based equations
Berg eGFRcys
$eGFR = 91 \times CysC^{-1.213}$

CAPA eGFRcys
$eGFR = 130 \times CysC^{-1.069} \times Age^{-0.117} - 7$

CKD-EPI (2012) eGFRcys
Male: SexFactor = 1
Female: SexFactor = 0.932

$eGFR = 133 \times \min(CysC / 0.8, 1)^{-0.499} \times \max(CysC / 0.8, 1)^{-1.328} \times (0.996^{Age}) \times SexFactor$

CKiD (2012) eGFRcys

$eGFR = 70.69 \times CysC^{-0.931}$

CKiD U25 eGFRcys

Age < 12:
- Male: $k = 87.2 \times 1.011^{(Age - 15)}$
- Female: $k = 79.9 \times 1.004^{(Age - 12)}$

12 ≤ Age ≤ 15:
- Male: $k = 87.2 \times 1.011^{(Age - 15)}$
- Female: $k = 79.9 \times 0.974^{(Age - 12)}$

15 ≤ Age < 18:
- Male: $k = 87.2 \times 0.960^{(Age - 15)}$
- Female: $k = 79.9 \times 0.974^{(Age - 12)}$

18 ≤ Age ≤ 25:
- Male: $k = 77.1$
- Female: $k = 68.3$

$eGFR = k \times (1 / CysC)$

EKFC eGFRcys

Age ≤ 50: $Q = 0.83$
Age > 50: $Q = 0.83 + 0.005 \times (Age - 50)$
Age ≤ 40: $AgeFactor = 1$
Age > 40: $AgeFactor = 0.990^{(Age - 40)}$
CysC / Q < 1.0: $eGFR = 107.3 \times (CysC / Q)^{-0.322} \times AgeFactor$
CysC / Q ≥ 1.0: $eGFR = 107.3 \times (CysC / Q)^{-1.132} \times AgeFactor$

FAS eGFRcys

Age < 70: $Q = 0.82$
Age ≥ 70: $Q = 0.95$
Age ≤ 40: $eGFR = 107.3 / (CysC / Q)$
Age > 40: $eGFR = 107.3 / (CysC / Q) \times 0.988^{(Age - 40)}$

r-LMR eGFRcys

Age ≤ 50: $Q_{CysC} = 0.83$
Age > 50: $Q_{CysC} = 0.83 + 0.005 \times (Age - 50)$
Age < 18:

Male:
$Q_{SCr} = \exp(3.200 + (0.259 \times Age) - 0.543 \times \ln(Age) - 0.00763 \times (Age^2) + 0.0000790 \times (Age^3))$
$SCr_{transl} = (CysC / Q_{CysC}) \times Q_{SCr}$
$SCr_{adj} = \exp(\ln(SCr_{transl}) + 0.259 \times (18 - Age) - 0.543 \times \ln(18 / Age) - 0.00763 \times (18^2 - Age^2) + 0.0000790 \times (18^3 - Age^3))$
$Q_{SCr18} = 80$

Female:
$Q_{SCr} = \exp(3.080 + (0.177 \times Age) - 0.223 \times \ln(Age) - 0.00596 \times (Age^2) + 0.0000686 \times (Age^3))$
$SCr_{transl} = (CysC / Q_{CysC}) \times Q_{SCr}$
$SCr_{adj} = \exp(\ln(SCr_{transl}) + 0.177 \times (18 - Age) - 0.223 \times \ln(18 / Age) - 0.00596 \times (18^2 - Age^2) + 0.0000686 \times (18^3 - Age^3))$
$Q_{SCr18} = 62$

$SCr_{adj} / Q_{SCr18} < 2.33$: $X = 4.3087 - 0.7623 \times SCr_{adj} / Q_{SCr18}$
$SCr_{adj} / Q_{SCr18} \geq 2.33$: $X = 3.3145 - 0.9260 \times \ln(SCr_{adj} / Q_{SCr18})$
$eGFR = \exp(X - 0.0158 \times 18 + 0.438 \times \ln(18))$

Age ≥ 18:

$CysC / Q_{CysC} < 2.33$: $X = 4.3087 - 0.7623 \times CysC / Q_{CysC}$
$CysC / Q_{CysC} \geq 2.33$: $X = 3.3145 - 0.9260 \times \ln(CysC / Q_{CysC})$
$eGFR = \exp(X - 0.0158 \times Age + 0.438 \times \ln(Age))$

Creatinine-Cystatin C-based combination equations

CKiD (2012) eGFRcr-cys

$SCr = SCr / 88.4$ $BUN = Urea / 0.357$ $Height = Height / 100$
Male: $SexFactor = 1.076$
Female: $SexFactor = 1$
$eGFR = 39.8 \times (Height / SCr)^{0.456} \times (1.8 / CysC)^{0.418} \times (30 / BUN)^{0.079} \times SexFactor \times (Height / 1.4)^{0.179}$

CKiD U25 eGFRcr-cys
eGFR = (CKiD U25 eGFRcr + CKiD U25 eGFRcys) / 2

EKFC eGFRcr-cys
eGFR = (EKFC eGFRcr + EKFC eGFRcys) / 2

EKFC-Height eGFRcr-cys
eGFR = (EKFC-Height eGFRcr + EKFC eGFRcys) / 2

FAS eGFRcr-cys
SCr = SCr / 88.4
Age < 20:
Male: $Q_{SCr} = 0.21 + 0.057 \times Age - 0.0075 \times Age^2 + 0.00064 \times Age^3 - 0.000016 \times Age^4$
Female: $Q_{SCr} = 0.23 + 0.034 \times Age - 0.0018 \times Age^2 + 0.00017 \times Age^3 - 0.0000051 \times Age^4$
Age ≥ 20:
Male: $Q_{SCr} = 0.90$
Female: $Q_{SCr} = 0.70$
Age < 70: $Q_{CysC} = 0.82$
Age ≥ 70: $Q_{CysC} = 0.95$
$\alpha = 0.5$
Age ≤ 40: $eGFR = 107.3 / (\alpha \times (SCr / Q_{SCr}) + (1 - \alpha) \times (CysC / Q_{CysC}))$
Age > 40: $eGFR = 107.3 / (\alpha \times (SCr / Q_{SCr}) + (1 - \alpha) \times (CysC / Q_{CysC})) \times 0.988^{(Age - 40)}$

FAS-Height eGFRcr-cys
SCr = SCr / 88.4 Height = Height / 100
Age < 20: $Q_{SCr} = 3.94 - 13.4 \times Height + 17.6 \times Height^2 - 9.84 \times Height^3 + 2.04 \times Height^4$
Age ≥ 20:
Male: $Q_{SCr} = 0.90$
Female: $Q_{SCr} = 0.70$
Age < 70: $Q_{CysC} = 0.82$
Age ≥ 70: $Q_{CysC} = 0.95$
$\alpha = 0.5$
Age ≤ 40: $eGFR = 107.3 / (\alpha \times (SCr / Q_{SCr}) + (1 - \alpha) \times (CysC / Q_{CysC}))$
Age > 40: $eGFR = 107.3 / (\alpha \times (SCr / Q_{SCr}) + (1 - \alpha) \times (CysC / Q_{CysC})) \times 0.988^{(Age - 40)}$

r-LMR eGFRcr-cys
eGFR = (r-LMR eGFRcr + r-LMR eGFRcys) / 2

Supplementary Item S2. STARD 2015 Checklist

Section & Topic	No	Item	Reported on page #
**TITLE OR ABSTRACT**			
	**1**	Identification as a study of diagnostic accuracy using at least one measure of accuracy (such as sensitivity, specificity, predictive values, or AUC)	p. 2
**ABSTRACT**			
	**2**	Structured summary of study design, methods, results, and conclusions (for specific guidance, see STARD for Abstracts)	p. 2
**INTRODUCTION**			
	**3**	Scientific and clinical background, including the intended use and clinical role of the index test	pp. 3–4
	**4**	Study objectives and hypotheses	p. 4
**METHODS**			
Study design	**5**	Whether data collection was planned before the index test and reference standard were performed (prospective study) or after (retrospective study)	p. 4
Participants	**6**	Eligibility criteria	p. 4
	**7**	On what basis potentially eligible participants were identified (such as symptoms, results from previous tests, inclusion in registry)	pp. 4–5
	**8**	Where and when potentially eligible participants were identified (setting, location and dates)	pp. 4–5
	**9**	Whether participants formed a consecutive, random or convenience series	p. 5
Test methods	**10a**	Index test, in sufficient detail to allow replication	pp. 6–7, Table S1 and Item S1
	**10b**	Reference standard, in sufficient detail to allow replication	pp. 5-6
	**11**	Rationale for choosing the reference standard (if alternatives exist)	pp. 3–6
	**12a**	Definition of and rationale for test positivity cut-offs or result categories of the index test, distinguishing pre-specified from exploratory	p. 7
	**12b**	Definition of and rationale for test positivity cut-offs or result categories of the reference standard, distinguishing pre-specified from exploratory	p. 7
	**13a**	Whether clinical information and reference standard results were available to the performers/readers of the index test	NA
	**13b**	Whether clinical information and index test results were available to the assessors of the reference standard	NA
Analysis	**14**	Methods for estimating or comparing measures of diagnostic accuracy	pp. 7–8
	**15**	How indeterminate index test or reference standard results were handled	NA
	**16**	How missing data on the index test and reference standard were handled	p. 5
	**17**	Any analyses of variability in diagnostic accuracy, distinguishing pre-specified from exploratory	pp. 7–8
	**18**	Intended sample size and how it was determined	p. 5
**RESULTS**			
Participants	**19**	Flow of participants, using a diagram	p. 5 and Figure 1
	**20**	Baseline demographic and clinical characteristics of participants	pp. 8–9 and Tables 1, S8, S10
	**21a**	Distribution of severity of disease in those with the target condition	pp. 8–9 and Tables 1, S8, S10
	**21b**	Distribution of alternative diagnoses in those without the target condition	pp. 8–9 and Tables 1, S8, S10
	**22**	Time interval and any clinical interventions between index test and reference standard	p. 9, Figure S1
Test results	**23**	Cross tabulation of the index test results (or their distribution) by the results of the reference standard	NA

	**24**	Estimates of diagnostic accuracy and their precision (such as 95% confidence intervals)	pp. 9–12, Tables 2–4, S2–7, S9, S11 and Figures 2, S3–9
	**25**	Any adverse events from performing the index test or the reference standard	NA
**DISCUSSION**			
	**26**	Study limitations, including sources of potential bias, statistical uncertainty, and generalisability	p. 16–17
	**27**	Implications for practice, including the intended use and clinical role of the index test	pp. 12–17
**OTHER INFORMATION**			
	**28**	Registration number and name of registry	NA
	**29**	Where the full study protocol can be accessed	NA
	**30**	Sources of funding and other support; role of funders	p. 18

STARD 2015

AIM

STARD stands for "Standards for Reporting Diagnostic accuracy studies". This list of items was developed to contribute to the completeness and transparency of reporting of diagnostic accuracy studies. Authors can use the list to write informative study reports. Editors and peer-reviewers can use it to evaluate whether the information has been included in manuscripts submitted for publication.

Explanation

A **diagnostic accuracy study** evaluates the ability of one or more medical tests to correctly classify study participants as having a **target condition.** This can be a disease, a disease stage, response or benefit from therapy, or an event or condition in the future. A medical test can be an imaging procedure, a laboratory test, elements from history and physical examination, a combination of these, or any other method for collecting information about the current health status of a patient.

The test whose accuracy is evaluated is called **index test.** A study can evaluate the accuracy of one or more index tests. Evaluating the ability of a medical test to correctly classify patients is typically done by comparing the distribution of the index test results with those of the **reference standard**. The reference standard is the best available method for establishing the presence or absence of the target condition. An accuracy study can rely on one or more reference standards.

If test results are categorized as either positive or negative, the cross tabulation of the index test results against those of the reference standard can be used to estimate the **sensitivity** of the index test (the proportion of participants *with* the target condition who have a positive index test), and its **specificity** (the proportion *without* the target condition who have a negative index test). From this cross tabulation (sometimes referred to as the contingency or "2x2" table), several other accuracy statistics can be estimated, such as the positive and negative **predictive values** of the test. Confidence intervals around estimates of accuracy can then be calculated to quantify the statistical **precision** of the measurements.

If the index test results can take more than two values, categorization of test results as positive or negative requires a **test positivity cut-off**. When multiple such cut-offs can be defined, authors can report a receiver operating characteristic (ROC) curve which graphically represents the combination of sensitivity and specificity for each possible test positivity cut-off. The **area under the ROC curve** informs in a single numerical value about the overall diagnostic accuracy of the index test.

The **intended use** of a medical test can be diagnosis, screening, staging, monitoring, surveillance, prediction or prognosis. The **clinical role** of a test explains its position relative to existing tests in the clinical pathway. A replacement test, for example, replaces an existing test. A triage test is used before an existing test; an add-on test is used after an existing test.

Besides diagnostic accuracy, several other outcomes and statistics may be relevant in the evaluation of medical tests. Medical tests can also be used to classify patients for purposes other than diagnosis, such as staging or prognosis. The STARD list was not explicitly developed for these other outcomes, statistics, and study types, although most STARD items would still apply.

DEVELOPMENT

This STARD list was released in 2015. The 30 items were identified by an international expert group of methodologists, researchers, and editors. The guiding principle in the development of STARD was to select items that, when reported, would help readers to judge the potential for bias in the study, to appraise the applicability of the study findings and the validity of conclusions and recommendations. The list represents an update of the first version, which was published in 2003.

More information can be found on http://www.equator-network.org/reporting-guidelines/stard.

Supplementary References

S1. Björk J, Nyman U, Larsson A, Delanaye P, Pottel H. Estimation of the glomerular filtration rate in children and young adults by means of the CKD-EPI equation with age-adjusted creatinine values. *Kidney Int*. 2021;99(4):940-947. doi:10.1016/j.kint.2020.10.017

S2. Hoste L, Dubourg L, Selistre L, et al. A new equation to estimate the glomerular filtration rate in children, adolescents and young adults. *Nephrol Dial Transplant*. 2014;29(5):1082-1091. doi:10.1093/ndt/gft277

S3. Pottel H, Hoste L, Dubourg L, et al. An estimated glomerular filtration rate equation for the full age spectrum. *Nephrol Dial Transplant*. 2016;31(5):798-806. doi:10.1093/ndt/gfv454

S4. Pottel H, Mottaghy FM, Zaman Z, Martens F. On the relationship between glomerular filtration rate and serum creatinine in children. *Pediatr Nephrol*. 2010;25(5):927-934. doi:10.1007/s00467-009-1389-1

S5. Gao A, Cachat F, Faouzi M, et al. Comparison of the glomerular filtration rate in children by the new revised Schwartz formula and a new generalized formula. *Kidney Int*. 2013;83(3):524-530. doi:10.1038/ki.2012.388

S6. Björk J, Nyman U, Delanaye P, et al. A novel method for creatinine adjustment makes the revised Lund-Malmö GFR estimating equation applicable in children. *Scand J Clin Lab Invest*. 2020;80(6):456-463. doi:10.1080/00365513.2020.1774641

S7. De Souza VC, Rabilloud M, Cochat P, et al. Schwartz formula: is one k-coefficient adequate for all children? *PLoS One*. 2012;7(12):e53439. doi:10.1371/journal.pone.0053439

S8. Berg UB, Nyman U, Bäck R, et al. New standardized cystatin C and creatinine GFR equations in children validated with inulin clearance. *Pediatr Nephrol*. 2015;30(8):1317-1326. doi:10.1007/s00467-015-3060-3

S9. Grubb A, Horio M, Hansson LO, et al. Generation of a new cystatin C-based estimating equation for glomerular filtration rate by use of 7 assays standardized to the international calibrator. *Clin Chem*. 2014;60(7):974-986. doi:10.1373/clinchem.2013.220707

S10. Inker LA, Schmid CH, Tighiouart H, et al. Estimating glomerular filtration rate from serum creatinine and cystatin C. *N Engl J Med*. 2012;367(1):20-29. doi:10.1056/NEJMoa1114248

S11. Pottel H, Delanaye P, Schaeffner E, et al. Estimating glomerular filtration rate for the full age spectrum from serum creatinine and cystatin C. *Nephrol Dial Transplant*. 2017;32(3):497-507. doi:10.1093/ndt/gfw425
